# A benchmark of batch-effect correction methods for single-cell RNA sequencing data

**DOI:** 10.1186/s13059-019-1850-9

**Published:** 2020-01-16

**Authors:** Hoa Thi Nhu Tran, Kok Siong Ang, Marion Chevrier, Xiaomeng Zhang, Nicole Yee Shin Lee, Michelle Goh, Jinmiao Chen

**Affiliations:** Singapore Immunology Network (SIgN), Agency for Science, Technology and Research (A*STAR), 8A Biomedical Grove, Immunos Building, Level 3, Singapore, 138648 Singapore

**Keywords:** Single-cell RNA-seq, Batch correction, Batch effect, Integration, Differential gene expression

## Abstract

**Background:**

Large-scale single-cell transcriptomic datasets generated using different technologies contain batch-specific systematic variations that present a challenge to batch-effect removal and data integration. With continued growth expected in scRNA-seq data, achieving effective batch integration with available computational resources is crucial. Here, we perform an in-depth benchmark study on available batch correction methods to determine the most suitable method for batch-effect removal.

**Results:**

We compare 14 methods in terms of computational runtime, the ability to handle large datasets, and batch-effect correction efficacy while preserving cell type purity. Five scenarios are designed for the study: identical cell types with different technologies, non-identical cell types, multiple batches, big data, and simulated data. Performance is evaluated using four benchmarking metrics including kBET, LISI, ASW, and ARI. We also investigate the use of batch-corrected data to study differential gene expression.

**Conclusion:**

Based on our results, Harmony, LIGER, and Seurat 3 are the recommended methods for batch integration. Due to its significantly shorter runtime, Harmony is recommended as the first method to try, with the other methods as viable alternatives.

## Introduction

Technological advances in the recent years have increased our ability to generate high-throughput single-cell gene expression data. Single-cell data is often compiled from multiple experiments with differences in capturing times, handling personnel, reagent lots, equipments, and even technology platforms. These differences lead to large variations or batch effects in the data, and can confound biological variations of interest during data integration. As such, effective batch-effect removal is essential. Batch effects can be highly nonlinear, making it difficult to correctly align different datasets while preserving key biological variations. To address these challenges, tools developed for microarray data batch correction such as ComBat [1] and limma [2] have been employed on single-cell RNA-seq (scRNA-seq) data. However, single-cell experiments suffer from “drop out” events due to the stochasticity of gene expression, or failure in RNA capture or amplification during sequencing [3]. This has prompted efforts to develop workflows to handle data with such characteristics [4–6].

A popular and successful approach, pioneered by Haghverdi et al. [5], identifies cell mappings between datasets and then reconstructs the data in a shared space. The algorithm first identifies mutual nearest neighbors (MNNs) to establish connections between two datasets. The resulting list of paired cells (or MNNs) is used to compute the translation vector to align the datasets into a shared space. The advantage of this approach is that a normalized gene expression matrix is obtained, which can be employed in downstream analysis. However, this approach is computationally demanding in terms of CPU time and memory, due to the need to compute the list of neighbors in a high dimension gene expression space. As such, the developers introduced fastMNN [5, 7], which applies the MNN scheme in the subspace computed using principal component analysis (PCA) [8], resulting in significant improvements in both runtime and accuracy. Two other methods, Scanorama [9] and BBKNN [10], also search for MNNs in dimensionally reduced spaces and use them in a similarity weighted manner to guide batch integration.

The Seurat MultiCCA method from the popular Seurat package was developed in 2017 by the Satija lab [4]. It employs canonical correlation analysis (CCA) [11] to reduce data dimensionality and capture the most correlated data features to align the data batches. A newer version, Seurat Integration (Seurat 3) [12], first uses CCA to project the data into a subspace to identify correlations across datasets. The MNNs are then computed in the CCA subspace and serve as “anchors” to correct the data. Another recently proposed method, Harmony [13], first employs PCA for dimensionality reduction. In the PCA space, Harmony iteratively removes batch effects present. At each iteration, it clusters similar cells from different batches while maximizing the diversity of batches within each cluster and then calculates a correction factor for each cell to be applied. This approach is fast and can accurately detect the true biological connection across datasets.

LIGER is a newly developed method to handle a perceived shortcoming of other methods, which is the assumption that differences between datasets are entirely due to technical variations and not of biological origins, thus aiming to remove all of them [14]. LIGER uses integrative non-negative matrix factorization to first obtain a low-dimensional representation of the input data. The representation is composed of two parts: a set of batch-specific factors and a set of shared factors. Thereafter, clustering is performed and followed by a search for shared clusters using a shared factor neighborhood graph to connect cells with similar neighborhoods. With the identified clusters, the factor loading quantiles are then normalized to match a chosen reference dataset (typically the set with the largest number of cells), thus accomplishing batch correction.

The field of deep neural networks in machine learning has experienced tremendous progress in recent years. Taking advantage of these developments, researchers have started to apply neural networks to batch alignment problems, giving rise to alternate approaches in batch correction. For example, Shaham et al. [15] trained residual neural networks for batch correction by minimizing the maximum mean discrepancy between the distributions of source and target batches. This approach uses the training data to learn a map from source data to target data; as such, it has comparatively poorer performance with small datasets. Lotfollahi et al. [16] developed scGen, where a variational autoencoder (VAE) model is trained on a reference dataset before being used to correct the actual data [17]. The VAE model compared favorably against other models such as Generative Adversarial Network in batch correction applications. Similar to MNN Correct, scGen returns a normalized gene expression matrix, which is useful for downstream analysis.

Studies that comprehensively compare the performance of various batch correcting algorithms are currently lacking. While publications describing new methods do benchmark against existing approaches, these comparisons are often against a small number of methods in limited scenarios with a small number of datasets [4, 5, 9–13, 16]. Furthermore, the comparisons are often made by visual inspection, which itself is subject to different interpretations. Assessment metrics such as average silhouette width (ASW) or adjusted rand index (ARI) are sometimes used, but as demonstrated in our study, may not give a full picture of batch correction result. As such, our present study aims to comprehensively evaluate all batch-effect correction approaches that have been developed for RNA-seq data to date in an objective manner with the aid of multiple evaluation metrics. Specifically, we test the following methods: MNN Correct [5], fastMNN [5, 7], MultiCCA Seurat 2 [4], Seurat 3 [12], MMD-ResNet [15], Harmony [13], Scanorama [9], BBKNN [10], scGen [16], ComBat [1], LIGER [14], limma [2], scMerge [18], and ZINB-WaVE [6]. We employ ten datasets with different characteristics in order to test these methods under five different scenarios. These scenarios are as follows: batches with identical cell types but different sequencing technologies, batches containing non-identical cell types, multiple batches, big datasets with more than half a million cells, and simulated datasets for differential gene expression analysis. The last scenario explores the impact of batch correction on differentially expressed genes.

## Results

### Comprehensive benchmarking of 14 methods on ten datasets using five evaluation metrics

**Fig. 1:**
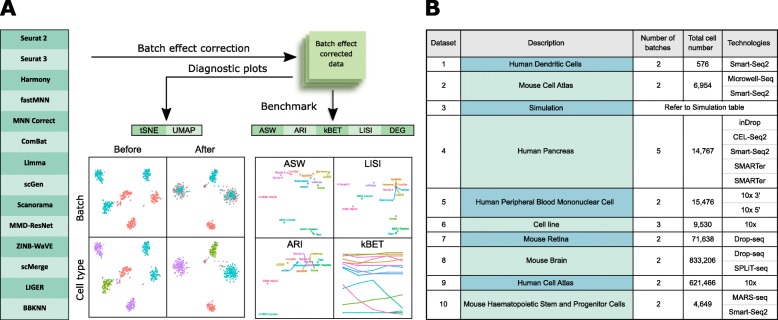
Benchmarking 14 methods on ten datasets using five evaluation metrics. **a** Benchmarking workflow. We evaluated the performance of 14 batch correcting algorithms in terms of their ability to integrate batches while maintaining accuracy in terms of cell type separation. We employed t-SNE and UMAP visualizations in conjunction with the kBET, LISI, ASW, ARI, and DEG benchmarking metrics to evaluate the batch correction results. **b** Description of the ten datasets on which the batch correction algorithms were tested

We evaluated the performance of batch correcting algorithms in terms of their ability to integrate batches while maintaining cell type separation (Fig. 1). The batch correction algorithms tested are either available in the R or Python language environment. For Seurat 2, Harmony, MNN Correct, fastMNN, and limma, the data preprocessing steps of normalization, scaling, and highly variable gene (HVG) selection were performed using the Seurat 2 package. For Seurat 3 batch correction, the corresponding functions in the package were used. For the other methods, their respective recommended preprocessing pipelines were employed.

The algorithms were tested on ten datasets, covering a diverse spectrum of cell types such as dendritic cells, pancreatic cells, retinal cells, and peripheral blood mononuclear cell (PBMCs), with datasets from both human and mouse. The technologies used also span a broad range, including 10x, SMART-seq, Drop-seq, and SMARTer. Dataset details and sources can be found in Additional file 1: Table S1, while the breakdown on cell counts per cell type is in Additional file 2: Table S2. We organized the datasets into five relevant scenarios: identical cell types with different scRNA-seq protocols, non-identical cell types, multiple batches (> 2 batches), big datasets, and simulated data. Scenario 1 consisted of dataset 2 of murine tissues, and dataset 5 of human perpherial blood mononuclear cells (PBMCs). For each dataset, all batches have the same cell types, though the proportion within each batch can vary significantly. The batches were sequenced with different technologies, thus increasing the difficulty of batch correction. Datasets 1, 6, 7, and 10 formed scenario 2, where batches within each dataset have non-identical cell types. The datasets of this scenario encompassed a range of different tissue samples: human dendritic cells, immortalized cell lines, mouse retina cells, and mouse hematopoietic cells. For scenario 3 of multiple batches, we used dataset 4, which contains five batches of human pancreatic cell expression data acquired using four scRNA-seq technologies. The distribution of cell types is also significantly different between batches. Scenario 4 was composed of datasets 8 and 9, where we tested the algorithms on big datasets with more than half a million cells each. In the final scenario, we used the Splatter package [19] to generate simulation datasets with different drop-out rates and unbalanced cell counts across batches. The aim of this scenario was to study the impact of batch correction on downstream differential gene expression analysis. In particular, we were interested in improving the recovery of differentially expressed genes (DEGs) after batch correction. We compared changes in the detected differentially expressed genes in terms of precision and recall with the *F*-score. Coverage of different scenarios by the different datasets can be found in Additional file 3: Table S3.

To evaluate the batch correction results, we employed t-Distributed Stochastic Neighbor Embedding (t-SNE) [20] and Uniform Manifold Approximation and Projection (UMAP) [21] visualizations in conjunction with the *k*-nearest neighbor batch-effect test (kBET) [22], local inverse Simpson’s index (LISI) [13, 23], average silhouette width (ASW) [24], and adjusted rand index (ARI) benchmarking metrics [25]. The UMAP plots are shown here in the main text, while the t-SNE plots are in the Additional file 4: Figure S1–S11. The kBET metric measures batch mixing on the local level using a predetermined number of nearest neighbors, which are selected around each data point by distance, to compute the local batch label distribution. A small proportion of local distributions deviating from the global batch label ratio (i.e., rejection rate) denotes good batch mixing. Here, we computed the kBET rejection rates for local sample sizes at 5%, 10%, 15%, 20%, and 25% to ensure that the assessment was not subject to the choice of sample size. The LISI metric can be used to measure the local cell type distribution (cLISI) or batch distribution (iLISI), based on local neighbors chosen on a preselected perplexity. Here, we used a perplexity of 40. Using the selected neighbors of a cell, the LISI was then computed on the local cell type and batch labels for the cLISI and iLISI indices respectively. For comparison between methods, we took the median value of the scores computed for all cells in the dataset, and scaled such that 0 and 1 denote the worst and best possible scores respectively. We also used the ASW metric to assess batch mixing and preserving cell type purity. For ease of comparison, we plotted the scores as 1-ASW_batch_ and ASW_cell type_, such that a higher value denotes better performance. Similarly, we computed and plotted the ARI scores in the same fashion, 1-ARI_batch_ and ARI_cell type_. To compute the ARI scores, *k*-means clustering was first performed to obtain cluster labels for comparison against batch labels and cell type labels to obtain the ARI_batch_ and ARI_cell type_ scores respectively. All the batch mixing indices were computed for common cell types only, while all cells were used for cell type purity assessment. The computed values of benchmarking metrics can be found in Additional file 5: Table S4, while the statistical tests for significance are in Additional file 6: Table S5. To summarize these metrics, we summed the ranks of each method across all metrics to obtain a rank sum that was used to sort the methods. The rank sums are summarized in Fig. 21a, with details in Additional file 8: Table S7.

### Scenario 1: identical cell types, different technologies

**Fig. 2:**
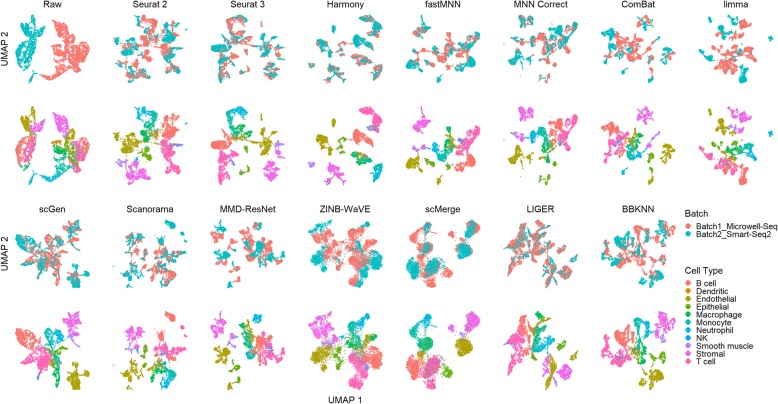
Qualitative evaluation of 14 batch-effect correction methods using UMAP visualization for dataset 2 of mouse cell atlas. The 14 methods are organized into two panels, with the top panel showing UMAP plots of raw data, Seurat 2, Seurat 3, Harmony, fastMNN, MNN Correct, ComBat, and limma outputs, while the bottom panel shows the UMAP plots of scGen, Scanorama, MMD-ResNet, ZINB-WaVE, scMerge, LIGER, and BBKNN outputs. Each panel contains two rows of UMAP plots. In the first row, cells are colored by batch, and in the second by cell type

In this scenario, we tested the methods against datasets that contain the same cell types across all batches. However, different technologies were used to acquire the scRNA-seq data for each batch. For dataset 2, the visualization plots show that Seurat 2, Seurat 3, Harmony, fastMNN, MNN Correct, scGen, Scanorama, scMerge, and LIGER successfully mixed the common cells (Fig. 2). There was minimal cell type mixing, except for the mixing of NK and T cells, which may be attributed to the gene expression similarities of these cell types [26]. ComBat, limma, MMD-ResNet, ZINB-WaVE, and BBKNN were able to bring similar cell types across batches close, but with little to no mixing.

**Fig. 3:**
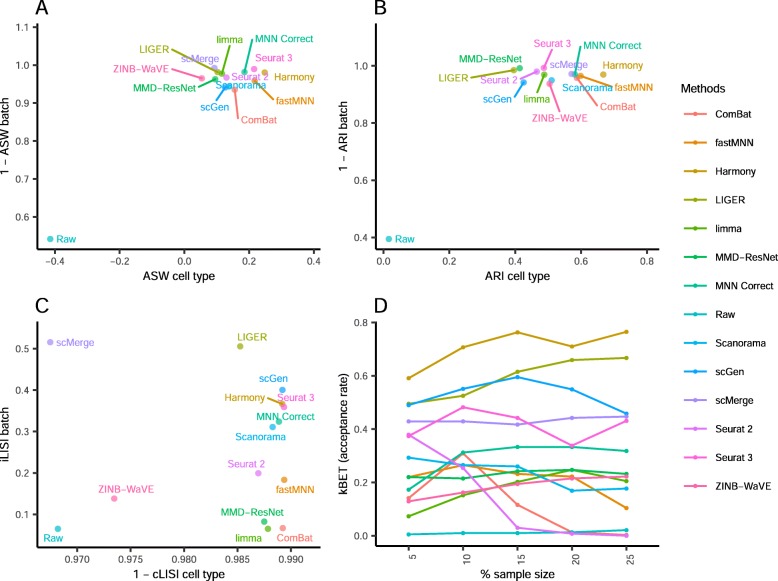
Quantitative evaluation of 14 batch-effect correction methods using the four assessment metrics **a** ASW, **b** ARI, **c** LISI, and **d** kBET on dataset 2 of mouse cell atlas. Methods appearing at the upper right quadrant of the ASW, ARI, and LISI plots are the good performing methods. Methods with higher kBET acceptance rates are the better performing methods

Comparing the iLISI scores, scMerge was the top method for batch mixing, and LIGER was a close second (*p* = 0.015) (Fig. 3). All methods gave good cLISI scores (1-cLISI > 0.96), which is congruent with the visualizations. For kBET, Harmony was top for batch mixing, followed by LIGER and scGen (*p* < 0.001). Using the ASW assessment, Seurat 3 and Harmony were the best methods in balancing between performance in batch and cell type, though all other methods also obtained good scores in batch mixing (1-ASW_batch_ > 0.9). In the ARI scores for batch mixing, all methods scored greater than 0.9, with Harmony obtaining the best ARI cell type score of 0.67 (*p* < 0.001) and an ARI batch score of 0.97. In most metrics, Harmony ranked high, and unsurprisingly, it was also the best method based on the rank sum, with MNN Correct and Seurat 3 tied at second place.

**Fig. 4:**
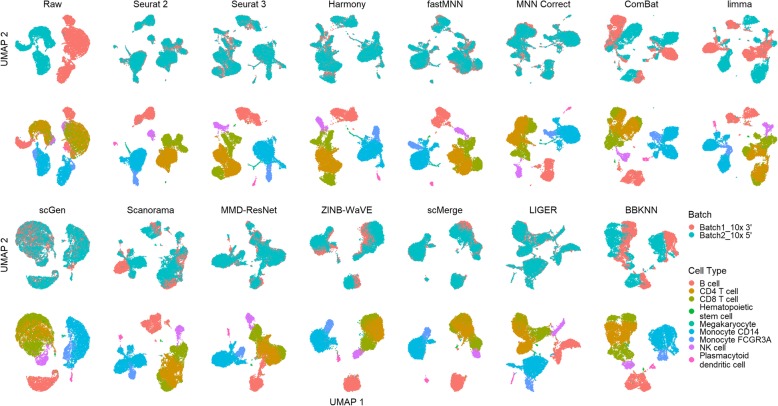
Qualitative evaluation of 14 batch-effect correction methods using UMAP visualization for dataset 5 of human peripheral blood mononuclear cells. The 14 methods are organized into two panels, with the top panel showing UMAP plots of raw data, Seurat 2, Seurat 3, Harmony, fastMNN, MNN Correct, ComBat, and limma outputs, while the bottom panel shows the UMAP plots of scGen, Scanorama, MMD-ResNet, ZINB-WaVE, scMerge, LIGER, and BBKNN outputs. Each panel contains two rows of UMAP plots. In the first row, cells are colored by batch, and in the second by cell type

In dataset 5, there are two pairs of similar cell types, CD4 and CD8, and monocytes CD14 and FCGR3A. None of the methods were able to produce distinct clusters of CD14 and FCGR3A, or CD4 and CD8 in the visualization plots; the FCGR3A cells invariably formed a sub-cluster attached to the CD14 cluster, while CD8 cells formed sub-clusters around CD4 cells (Fig. 4). Seurat 2, Seurat 3, Harmony, fastMNN, and MNN Correct evenly mixed the batches with minimal mixing between CD4 and CD8 sub-clusters. In these cases, some separation of the CD4 and CD8 sub-clusters is visible, especially in the t-SNE plot (Additional file 4: Figure S2). scGen, MMD-ResNet, and LIGER also evenly mixed the batches, but with greater mixing of CD4 and CD8 cells. Scanorama, ZINB-WaVE, and scMerge not only mixed the CD4 and CD8 cells, but also accomplished poorer overall batch mixing. Finally, ComBat, limma, and BBKNN brought the batches close but did not mix them.

**Fig. 5:**
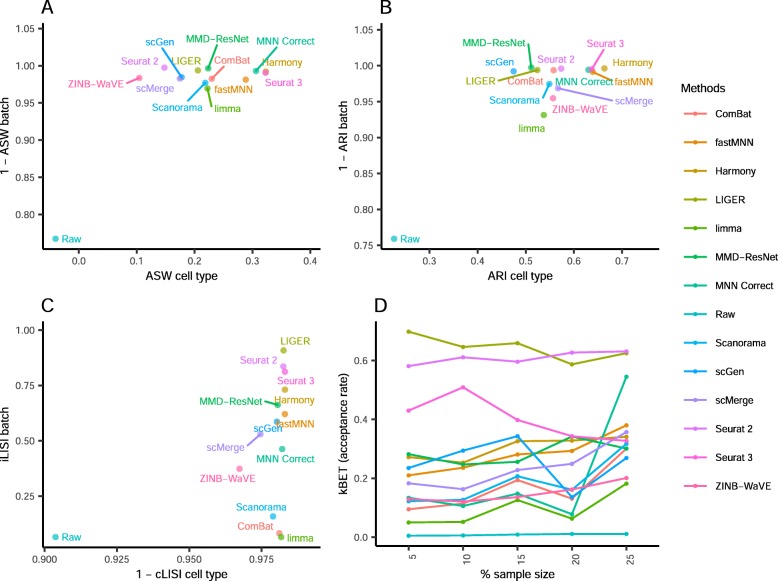
Quantitative evaluation of 14 batch-effect correction methods using the four assessment metrics **a** ASW, **b** ARI, **c** LISI, and **d** kBET on dataset 5 of human peripheral blood mononuclear cells. Methods appearing at the upper right quadrant of the ASW, ARI, and LISI plots are the good performing methods. Methods with higher kBET acceptance rates are the better performing methods

Using the cLISI metric, most methods had good scores for cell type purity of greater than 0.98 (Fig. 5). As the metric only measures local cell purity, the mixing at the edges of cell type-specific sub-clusters were poorly captured by the metric. This resulted in methods with high cLISI scores despite the mixing of CD4 and CD8 cells in the visualization plots. In terms of batch mixing (iLISI), LIGER was top (*p* < 0.001), followed by Seurat 2 and Seurat 3. The computed kBET scores also showed LIGER as the top method with Seurat 2 as a near second, while Seurat 3 was third for batch mixing (*p* < 0.001). In terms of ASW metrics, the batch mixing scores were greater than 0.95 for all methods, while Harmony and Seurat 3 was top in terms of cell type purity (*p* = 0.183), followed by MNN Correct. Similarly with ARI, Harmony, was the best method in terms of cell type purity, followed by fastMNN, Seurat 3, and MNN Correct as next best (*p* < 0.13). These four methods also had high ARI_batch_ scores of greater than 0.97. Using the rank sum, Harmony and Seurat 3 were tied as the best methods overall, with LIGER at the third place.

For both datasets, Harmony was the top method, and Seurat 3 ranked second and third once. Based on these results, both methods are highly recommended for datasets with common cell types. Though LIGER was only ranked third for dataset 5 and tied at fourth place with fastMNN for dataset 2, it was a consistent performer and thus also a competitive method worth considering.

### Scenario 2: non-identical cell types

**Fig. 6:**
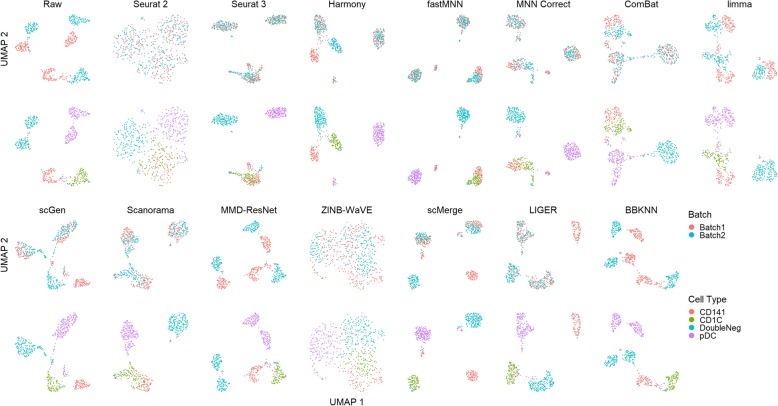
Qualitative evaluation of 14 batch-effect correction methods using UMAP visualization for dataset 1 of human dendritic cells. The 14 methods are organized into two panels, with the top panel showing UMAP plots of raw data, Seurat 2, Seurat 3, Harmony, fastMNN, MNN Correct, ComBat, and limma outputs, while the bottom panel shows the UMAP plots of scGen, Scanorama, MMD-ResNet, ZINB-WaVE, scMerge, LIGER, and BBKNN outputs. Each panel contains two rows of UMAP plots. In the first row, cells are colored by batch, and in the second by cell type

Dataset 1 poses an interesting challenge to batch correction algorithms due to the presence of two highly similar cell types present in dissimilar batches. Examination of the visualization plots shows that most methods were able to mix both batches together (Fig. 6). limma brought cell clusters of both batches close but did not achieve mixing, while MMD-ResNet and BBKNN did not bring any cell clusters of common type closer. scGen, Harmony, LIGER, and scMerge were able to integrate double negative and pDC cells from batches 1 and 2, while keeping CD141 and CD1C cells in separate clusters, with minimal mixing of CD1C, CD141, and double negative cells. The remaining methods produced higher levels of cell type mixing; MNN Correct, fastMNN, Seurat 3, and Seurat 2, and ZINB-WaVE produced single well mixed clusters of CD141 and CD1C cells, while ComBat and Scanorama brought CD1C and CD141 cells close, which would be hard to distinguish as different cell types in the case of unlabeled experimental data. ZINB-WaVE produced a large loose cluster in both tSNE and UMAP. While cell labels show segregation into sub-clusters, these sub-clusters cannot be easily discerned visually without labels.

Most batch correction algorithms require at least one identical cell type to be shared between any pair of data batches, to guide the data alignment. MNN Correct [5], fastMNN [5, 7], Seurat 3 [12], and Scanorama [9] search for MNNs to find the shared populations between different datasets. When there are sub-populations that are not shared between different batches, false matching of the MNNs and incorrect alignment can occur, especially if there are cell types that are highly similar. This appears to be the reason for the clustering of CD1C and CD141 cells together by many methods. The same issue was also been reported with Seurat 3 [12], where the researchers identified a small number of incorrect matches.

**Fig. 7:**
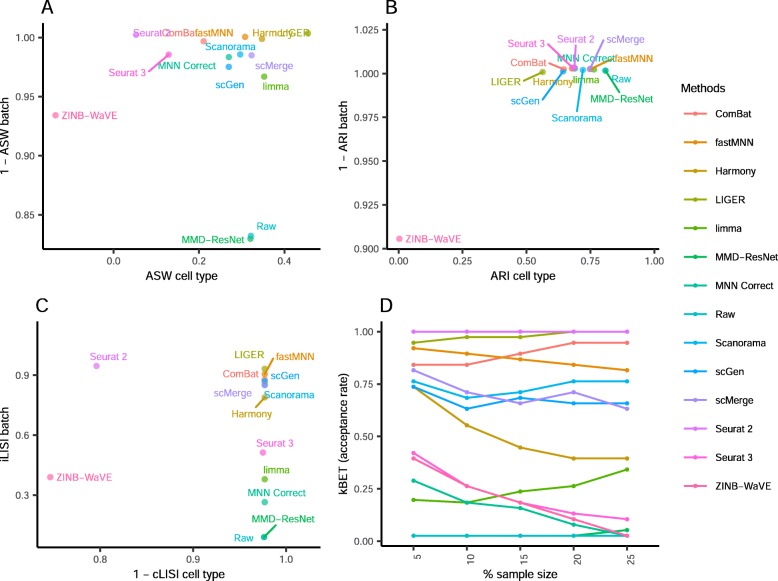
Quantitative evaluation of 14 batch-effect correction methods using the four assessment metrics **a** ASW, **b** ARI, **c** LISI, and **d** kBET on dataset 1 of human dendritic cells. Methods appearing at the upper right quadrant of the ASW, ARI, and LISI plots are the good performing methods. Methods with higher kBET acceptance rates are the better performing methods

In terms of kBET scores, LIGER and Seurat 2 were the best in terms of batch integration for this dataset (Fig. 7). For the iLISI metric, LIGER and Seurat 2 again achieved the highest scores. In terms of cLISI, most methods posted high scores (1-cLISI > 0.96), except for Seurat 2 and ZINB-WaVE. By the ASW metrics, LIGER was the leading method in both cell purity and batch mixing (*p* < 0.001) . Except for ZINB-WaVE and MMD-ResNet, the other methods gave excellent ASW batch integration scores (1-ASW_batch_ > 0.95). For ARI assessment, most methods gave good batch mixing, with the exception of ZINB-WaVE, which was also the worst in terms of cell type purity. Using the rank sum of the metrics, fastMNN emerged as the best method, with LIGER and scMerge ranking second and third respectively.

**Fig. 8:**
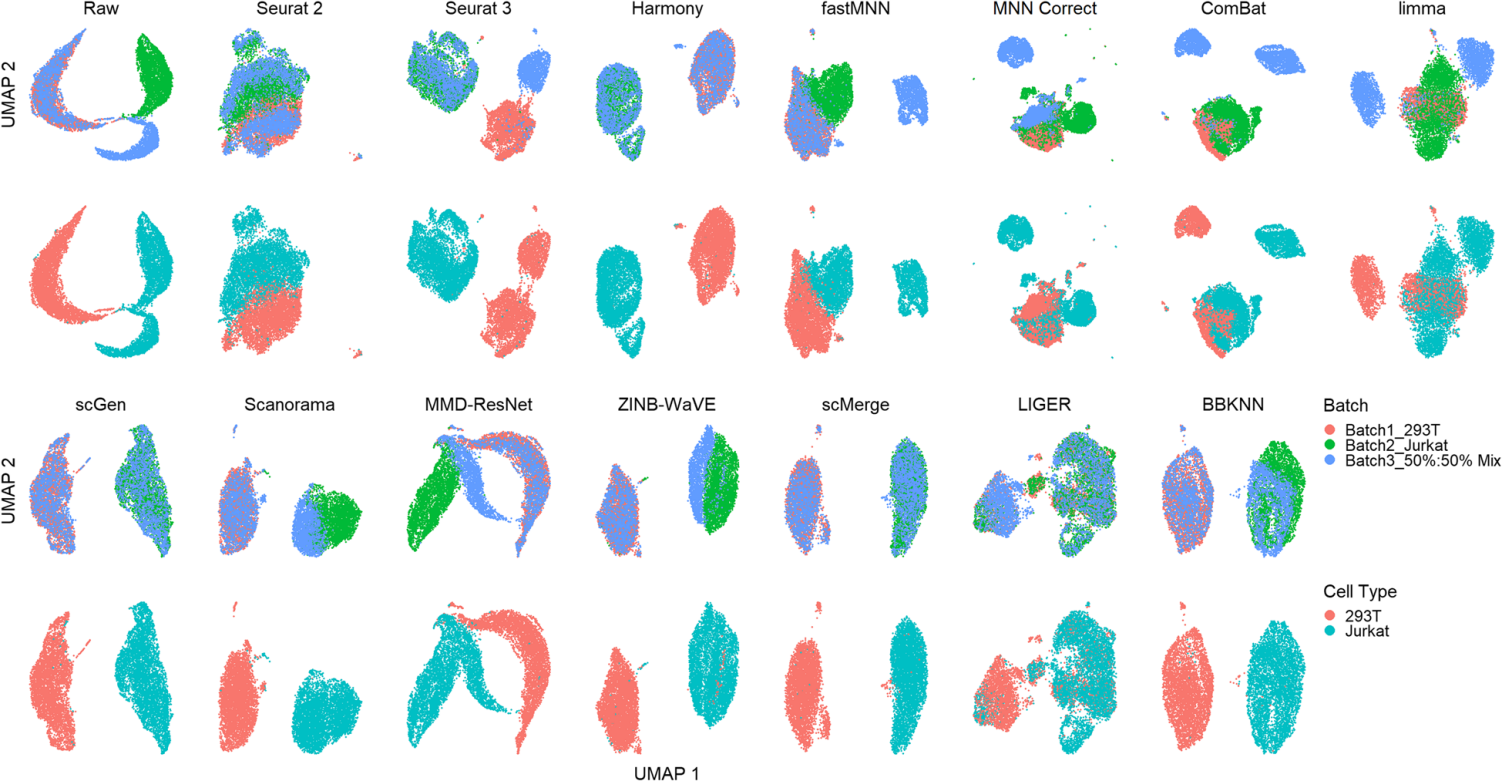
Qualitative evaluation of 14 batch-effect correction methods using UMAP visualization for dataset 6 of cell lines. The 14 methods are organized into two panels, with the top panel showing UMAP plots of raw data, Seurat 2, Seurat 3, Harmony, fastMNN, MNN Correct, ComBat, and limma outputs, while the bottom panel shows the UMAP plots of scGen, Scanorama, MMD-ResNet, ZINB-WaVE, scMerge, LIGER, and BBKNN outputs. Each panel contains two rows of UMAP plots. In the first row, cells are colored by batch, and in the second by cell type

Dataset 6 contains only two cell types, with two out of the three batches containing only one cell type that is also only shared with the third batch. The t-SNE and UMAP plots show that scGen, scMerge, and BBKNN were able to produce two large cell type-specific clusters (293T and Jurkat) that were well mixed with cells from their respective batches (Fig. 8). Harmony also mixed the batches well, but with the Jurkat cells divided into two clusters. LIGER also produced two batch mixed clusters, but with some cell type mixing. In Seurat 2’s output, the batches were mixed, but the 293T and Jurkat cell clusters were too closely positioned to be easily separated visually. fastMNN, Scanorama, ZINB-WaVE, and MMD-ResNet batch mixed the 293T cells but not the Jurkat cells, while Seurat 3 only mixed the Jurkat cells. Finally, MNN Correct, ComBat, and limma incorrectly mixed the Jurkat and 293T cells from different batches.

**Fig. 9:**
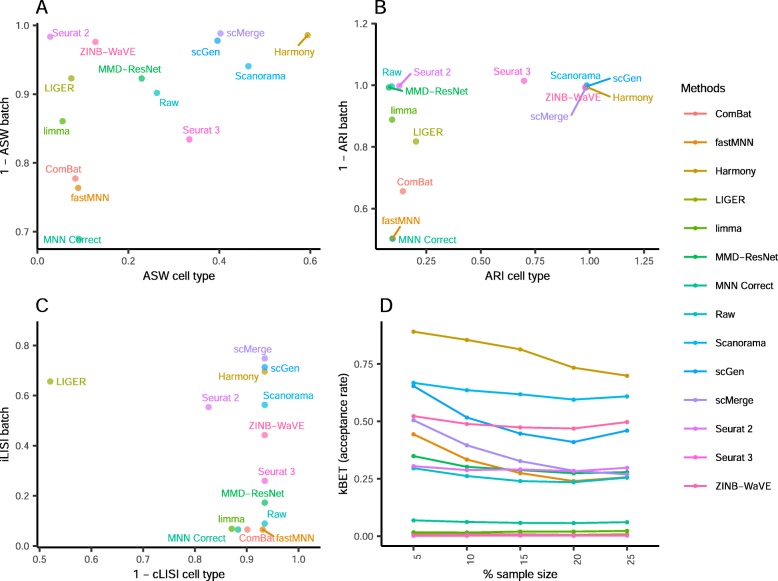
Quantitative evaluation of 14 batch-effect correction methods using the four assessment metrics **a** ASW, **b** ARI, **c** LISI, and **d** kBET on dataset 6 of cell lines. Methods appearing at the upper right quadrant of the ASW, ARI, and LISI plots are the good performing methods. Methods with higher kBET acceptance rates are the better performing methods

The LISI metrics also indicate that Harmony, scMerge, and scGen were the best methods for this dataset in terms of batch integration and cell type purity (Fig. 9). Similarly, Harmony was top ranked in kBET, followed by scGen and Scanorama, despite the relatively poor batch mixing of Jurkat cells by Scanorama in the visualizations. Using the ASW metrics, Harmony was the leading method, followed by scMerge, scGen, and Scanorama, with Scanorama showing lower batch integration but higher cell type purity. With the ARI metrics, scGen, scMerge, ZINB-WaVE, Harmony, and Scanorama were the top methods. Based on the rank sum of the assessment metrics, Harmony was the top method, followed by Scanorama and scGen. These results are quite consistent across all metrics, which gives confidence on our assessment of the methods. Due to the BBKNN’s output being a graph, assessment metrics could not be computed. However, based on the UMAP visualization, we consider BBKNN to be a competitive method.

**Fig. 10:**
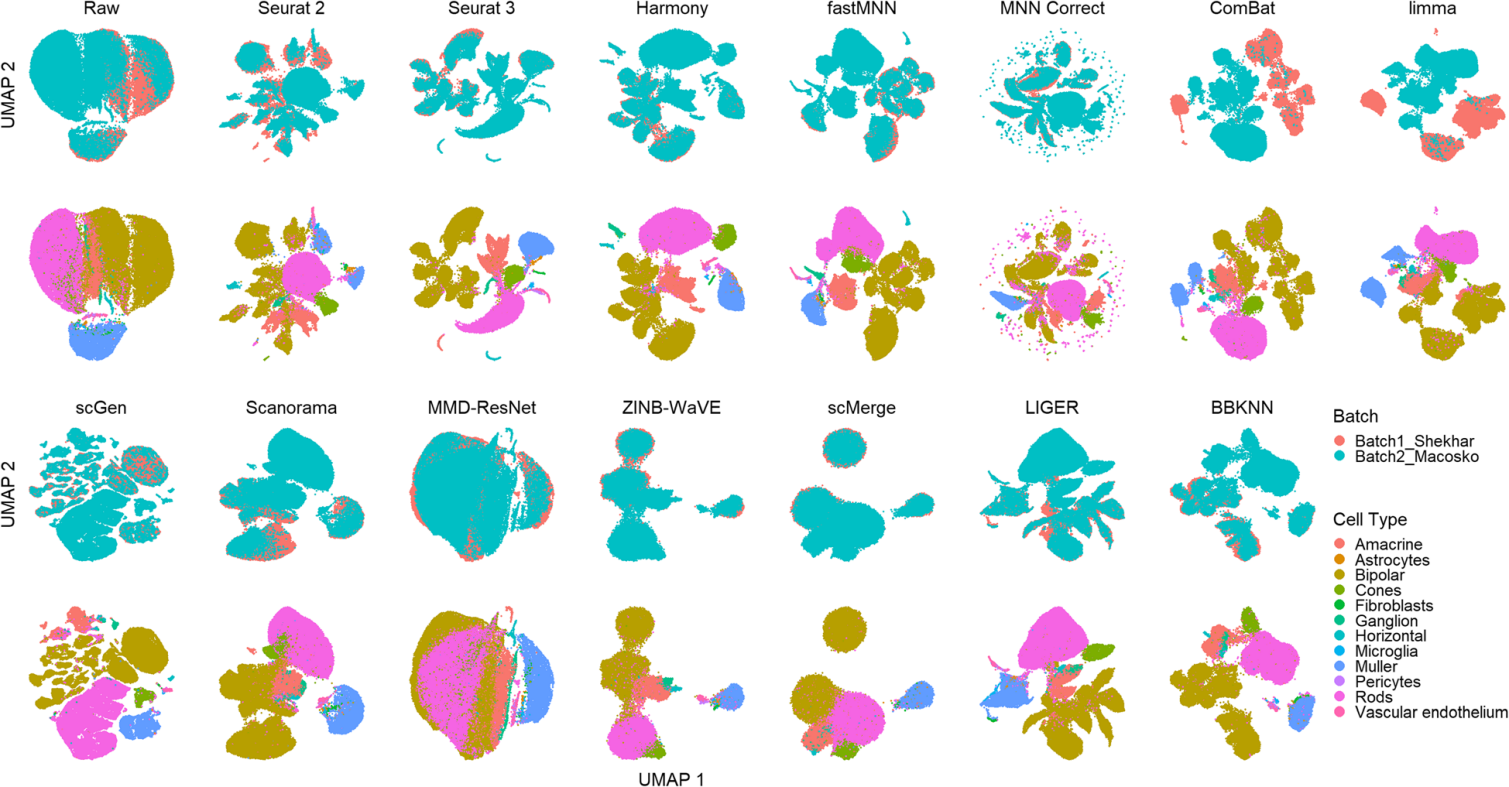
Qualitative evaluation of 14 batch-effect correction methods using UMAP visualization for dataset 7 of mouse retinal cells. The 14 methods are organized into two panels, with the top panel showing UMAP plots of raw data, Seurat 2, Seurat 3, Harmony, fastMNN, MNN Correct, ComBat, and limma outputs, while the bottom panel shows the UMAP plots of scGen, Scanorama, MMD-ResNet, ZINB-WaVE, scMerge, LIGER, and BBKNN outputs. Each panel contains two rows of UMAP plots. In the first row, cells are colored by batch, and in the second by cell type

For dataset 7 (Fig. 10), the cell counts across cell types are highly uneven with batch 1 predominantly made up of bipolar cells (88%), and smaller numbers of amacrine, Muller, cone, and rod cells. On the other hand, batch 2 contains more cell types with 66% being rod cells (see Additional file 2: Table S2) and cell types such as ganglion, vascular endothelium, and horizontal that are not found in batch 1. From the visualization plots, the batch effect does not appear to be significant. In particular, the Muller and bipolar cells already show substantial mixing. Surprisingly, ComBat and limma separated the shared cell types to form batch and cell type separated clusters instead. ZINB-WaVE, scMerge, and MMD-ResNet clustered most of the cells into a single large cluster, albeit with intra-cluster segregation among the cell types. The remaining methods were largely able to mix the common cells while maintaining cell type purity among clusters, though Seurat 2 separated the Muller cells from both batches.

**Fig. 11:**
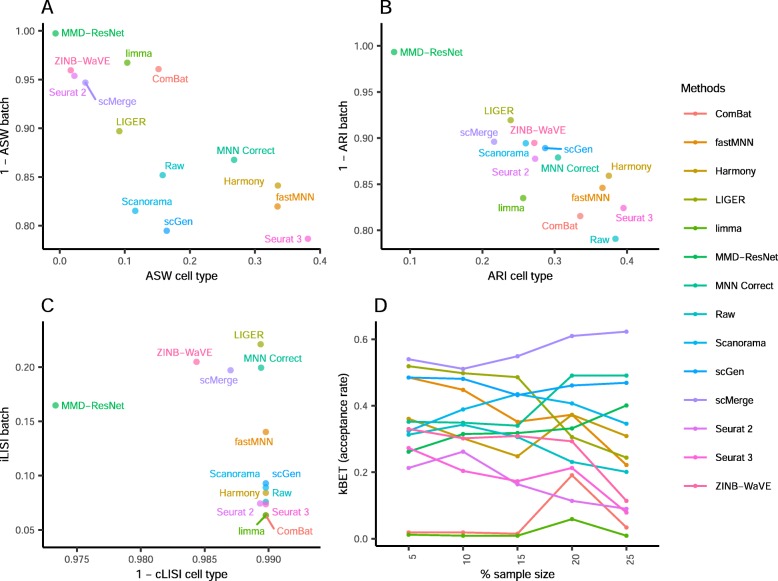
Quantitative evaluation of 14 batch-effect correction methods using the four assessment metrics **a** ASW, **b** ARI, **c** LISI, and **d** kBET on dataset 7 of mouse retinal cells. Methods appearing at the upper right quadrant of the ASW, ARI, and LISI plots are the good performing methods. Methods with higher kBET acceptance rates are the better performing methods

Using the LISI metrics, we assessed batch integration and cell type purity where LIGER was top for batch integration (*p* < 0.001) and the best method overall (Fig. 11). On the other hand, the computed kBET metric shows scMerge as the best for batch integration (*p* < 0.001). Meanwhile, the ASW metrics show no clearly superior method, with methods showing tradeoff between scores in batch mixing and cell type purity. The metric also shows a high batch mixing score for ComBat, despite the lack of batch mixing in the visualization plots. In the case of ARI, trade-offs between batch and cell type metrics can be seen among the methods, without a clearly superior method. Using the rank sum to combine the assessment metric results, LIGER was the top performing method with MNN Correct second and scMerge third. For this dataset, it is difficult to determine a clearly superior method by visual inspection or evaluation metrics. While LIGER can be concluded to be the best based on the metrics, the visualizations of other methods such as Harmony, Seurat 3, and scGen suggest that these methods were also able to perform batch integration and preserve cell type purity, despite their fairly low rankings in the rank sum.

**Fig. 12:**
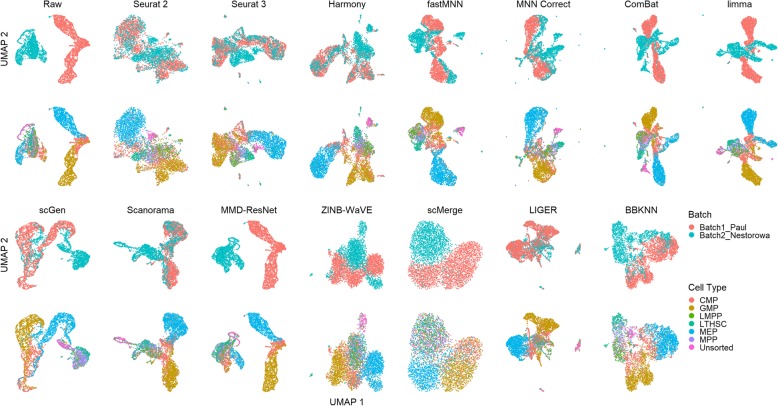
Qualitative evaluation of 14 batch-effect correction methods using UMAP visualization for dataset 10 of mouse hematopoietic stem and progenitor cells. The 14 methods are organized into two panels, with the top panel showing UMAP plots of raw data, Seurat 2, Seurat 3, Harmony, fastMNN, MNN Correct, ComBat, and limma outputs, while the bottom panel shows the UMAP plots of scGen, Scanorama, MMD-ResNet, ZINB-WaVE, scMerge, LIGER, and BBKNN outputs. Each panel contains two rows of UMAP plots. In the first row, cells are colored by batch, and in the second by cell type

In dataset 10, batch 1 contains only CMP, GMP, and MEP cells, while batch 2 contains all cell types. Based on the cell type information, we can expect the clustering of cells to reflect their developmental lineage, with GMP and MEP forming their respective clusters that are also close to CMP cells [5]. In the visualization plots, Seurat 2, Seurat 3, Harmony, Scanorama, and LIGER, were able to batch mix the GMP and MEP cells, with the expected mixing of other cell types (Fig. 12). fastMNN, MNN Correct, scGen, and BBKNN brought the GMP and MEP cells close with minimal batch mixing. ZINB-WaVE and scMerge moved the cells closer to form a big loose cluster, while ComBat and limma moved the batches closer but no batch mixing occurred. Finally, MMD-ResNet made almost no impact on the distribution of cells.

**Fig. 13:**
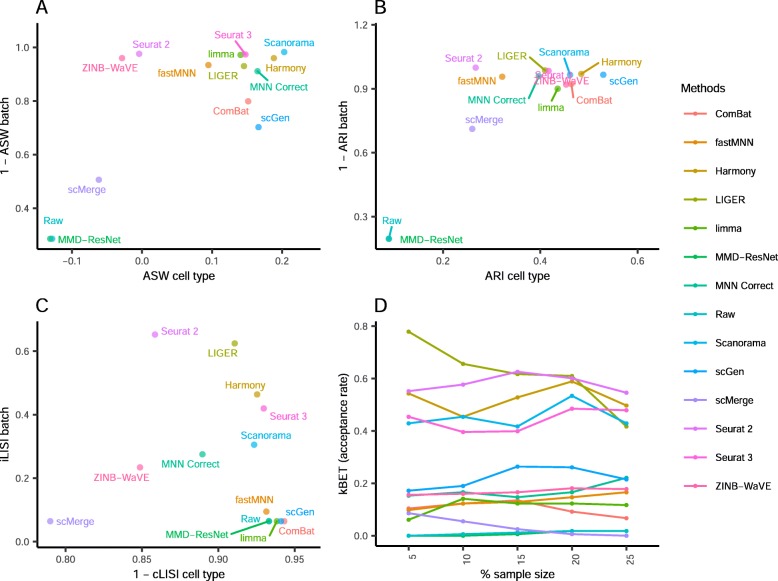
Quantitative evaluation of 14 batch-effect correction methods using the four assessment metrics **a** ASW, **b** ARI, **c** LISI, and **d** kBET on dataset 10 of mouse hematopoietic stem and progenitor cells. Methods appearing at the upper right quadrant of the ASW, ARI, and LISI plots are the good performing methods. Methods with higher kBET acceptance rates are the better performing methods

The iLISI metric computed shows that Seurat 2 was the best for batch mixing, followed closely by LIGER (*p* = 0.057), though LIGER's output is superior in cell type purity (cLISI) with *p* value < 0.001 (Fig. 13). Harmony and Seurat 3 ranked third and fourth respectively for iLISI but with increasingly better cLISI scores. A similar trend can be seen in the kBET results with LIGER as the top result, followed by Seurat 2 and Harmony (*p* < 0.001). Based on the ASW metrics, Scanorama was the top method for both batch mixing and cell type purity (*p* < 0.001). The ARI scores gave very different results with scGen as the top method for cell type purity (*p* < 0.001) while having a batch mixing score comparable to other methods with high ARI_batch_ scores (> 0.9). Using the rank sum to summarize the evaluations, Harmony, Scanorama, and LIGER were the top methods for this dataset. While Harmony did not top any metric, it was ranked second in three metrics (ASW, ARI, and LISI) and third for kBET; the results highlight Harmony's efficacy on this dataset.

In this scenario, we tested the batch correction methods on four diverse datasets. While no method was the best for all datasets, LIGER was one of the top three methods for three datasets (1, 7, 10), while scMerge was ranked third for datasets 1, 6, and 7. Harmony ranked first for datasets 6 and 10, while Scanorama ranked second for datasets 6 and 10. Based on these results, LIGER was the leading method in this scenario.

### Scenario 3: multiple batches

**Fig. 14:**
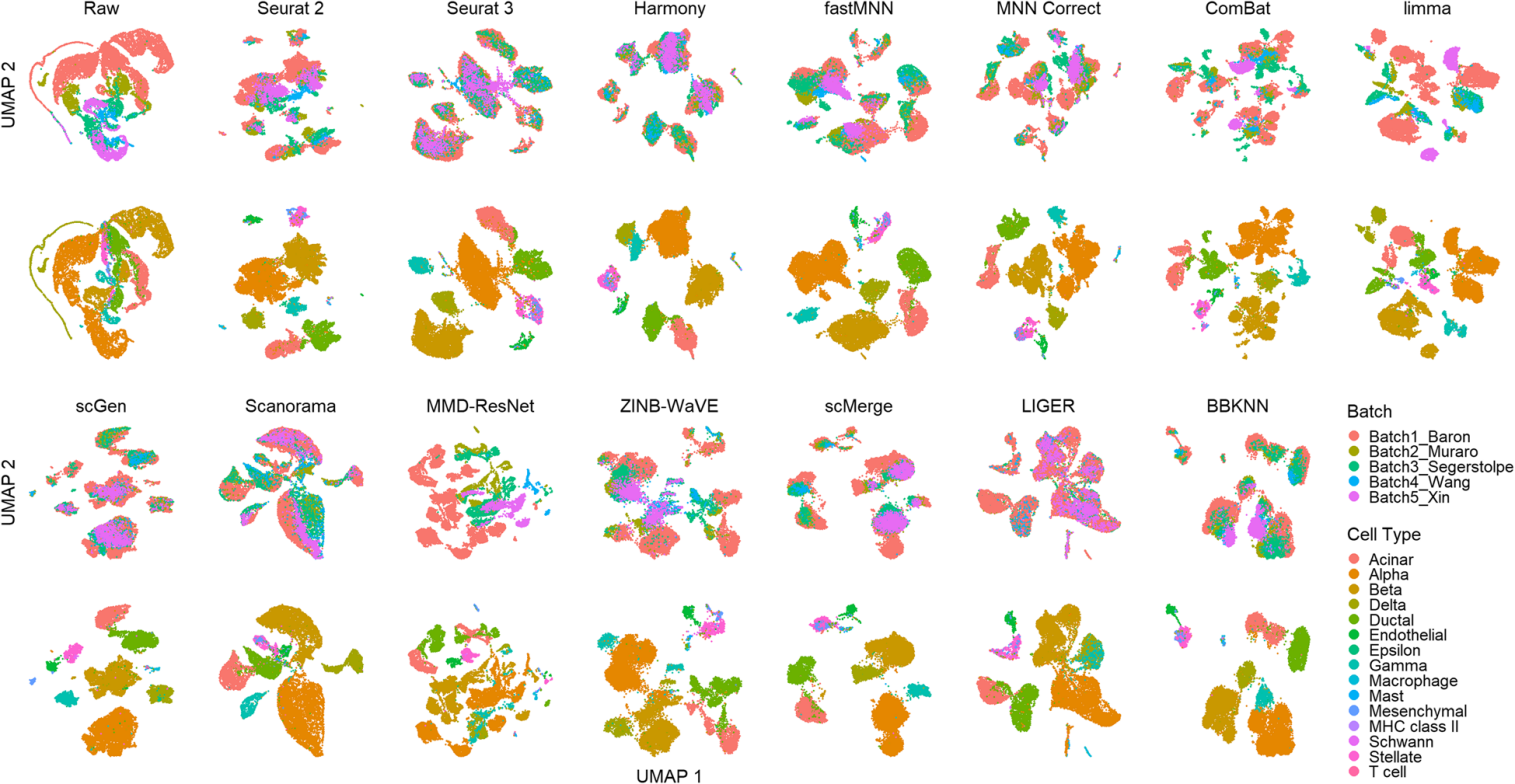
Qualitative evaluation of 14 batch-effect correction methods using UMAP visualization for dataset 4 of human pancreatic cells. The 14 methods are organized into two panels, with the top panel showing UMAP plots of raw data, Seurat 2, Seurat 3, Harmony, fastMNN, MNN Correct, ComBat, and limma outputs, while the bottom panel shows the UMAP plots of scGen, Scanorama, MMD-ResNet, ZINB-WaVE, scMerge, LIGER, and BBKNN outputs. Each panel contains two rows of UMAP plots. In the first row, cells are colored by batch, and in the second by cell type

This scenario tested batch correction abilities with multiple batches. Dataset 4 consists of five batches of human pancreatic cells sequenced with four technologies. The t-SNE and UMAP plots show that Seurat 3, Harmony, scGen, and LIGER produced clusters that evenly mixed with cells from different batches (Fig. 14). The batch mixing was less even for Seurat 2, fastMNN, Scanorama, ZINB-WaVE, scMerge, and BBKNN. The above methods also mixed the stellate and mesenchymal cells to varying extents except for scGen, which can be attributed to the supervised nature of the method. Delta and gamma cells were also clustered close by LIGER and Harmony, though better separation can be seen in Harmony’s t-SNE plot (Additional file 4: Figure S7). MNN Correct, ComBat, limma, and MMD-ResNet brought cell-specific clusters from different batches close, but without significant batch mixing. The cell types were also broken up into multiple smaller clusters by these methods.

**Fig. 15:**
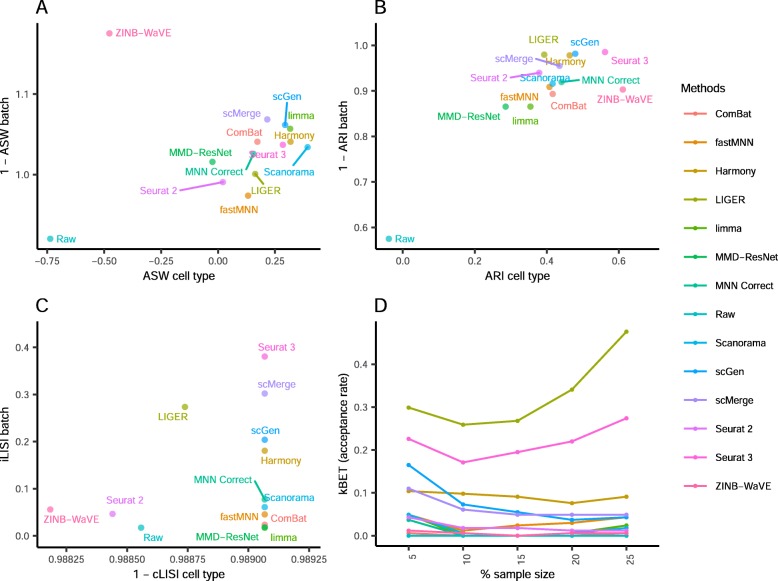
Quantitative evaluation of 14 batch-effect correction methods using the four assessment metrics **a** ASW, **b** ARI, **c** LISI, and **d** kBET on dataset 4 of human pancreatic cells. Methods appearing at the upper right quadrant of the ASW, ARI, and LISI plots are the good performing methods. Methods with higher kBET acceptance rates are the better performing methods

From the LISI metrics, the cell type purity of the method outputs was high (> 0.98), while Seurat 3 was also top in batch integration (*p* < 0.001) (Fig. 15). Seurat 3 was also second in batch mixing by the kBET metric, while LIGER was top (*p* < 0.001). Assessment by ASW showed that ZINB-WaVE ranked top for batch integration (*p* < 0.001), though the earlier visual analysis shows that it did not mix the batches well. The other methods show poorer ASW batch scores but higher ASW cell type scores with Scanorama as the best (*p* < 0.001). All methods received high ARI batch integration scores (> 0.85), despite the lack of batch mixing by methods such as ComBat and limma. However, the leading methods (score > 0.99) of Seurat 3, Harmony, scGen, and LIGER were largely similar to the results obtained with iLISI. For ARI cell type assessment, ZINB-WaVE received the highest score, though not significantly different (*p* value > 0.05) compared to other methods except MMD-ResNet and limma. Using the rank sum to summarize the metrics, Seurat 3 was the leading method, followed by scGen and scMerge. Returning to the analysis with t-SNE and UMAP visualizations, we can concur with the rankings. While dataset 6 was analyzed in scenario 2, the dataset also contains more than two batches and therefore relevant to this scenario. For dataset 6, the top three methods were Harmony, Scanorama, and scGen, with scMerge ranked fourth. Taking the ranking results of both datasets into consideration, for datasets with labeled cell types, scGen is recommended based on its performance. For unlabeled data, Seurat 3 and Harmony are the recommended choices.

### Scenario 4: big data

In this scenario, we tested the methods on two large datasets with more than 100,000 cells each. BBKNN, ComBat, Harmony, LIGER, limma, MMD-ResNet, Scanorama, scGen, Seurat 3, and ZINB-WaVE were able to complete runs on the full datasets. The remaining four methods were unable to complete the batch correction runs due to memory or runtime requirements. FastMNN, scMerge, and Seurat 2 required more than 2.27 TB of memory and caused excessive disk thrashing from virtual memory usage, while MNN Correct was able to run but took too long (> 48 h). The metrics and visualizations shown were computed on the 10% downsampled data; however, our analysis here focuses on the methods that could complete running on the large datasets.

**Fig. 16:**
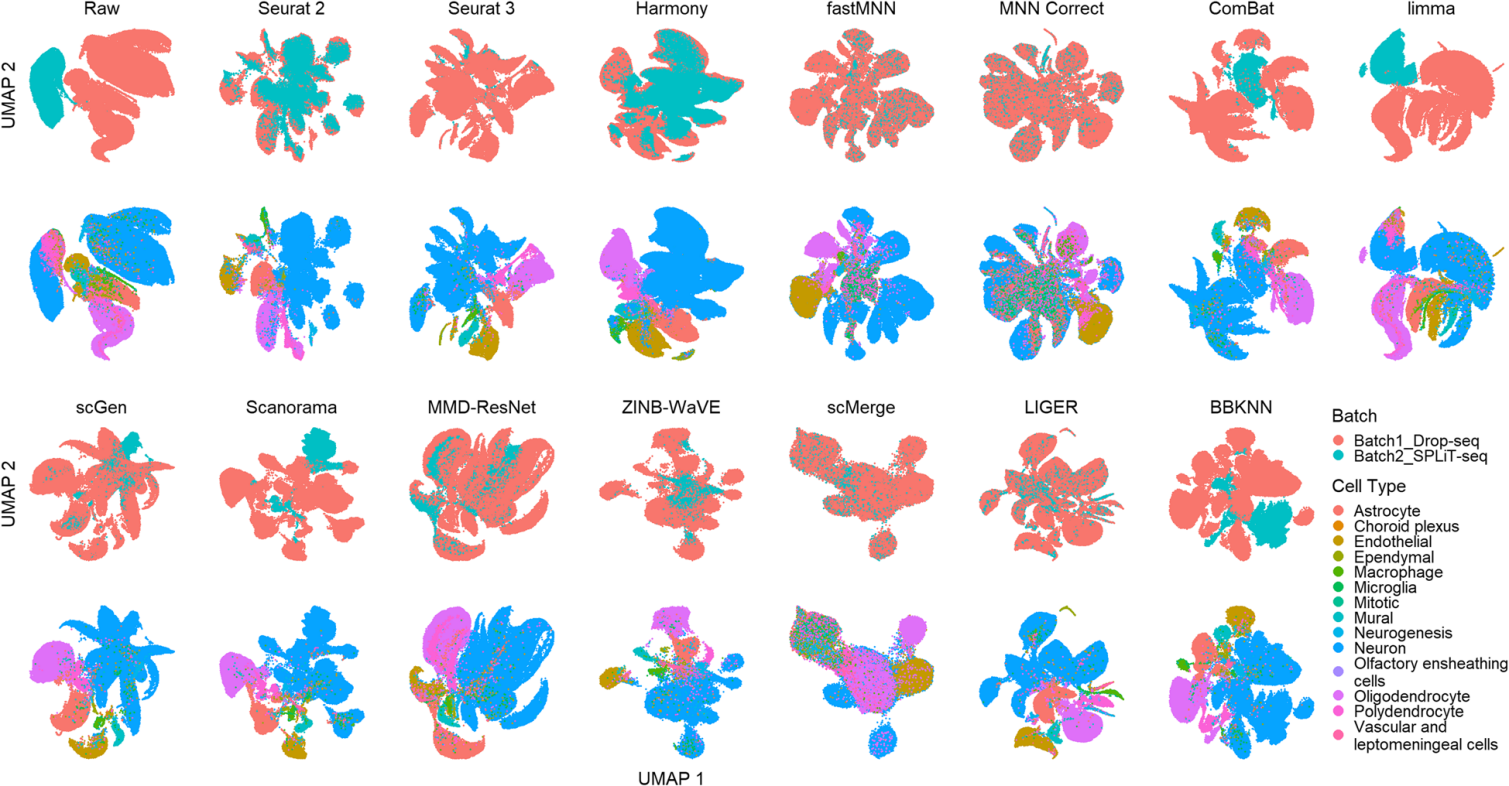
Qualitative evaluation of 14 batch-effect correction methods using UMAP visualization for dataset 8 of mouse brain. The 14 methods are organized into two panels, with the top panel showing UMAP plots of raw data, Seurat 2, Seurat 3, Harmony, fastMNN, MNN Correct, ComBat, and limma outputs, while the bottom panel shows the UMAP plots of scGen, Scanorama, MMD-ResNet, ZINB-WaVE, scMerge, LIGER, and BBKNN outputs. Each panel contains two rows of UMAP plots. In the first row, cells are colored by batch, and in the second by cell type

**Fig. 17:**
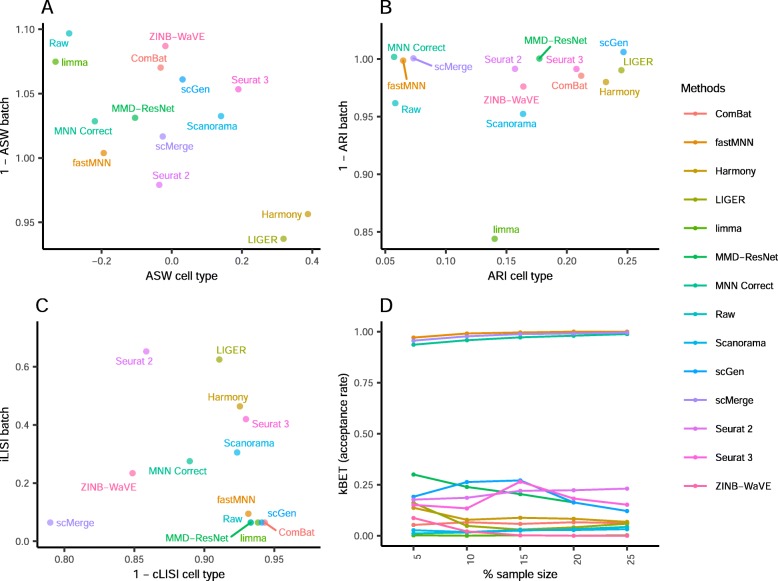
Quantitative evaluation of 14 batch-effect correction methods using the four assessment metrics **a** ASW, **b** ARI, **c** LISI, and **d** kBET on dataset 8 of mouse brain. Methods appearing at the upper right quadrant of the ASW, ARI, and LISI plots are the good performing methods. Methods with higher kBET acceptance rates are the better performing methods

Dataset 8 consists of two batches of murine brain data acquired using different technologies (Fig. 16). The cell numbers are unevenly distributed across cell types, and the bulk of the cells in batch 2 consist of astrocytes, neurons, oligodendrocytes, and polydendrocytes. Only LIGER appears to have maintained relatively good cell type separation while achieving batch mixing. Seurat 3, Harmony, ZINB-WaVE, scGen, and MMD-ResNet produced comparatively less even batch mixing. ComBat, limma, Scanorama, and BBKNN fared even poorer with little to no batch mixing. Employing the LISI metrics, all methods maintained high local cell type purity with good cLISI scores (1-cLISI > 0.8) (Fig. 17). Among the methods that could complete running on the large datasets, LIGER and Seurat 2 achieved the highest iLISI scores among the methods (*p* value = 0.057), followed by Harmony and Seurat 3 (*p* < 0.001). Within the LISI metrics, we can see some trade off between batch integration and cell type purity among LIGER, Harmony, and Seurat 3. Surprisingly, the kBET metric shows very different results, with none of these methods achieving a good score. Instead, fastMNN, scMerge, and MNN Correct were the top three methods by kBET. The ASW metrics also paint a substantially different picture with ZINB-WaVE being the best in batch mixing, though most methods showed high batch mixing scores (1-ASW_batch_ > 0.93) as well, while Harmony produced the highest cell type purity (*p* < 0.001). The ARI results also differ, most methods were also able to produce high batch mixing scores greater than 0.95 (except for limma). Overall, with scGen being the best method, being top for batch mixing (*p* < 0.001), and tied with LIGER for cell type purity (*p* = 0.34). In terms of ARI cell type purity, scGen and LIGER were followed by Harmony. Combining the ranking across all metrics with rank sum, Seurat 3 ranked first, followed by scGen and Seurat 2.

**Fig. 18:**
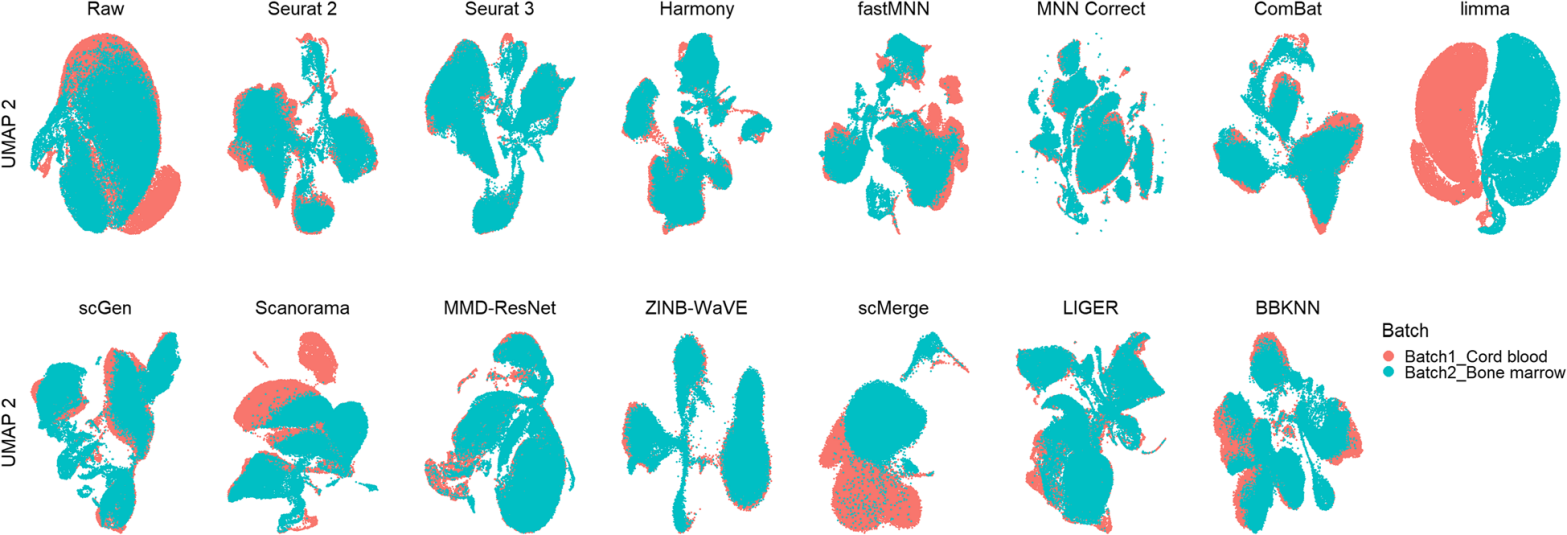
Qualitative evaluation of 14 batch-effect correction methods using UMAP visualization for dataset 9 of human cell atlas. The 14 methods are organized into two panels, with the top panel showing UMAP plots of raw data, Seurat 2, Seurat 3, Harmony, fastMNN, MNN Correct, ComBat, and limma outputs, while the bottom panel shows the UMAP plots of scGen, Scanorama, MMD-ResNet, ZINB-WaVE, scMerge, LIGER, and BBKNN outputs. Cells are colored by batch

**Fig. 19:**
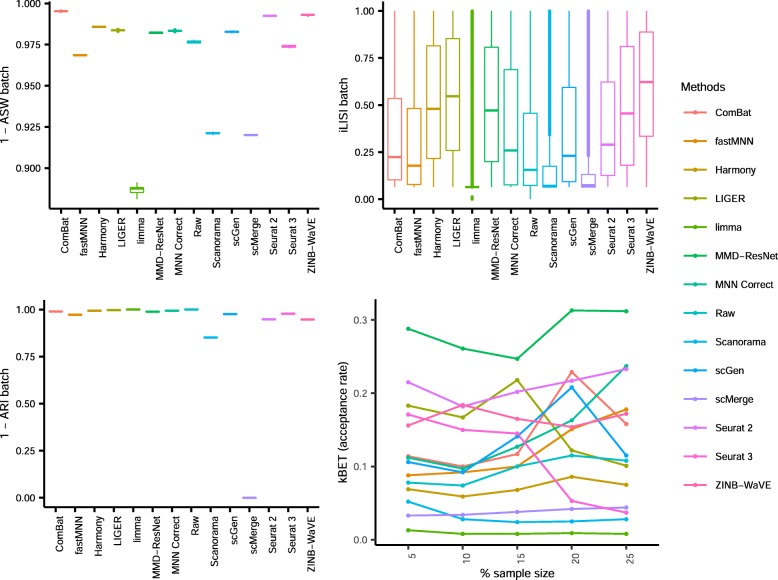
Quantitative evaluation of 14 batch-effect correction methods using the four assessment metrics **a** ASW, **b** ARI, **c** LISI, and **d** kBET on dataset 9 of human cell atlas. Methods appearing at the upper right quadrant of the ASW, ARI, and LISI plots are the good performing methods. Methods with higher kBET acceptance rates are the better performing methods

Dataset 9 is composed of two data batches, each derived from different tissue. Due to the lack of available cell type information, we could only assess batch mixing capabilities. From the plots, the raw data shows substantial mixing. Visually, most methods were able to evenly mix the batches, except for scMerge, limma, and Scanorama (Fig. 18). ComBat improved the mixing slightly, but limma separated the batches instead. Harmony, LIGER, MMD-ResNet, Seurat 3, and ZINB-WaVE produced iLISI scores above 0.45 (*p* < 0.05), significantly better than the other methods (Fig. 19). In terms of the kBET scores, MMD-ResNet was consistently better than all other methods at all sample sizes (*p* < 0.001). Batch mixing measured by ASW shows that most methods achieved mixing better than the raw data, except for limma, scMerge, fastMNN, Scanorama, and Seurat 3. The ASW_batch_ score for Seurat 3 is surprisingly poorer than competing methods, given its visualization plots and rankings in the other rankings. In the ARI batch mixing assessment, most methods obtained high scores (> 0.9). Surprisingly, limma had a high score (> 0.99), which is comparable to other leading methods while its performance measured by other metrics was poor. Summarizing the various metrics, the computed rank sum showed LIGER as the top method, with ZINB-WaVE ranked second and MMD-ResNet third.

Among the methods that ranked in the top three, LIGER and Seurat 3 ranked top for one dataset each. Visual examination of the plots also supports this result, thus making both methods recommended for large datasets.

### Scenario 5: simulation

**Fig. 20:**
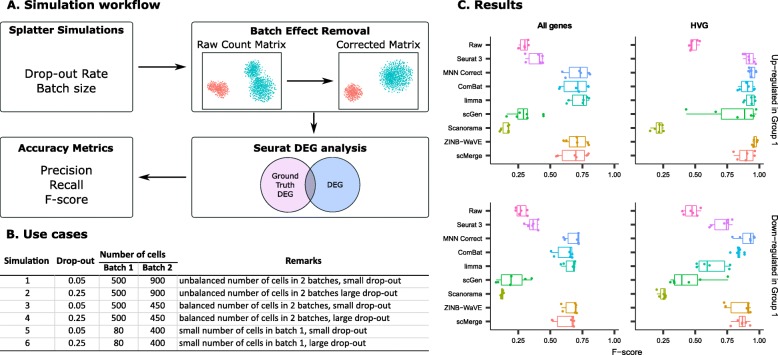
Evaluation of eight batch-effect correction methods using simulated datasets and differential gene expression analysis. **a** Evaluation workflow: six sets of simulation data with predefined batch effect and differential gene expression profiles were generated using the Splatter package with varied parameters. The eight methods that return corrected expression matrices were applied to the simulated data, and the batch-corrected output were subsequently subjected to differential gene expression analysis with the Seurat package. Differentially expressed genes (DEGs) identified from the batch-corrected matrices were compared to the ground truth DEGs, and accuracy metrics including precision, recall, and *F*-score were calculated. **b** Description of the six simulated datasets. Different combinations of parameters were used to cover different scenarios of cell population sizes and drop-out rates. **c** *F*-score boxplot for the eight methods using all genes or HVGs

One of the uses of batch integration is to obtain a corrected gene expression matrix for downstream analysis. This is illustrated by several recent publications that used batch-effect-corrected matrices for analyses, such as pseudotime trajectory analysis [9, 18] and DEG analysis [5, 12]. However, these only compared a small number of batch-effect removal methods and test cases. Herein, we performed a comprehensive evaluation of eight methods that return batch-corrected expression matrices. We evaluated the methods in terms of detecting differential gene expression, using the DEG analysis workflow shown in Fig. 20a. Six different use cases were designed using the Splatter simulation package [19] in the R language environment to cover different scenarios of cell population sizes and drop-out rates, including small and large drop-out rates, balanced and unbalanced cell counts in two batches, and also the case of small cell numbers in one batch. The simulation details are shown in Fig. 20b. The advantage of employing simulation data generated with Splatter is that the true number of DEGs is known, thus enabling us to study the impact of batch correction on the fraction of true DEGs recovered.

For this study, we only considered the methods that produce a batch-effect-corrected gene expression matrix: Seurat 3, MNN Correct, ComBat, limma, scGen, Scanorama, ZINB-WaVE, and scMerge. The simulated gene expression matrices with batch effects were used as input to the batch-effect correction methods. We tested the batch-effect removal methods with two cases for each expression matrix, using all genes, and only highly variable genes (HVGs). DEG detection was performed on the batch-corrected matrices. We first detected DEGs between cell type “Group 1” versus cell type “Group 2” and computed the *F*-score of upregulated and downregulated genes in “Group 1.” We then calculated the accuracy metrics of true positive (TP), false positive (FP), false negative (FN), and *F*-score to examine the accuracy of DEGs obtained from each corrected matrix, as compared to the true DEGs. The *F*-scores are summarized in Fig. 20c.

As seen in Fig. 20c, the median *F*-scores of MNN Correct, ComBat, and limma were 0.73, 0.71, and 0.76 for upregulated genes in the case of all genes, and 0.94, 0.91, and 0.94 for upregulated genes in the case of HVGs. Statistically, there was no performance difference between these methods (Wilcoxon *p* value > 0.05, Additional file 7: Table S6B). We also see a similar trend in the *F*-score results for downregulated genes in the case of all genes. However, if we considered only downregulated HVGs, limma was not able to remove batch effects and resulted in lower *F*-score than MNN Correct and ComBat with statistical significance (Wilcoxon *p* value < 0.01, Fig. 20c, Additional file 7: Table S6E). The median *F*-scores of ZINB-WaVE and scMerge were 0.71 and 0.70 for upregulated genes (all genes), and 0.96 and 0.9 for upregulated genes (HVGs), with no statistically significant differences (Wilcoxon *p* value > 0.05, Additional file 7: Table S6B). Finally, in the case of Seurat 3, scGen, and Scanorama, the median *F*-scores were 0.42, 0.29, and 0.17 in upregulated genes (all genes), and 0.92, 0.88, and 0.22 in upregulated genes (HVGs). Statistical tests showed no significant difference between Seurat 3 and scGen, but there was a significant difference between the results of Seurat 3 and Scanorama (Wilcoxon *p* value < 0.05), and between scGen and Scanorama (Wilcoxon *p* value < 0.01). In particular, Scanorama’s *F*-score was lower than the raw, implying that the method removed most of the cell type variation between “Group 1” and “Group 2.” This is a crucial point, as the goal of batch correction is to remove variations due to data acquisition under different conditions and technologies, while preserving variations of biological origin. Therefore, the *F*-score results of DEG analysis applied on corrected results should have higher accuracy compared to the raw data. For most methods, the *F*-scores of the batch-corrected data were higher than those of the raw data, except for scGen and Scanorama. This shows that the batch-effect removal methods were effective in removing the technical variance between data batches of our simulated data. Based on the *F*-scores, MNN Correct, ZINB-WaVE, ComBat, and scMerge were the top performing methods (Fig. 20c, Additional file 7: Table S6).

The proportion of correctly detected upregulated genes was higher for the HVG set than for the all genes set. This is unsurprising, since HVGs are more likely to retain their cell type variations than genes with lower variability. Therefore, applying batch-effect correction on selected HVGs will give better precision, but restricts the study. If it is necessary to perform batch correction on the full gene dataset, then one should be careful of erroneous conclusions that may arise from the false positives and negatives.

Next, we examined the accuracy metrics of the methods (Additional file 7: Table S6). MNN Correct, ComBat, and limma produced corrected matrices with high TP and low FP rates in DEG analysis. This was followed by ZINB-WaVE and scMerge with slightly lower TP. The TP and FP numbers were further lower for the case of Seurat 3, while scGen and Scanorama gave the worst results with the lowest TP and highest FP. Based on these metrics, ComBat, limma, and MNN Correct were the best methods, followed by ZINB-WaVE and scMerge. Conversely, scGen and Scanorama were the worst performing methods, with Scanorama removing biologically important gene expression difference between cell groups.

When creating the test cases, we used different drop-out rates and sample sizes. As seen in Fig. 20 and Additional file 7: Table S6, with the same batch size and different drop-out rate (case 1 compared to 2, and case 3 compared to 4), the TP and FP counts were on average similar between the case of a high drop-out rate compared to a low drop-out rate, except for scGen. For scGen, there was a big difference in the FP count between low and high drop-out rates, especially in the downregulated genes (FP = 722 in case 3 vs FP = 2763 in case 4, and FP = 92 in case 5 vs FP = 1926 in case 6). Therefore, the drop-out rate had minimal impact on most methods. We also observed slightly higher TP and FP numbers when there was an unbalanced number of cells in the batches (500 cells in batch 1 and 900 cells in batch 2, Fig. 20b), as opposed to balanced cell numbers (500 cells in batch 1 and 450 cells in batch 2, Fig. 20b, Additional file 7: Table S6). However, with a greater imbalance in cell numbers (simulations 5 and 6, with 80 cells in batch 1 and 400 cells in batch 2), the number of TP was instead significantly lower compared to other simulations. Overall, within the tested range of drop-out rates and cell counts, the dropout rate had little impact on the results (TP and FP), while the effect of cell count balance was mixed. Another noteworthy observation is that scGen is prone to FP errors compared to the other methods.

In conclusion, ComBat, MNN Correct, ZINB-WaVE, and scMerge are the recommended methods to obtain a batch-effect-corrected matrix for downstream analysis.

## Discussion

### Batch-effect correction methods

**Fig. 21:**
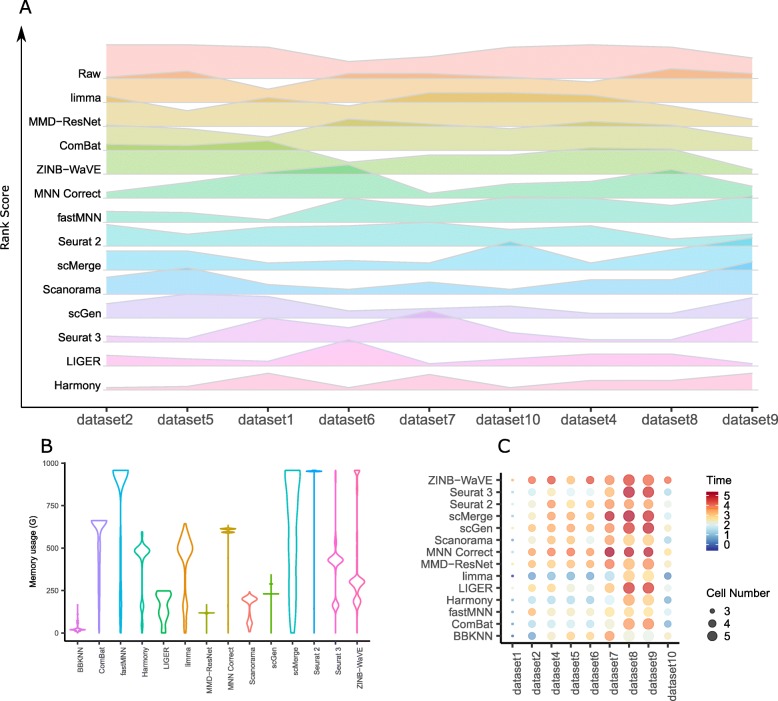
Efficacy and efficiency of the 14 batch-effect correction methods. **a** Rank sum of the assessment metrics. Methods were ranked based on each of the ASW, ARI, LISI, and kBET metrics, and the rankings were then combined across all metrics using the rank sum approach. The height of the ridgelines represents the rank sum scores across different datasets, with a lower rank sum score denoting better performance. Methods are ordered from bottom to top by increasing sum of rank scores across all ten datasets. Thus, methods appearing at the bottom are the best. **b** Memory usage of ten methods on dataset 8. **c** Runtime of 14 methods on ten datasets. Color represents log_10_(time in seconds), node size represents log_10_(cell number)

In the first four scenarios, we tested the batch correction methods’ abilities to mix batches while preserving cell type purity. For each dataset and scenario tested, different methods emerged top. Using the rank sums as summary (Fig. 21a), Harmony, LIGER, and Seurat 3 were the top methods overall. Examination of results for individual datasets shows that Harmony, LIGER, and Seurat 3 were highly ranked in at least four datasets. Harmony was the top method for two datasets in the second scenario on non-identical cell types, and the top method for both datasets in scenario 1 with different technologies. Its performance was poorer in the other scenarios, especially with the large datasets in scenario 4, ranking fourth in only dataset 8. LIGER ranked in the top three in scenario 2 datasets 1, 7, and 10; it also ranked third in dataset 5 and first in dataset 9. Seurat 3 ranked third for dataset 2 and second for dataset 5 in scenario 1, and first for datasets 4 and 8. These three methods were also able to complete runs on the large datasets, making them the best and most promising methods, as scRNA-seq datasets are expected to continue to grow in size. On the other end of the spectrum, ComBat, MMD-ResNet, and limma were the worst performing methods. In particular, limma ranked in the bottom three methods in seven datasets, while MMD-ResNet was in the bottom three for five datasets (Additional file 8: Table S7). Although ComBat was ranked in the bottom three for only two datasets, it was in the bottom half of the rankings in most cases. ZINB-WaVE also featured among the bottom three methods for five datasets with an overall ranking of fourth last.

To recover DEGs from batch-corrected data, we found ComBat, MNN Correct, ZINB-WaVE, and scMerge to be the best methods. Unfortunately, none of these coincide with the top methods for the other four scenarios, since Harmony and LIGER do not return corrected gene expression matrices, while Seurat 3’s DEG recovery was comparatively poorer. While ComBat’s DEG recovery was among the best, it was also one of the worst performing methods in the first four scenarios. Due to the relatively small dataset sizes and in silico simulation which well-defined noise distributions, it is perhaps unsurprising that ComBat performed well. However, it is also unfortunate that method such as Seurat 3 was less successful than older and less sophisticated methods when handling such well-behaved input. Taking into consideration their batch correction performance in the first four scenarios, scMerge is the only leading method for DEG recovery which also gives acceptable batch correction results.

### Batch integration assessment methods

Evaluating batch integration is commonly performed by visually inspecting t-SNE or UMAP plots. While a clear batch integration of small datasets with only a few cell types is easy to evaluate, the assessment can become subjective when the comparison is made between different competitive algorithms on large complicated datasets. The difficulty increases when the batches are not clearly segregated or when similar cell types are mixed. In this study, we used four metrics to help us assess the batch correction methods in terms of batch mixing and cell type purity. Though the assessment metrics may be more objective than visual analysis, the visual plots are still important in experimental applications of exploring newly acquired data and identifying new cell types. Thus, we combined the metrics and visualizations in our analysis.

In the first four scenarios, the assessment metrics did not always agree with each other, nor did the metrics always agree with our visual inspections of t-SNE and UMAP plots. The agreements between iLISI and kBET were generally higher, which can be attributed to their nature of computing based on local neighborhoods. For ARI and ASW, the scores are computed on a more global scale, which can produce significantly different results. Accuracy of the ARI is highly dependent on the clustering labels, which in turn is dependent on the clustering algorithm and number of clusters. For example in dataset 6, the top methods were Scanorama, Harmony, scGen, and scMerge. This was consistent among the metrics and visualizations. However, in the case of MMD-ResNet, it had an excellent ARI_batch_ score, despite its failure to mix Jurkat cells from batches 2 and 3. In this case, the clustering results placed the Jurkat cells in the same cluster, which resulted in the high ARI_batch_ score. Similarly for dataset 1, MMD-ResNet again obtained a good ARI_batch_ score despite the lack of batch integration by visual inspection, and poor ASW_batch_ and iLISI scores. The ASW metric also occasionally gave incongruent results. In dataset 4, ZINB-WaVE obtained the highest ASW_batch_ score. However, the visualizations show poor batch mixing, along with poor performance in iLISI and kBET assessments. Nevertheless, the metrics show broad consensus in most cases and remain useful in assessing specific characteristics of batch-corrected outputs. To smooth out disagreements and obtain an aggregate picture of the metric results, we used the rank sum approach. We also employed the evaluation metrics in conjunction with the visualizations to arrive at a more accurate assessment.

Notably, the currently available metrics only measure batch mixing or cell type purity, e.g., iLISI vs cLISI, ASW_batch_ vs ASW_cell type_, and ARI_batch_ vs ARI_cell type_. To combine batch and cell type assessment, one current approach is to compute a harmonic mean (F1 score). By default, this assumes equal importance on both traits and gives equal weightage, which may not be always appropriate. While it is a challenge to combine different types of assessment methods into a unique evaluation score index, a single integrated index that accounts for both batch mixing and cell type mixing will be better to evaluate the batch-corrected output.

### Runtime and memory requirements

The current state-of-art scRNA-seq experiments are able to generate expression datasets of more than a million cells [27]. Batch correction tools that can scale to such large datasets are needed to meet the challenge of integrating these datasets for large-scale analyses. As part of this benchmarking study, we tested the batch correction methods on dataset 8 with 833,206 cells, and we collected and compared their runtime and memory usage. All runs were conducted on a server equipped with 2x Intel® Xeon® Platinum 8160 CPUs at 2.10 GHz, 1 TB of DDR4 memory, and 1.27 TB of swap memory.

Ten out of 14 methods (BBKNN, ComBat, Harmony, LIGER, limma, MMD-ResNet, Scanorama, scGen, Seurat 3, and ZINB-WaVE) were able to complete runs on datasets 8 and 9, while the remainder did not complete due to insufficient memory or excessively long runtime. In the memory benchmark, BBKNN and MMD-ResNet had the lowest peak memory usage (≤ 170 GB), while Scanorama and LIGER peaked at 250 GB, and the remaining methods consumed more than 500 GB (Fig. 21b). These peak memory usages were observed for fairly small fraction of time process runtimes, which can help reduce resource hogging when running on shared computing resources. However, the peak memory usage does set the overall memory requirement. Drive-based virtual memory can help alleviate system memory shortage, but may increase program runtime.

Memory was the most common barrier to run completion on the large datasets, namely for fastMNN, scMerge, and Seurat 2 (Fig. 21b). While Seurat 3 and ZINB-WaVE also used more than 1 TB of memory, the disk virtual memory available allowed the two methods to finish, albeit with some speed penalty. For fastMNN, scMerge, and Seurat 2, the additional virtual memory usage (1.27 TB) was still insufficient and prevented run completion. MNN Correct required an excessive amount of time, which was also evident in the time required (> 24 h) for downsampled versions of datasets 8 and 9. Therefore, it is unlikely for runs on the full datasets to complete within a reasonable time frame (Fig. 21c).

For the methods that could complete runs on the large datasets, there were significant variations in program runtime (Fig. 21c). Harmony, ComBat, limma, Scanorama, MMD-ResNet, and BBKNN required less than 2 h to complete. ZINB-WaVE required less than 10 h for dataset 8, while the time required for LIGER, scGen, and Seurat 3 ranged from 11.7 h (LIGER on dataset 9) to more than 24 h (Seurat 3 on dataset 8). Among the top performing methods of LIGER, Harmony, and Seurat 3, Harmony was the fastest method, requiring less than 1 h for the big datasets. This makes Harmony a good choice for initial data exploration. If the results are not satisfactory, LIGER is the best alternative. Seurat 3, due to its long runtime, can then be employed if both Harmony and LIGER fail to deliver good results.

## Conclusion

In this study, we designed five testing scenarios with ten datasets to address the problem of removing batch effects. We tested 14 state-of-the-art batch correction algorithms designed to handle single-cell transcriptomic data. We found that each batch-effect removal method has its advantages and limitations, with no clearly superior method. Based on our results, we found LIGER, Harmony, and Seurat 3 to be the top batch mixing methods. Harmony performed well on datasets with common cell types, and also different technologies. The comparatively low runtime of Harmony also makes it suitable for initial data exploration of large datasets. Likewise, LIGER performed well, especially on datasets with non-identical cell types. The main drawback of LIGER is its longer runtime than Harmony, though it is acceptable for its performance. Seurat 3 is also able to handle large datasets, however with 20–50% longer runtime than LIGER. Due to its good batch mixing results with multiple batches, it is also recommended for such scenarios. To improve recovery of DEGs in batch-corrected data, we recommend scMerge for batch correction.

## Methods and materials

Table 1 summarizes the key characteristics of the 14 batch correction methods tested. Most of these methods, except scMerge and scGen, utilize unsupervised learning to find the shared subspace of the datasets. The unsupervised methods do not require cell type information as inputs. scMerge supports both unsupervised and supervised modes, and the unsupervised mode produces reasonably good results. scGen also supports both modes, but the supervised mode gives much better results. In this study, we ran scGen in the supervised mode with cell type information, and all other methods in the unsupervised mode without cell type information. We briefly describe the individual methods below.

**Table 1:** Description of the 14 batch-effect correction methods

Tools	Programming language	Batch-effect-corrected output	Methods	Reference package version
Seurat 2 (CCA, MultiCCA)	R	Normalized canonical components (CCs)	Canonical correlation analysis and dynamic time warping	Butler et al. [4], Seurat package version 2.3.4
Seurat 3 (Integration)	R	Normalized gene expression matrix	Canonical correlation analysis and mutual nearest neighbors-anchors	Stuart et al. [12], Seurat package version 3.0.1
Harmony	R	Normalized feature reduction vectors (Harmony)	Iterative clustering in dimensionally reduced space	Korsunsky et al. [13], Harmony version 0.99.9
MNN Correct	R	Normalized gene expression matrix	Mutual nearest neighbor in gene expression space	Haghverdi et al. [5], Scran package version 1.12.0
fastMNN	R	Normalized principal components	Mutual nearest neighbor in dimensionally reduced space	Haghverdi et al. [5], Lun ATL [7], Scran package version 1.12.0
ComBat	R	Normalized gene expression matrix	Adjusts for known batches using an empirical Bayesian framework	Johnson et al. [1]
limma	R	Normalized gene expression matrix	Linear model/empirical Bayes model	Smyth et al. [2], limma version 3.38.3
scGen	Python	Normalized gene expression matrix	Variational auto-encoders neural network model and latent space	Lotfollahi et al. [16], 2019, scGen version 1.0.0
Scanorama	Python/R	Normalized gene expression matrix	Mutual nearest neighbor and panoramic stitching	Hie et al. [9], Scanorama version 1.4.
MND-ResNet	Python	Normalized principal components	Residual neural network for calibration	Shaham et al. [15] updated code to Python 3
ZINB-WaVE	R	Normalized feature reduction vectors (ZINB-WaVE)/normalized gene expression matrix	Zero-inflated negative binomial model, extension of RUV model	Risso et al. [6], ZINB-WaVE version 1.6.0
scMerge	R	Normalized gene expression matrix	Stably expressed genes (scSEGs) and RUVIII model	Lin et al. [18], scMerge version 1.1.3
LIGER	R	Normalized feature reduction vectors (LIGER)	Integrative non-negative matrix factorization (iNMF) and joint clustering + quantile alignment	Welch et al. [14], liger version 1.0
BBKNN	Python/R	Connectivity graph and normalized dimension reduction vectors (UMAP)	Batch balanced *k-*nearest neighbors	Polański et al. [10], bioRxiv. BBKNN version 1.3.2

### Matching MNN for batch correction (MNN Correct)

MNN Correct searches for mutual nearest neighbors (MNNs) between batches, where cells of similar types across batches share the same neighbors within batches [5]. These identified pairs are then used to compute the batch effect for subsequent correction. The algorithm first pre-filters and scales single-cell gene expression data using cosine normalization, which is robust to data noise. Next, Euclidean distances are computed between cell pairs to identify MNNs. Expression differences between identified cell pairs are next used to compute the batch correction vector, which is then applied to all cells, not just participating cell pairs. The algorithm successively corrects each pair of batches. MNN Correct requires that all batches share at least one cell type with another, which is easy to fulfill. The method also assumes that the batch effects present are smaller and orthogonal to biological variations, which may not always hold true. The published software has the functionality to check whether these assumptions hold true. We first employed the preprocessing functions available in the Seurat package to filter, normalize, and scale the raw data. Based on the MNN tutorial, 5000 highly HVGs were identified and used as input to the mnnCorrect function for batch correction. PCA was then performed on the output matrix to obtain principle components (PCs) for visualization and evaluation.

### Fast matching mutual nearest neighbors for batch correction (fastMNN)

The MNN Correct algorithm demonstrates the efficacy of using MNNs to align data batches [5]. However, the distance computation for nearest neighbor identification is done in the gene expression space and thus computationally demanding. fastMNN [7] is a newer version of MNN Correct where nearest neighbors are determined in the PCA dimensionally reduced space. In our analysis, we employed the Seurat preprocessing workflow to first filter, normalize, and scale the data. Based on the examples provided by fastMNN authors, 5000 HVGs were identified and used as input for projection into the cosine space, followed by multi-sample PCA dimension reduction using the multiBatchPCA function from the Scran package [28] to obtain 50 principal components. Finally, the fastMNN function was used to batch correct the principal component output from the previous step. Visualization and evaluations were performed using the batch-corrected output in PCA space.

### Panorama stitching of single-cell RNA-seq data (Scanorama)

Scanorama also seeks to correct for batch effects through similar cells identified across batches [9]. Approximate singular value decomposition (SVD) is first used to transform the original gene expression data into a dimensionally reduced subspace. An approximate nearest neighbor search is performed using hyperplane locality sensitive hashing and random projection trees to speed up the identification of mutually linked (i.e., nearest neighbor) cells across batches. Unlike MNN Correct that searches for similar cells across batch pairs to compute the correction, Scanorama searches across all batches and determines the priority of dataset merging based on the percentage of matching cells in the batch. Data batches are merged into panoramas using a weighted average of vectors between local matching cells in a fashion similar to MNN Correct. In our analysis, if the gene expression matrix contained raw read counts, we scaled the data by dividing the read counts by the median value of read count sum per cell, followed by log_2_ transform. If the data was already in normalized form, we used it directly as input to the Scanorama function. The batch correction by Scanorama was performed in conjunction with the Scanpy workflow [29]. Finally, the top 20 principal components were extracted from the corrected gene expression matrix and used as input to the assessment methods.

### Batch balanced *k*-nearest neighbors (BBKNN)

BBKNN is another method that first computes the *k*-nearest neighbors in a dimensionally reduced principal component space [10]. The nearest neighbors are identified in a batch-balanced manner using Euclidean distances. BBKNN then transforms the neighbor information into connectivities to construct a graph that links together all cells across batches. The resulting graph is usable for clustering and visualization within the Scanpy workflow. In this study, we used the latest version of BBKNN (version 1.3.2) to perform the analysis. We also tested all of the input options that the BBKNN developers suggest to obtain the best output. We computed UMAP vectors using the Scanpy single-cell analysis platform to project the graph of connectivities into a low dimensionality space for evaluation.

### Seurat alignment CCA, multiCCA (Seurat 2)

Seurat multiCCA uses canonical correlation analysis (CCA) to first compute the linear combinations of genes with maximum correlation between batches [4]. These vectors are then aligned with dynamic time warping to account for population density changes, resulting in a single low-dimensional subspace with all input data sets. In our analysis, following the Seurat tutorial, we first employed the preprocessing functions from the Seurat package (v2.3.4) to filter, normalize, and scale the raw data. Two thousand HVGs were then identified for further use. Next, we used the CCA and multiCCA functions, for two batches and more than two batches respectively, to transform the data into CCA space. This was followed by the AlignSubSpace function to perform batch-effect correction. The output was then transformed into PCA space for further evaluation and visualization.

### Seurat Integration (Seurat 3)

Seurat Integration (Seurat 3) is an updated version of Seurat 2 that also uses CCA for dimensionality reduction [12]. Unlike Seurat 2, Seurat 3 first identifies MNNs (referred to as “anchors”) of similar cell states across batches in the normalized CCA subspace. To avoid aberrant anchors between dissimilar cells, the shared nearest neighbor graphs are used to assess cell type similarity. Similar to MNN, a correction vector is computed using the difference in expression profiles between cells to perform the data transformation. To handle more than two batches, a guide tree hierarchy based on pair-wise data similarities guides the batch integration order. In our analysis, we used the Seurat package version 3.0.1 in the R language environment to perform batch-effect correction. Adhering to the suggested integration workflow, we first scaled each dataset, selected 2000 HVGs as input to compute the integration anchors (FindIntegrationAnchors), and then integrated (IntegrateData) the batches using the anchors. The output was finally transformed into PCA space for further evaluation and visualization.

### Unsupervised joint embedding method (Harmony)

Harmony uses an iterative clustering approach to align cells from different batches [13]. The algorithm first combines the batches and projects the data into a dimensionally reduced space using PCA. Harmony then uses an iterative procedure to remove the multi-dataset-specific effects. Each iteration consists of four steps: the algorithm first groups the cells into multiple-dataset clusters using a novel variant of soft k-means clustering that allows fast and flexible cell clustering. In the second step, Harmony computes a global centroid for each cluster and a centroid for each specific dataset. Thirdly, using the centroids, Harmony calculates a correction factor for each dataset. Finally, the correction factor is used to correct each cell with a cell-specific factor. This procedure is applied repeatedly until convergence. In our analysis, following examples provided in the Harmony package in R language environment, we ran Harmony within the Seurat 2 workflow with the maximal number of clusters (50) and the maximal number of iterations (100). The top 20 normalized Harmony vectors in PCA space were used as input to the assessment methods.

### Distribution-matching residual networks (MMD-ResNet)

Shaham et al. proposed a deep learning approach for removing batch effects based on the residual neural network algorithm, or “MMD-ResNet” in 2016 [15]. This approach is based on the assumption that the difference between the distributions of the two datasets is moderate. Under this assumption, the key idea is to train a residual network to learn a map from one distribution to another. This map is then used to calibrate the source dataset to target dataset. Deciding which dataset serves as the source and which one serves as the target affects the result of batch-effect correction. In our analysis, we tested both orientations and compared the outputs to find the better option. Following the suggested pipeline, we first computed 50 principal components and used the reduced feature to train a network using 100 epochs with the batch size set to 30 for the small datasets (less than 1000 cells) and 50 for the larger datasets.

### scGen

scGen is a transfer learning method that combines variational auto-encoders (VAEs) and latent space vector arithmetic to model and predict single-cell expression data [16]. scGen first uses deep neural networks with VAEs to build a model that learns the distribution of cells in the reference dataset. Thereafter, the trained network is used to predict the distribution of the query dataset. The specific network architecture allows scGen to efficiently correct batch effects. In our analysis, we used scGen version 1.0.0 within the Scanpy pipeline in the Python environment. We trained a scGen network in 100 epochs with the batch size set to 30 for small datasets (less than 1000 cells) and 50 for larger datasets. Finally, we extracted 20 PC vectors from the corrected gene expression matrix and used them as input to the assessment and visualization methods.

### Adjust for batch effects using an empirical Bayes framework (ComBat)

ComBat was originally developed for microarray gene expression data [1], but had been successfully employed on scRNA-seq data [6]. Expression data is first standardized so that all genes have similar means and variances. Thereafter, the standardized data is fitted to standard distributions using a Bayesian approach to estimate the batch effects present. The computed batch-effect estimators are then used to correct the original expression matrix. There are two modes to run ComBat, which are parametric and non-parametric adjustments to correct for batch effects. Parametric mode includes scale adjustments whereas non-parametric mode only corrects the mean of the batch effect. In our benchmarking, we tested both modes, compared the results, and selected the better output from the two modes for comparison with other methods. In most cases, the parametric mode gave better output than the non-parametric mode. In our analysis, we computed the median value of sum of gene expression per cell, then used this median value to scale the data and then performed log_2_ transform. The scaled data was subsequently used as input to the ComBat function. Finally, we calculated 20 PC vectors from the corrected expression matrix as input to the assessment methods.

### Linear models for RNA-seq and microarray data (limma)

Similar to ComBat, limma was initially designed to work with microarray data [2]. limma fits the input data to a linear model with a blocking term to capture the batch effects. The batch effect captured in the term is then subtracted from the original data to obtain the batch-corrected expression matrix. In our work, we employed the preprocessing workflow in Seurat 2 to filter, normalize, and scale the data. Subsequently, the scaled data was used as input to the limma batch-effect removal function. Finally, 20 PCs were computed from the limma normalized matrix as input to the assessment methods.

### Zero-inflated negative binomial model for RNA-seq data (Zinb-WaVE)

Risso et al. [6] proposed an extension of the remove unwanted variation (RUV) model to use the zero-inflated negative binomial (ZINB) regression to model data with technical and biological effects. The resulting model is able to estimate zero inflation (dropouts), over-dispersion, and distribution of the data. ZINB-WaVE fits a ZINB model to the data, resulting in a factor model similar to PCA. Batch effect present is then removed from the raw data to produce a corrected gene expression matrix. In this study, we employed the Seurat preprocessing workflow to first filter, scale, and normalize the raw data. Thereafter, we extracted HVGs from the log transformed data for use as input to batch correction. Due to speed issues with the zinbwave function on our datasets, we used the zinbsurf function, which employs an approximate strategy that uses only a random subset of cells to infer the distribution for projecting the full dataset. For the DEG study with dataset 3, we used the corrected gene expression matrix output for further analysis. For other datasets, we obtained the reduced features vectors of ZINB, and computed PC vectors from them. The PC vectors were then used for evaluation and visualization.

### Merging multiple batches of scRNA-seq data (scMerge)

scMerge first searches for mutual nearest clusters in data batches using batch-specific HVGs to construct a graph that links cell clusters between batches [18]. Stably expressed genes (SEGs) are also identified for use as negative controls to estimate unwanted factors. The RUV model is then used to capture unwanted variations between datasets for removal. In the semi-supervised mode, cell type information is used to merge identified cell clusters according to type. In our benchmarking, we ran scMerge in the unsupervised mode because cell type information is often unavailable in experimental data, and most of the other batch correction methods were designed to function in an unsupervised manner. For the preprocessing step, we normalized the input data by log_2_ transform. For datasets 2 and 4, the cosine standardization was performed separately on each batch due to the different sequencing platforms used for data acquisition and the resulting large variance in gene expression across batches.

### A platform for integration of gene expression, epigenetic regulation, and spatial relationships across single-cell datasets (LIGER)

LIGER employs an iterative learning approach to characterize batch data for correction [14]. The method uses integrative non-negative matrix factorization (iNMF) to first learn a low-dimensional space where each gene is characterized by two sets of factors. The first set contains dataset-specific factors, and the second contains shared factors. The shared factor space is then used to identify similar cell types across datasets by first constructing a shared factor neighborhood graph to connect cells with similar factor loading patterns. Joint clusters are then identified using the Louvain community detection. Thereafter, the factor loading quantiles are normalized using the largest data batch as reference to achieve batch-correction. In our work, we followed the LIGER documentation. For preprocessing, we used the LIGER preprocessing functions, where we first selected genes with high variance. We then performed iNMF-based factorization using an alternating least squares algorithm (with number of factors *k* = 20 and the penalty parameter *λ* = 5), followed by data alignment using joint clustering and quantile alignment.

### Datasets

In this paper, we used experimentally derived and computer-simulated datasets to evaluate the performance of each batch correction method. Data sources, data batches, cell counts, and acquisition technology are listed in Additional file 1: Table S1, and the cell counts per cell type are listed in Additional file 2: Table S2. The ten datasets are described below:

#### Dataset 1: human dendritic cells

Dataset 1 consists of human blood dendritic cell (DC) scRNA-seq data from Villani et al. [30]. Villani et al. sorted CD1C DC, CD141 DC, plasmacytoid DC (pDC), and double negative cells, and then analyzed each population using Smart-Seq2 in two batches. We extracted the transcript per million (TPM) values of these four cell populations from GSE94820_raw.expMatrix_DCnMono.discovery.set.submission.txt.gz downloaded from https://www.ncbi.nlm.nih.gov/geo/query/acc.cgi?acc=GSE94820. Cell type and batch information were extracted using the cell ID which is formatted as “Cell.Type”_“Plate.ID”_“single.cell.ID.” Plates “P7”, “P8”, “P9”, and “P10” were run as batch 1, and “P3”, “P4”, “P13”, and “P14” as batch 2. The data batches have low sparsity (zero counts) and contain well-known cell types. We then removed CD1C DC in batch 1 and CD141 DC in batch 2 such that the two batches have non-identical cell types. Both of the resulting batches consist of 288 cells and 16,594 genes each. The first batch has 96 pDC, 96 double negative, and 96 CD141 cells. The second batch has 96 pDC, 96 double negative, and 96 CD1C cells. Notably, both batches only share two cell types (pDC and double negative), and each batch has one unshared type (CD141 and CD1C respectively) that are also biologically similar. This data characteristic creates an interesting challenge for batch correction algorithms to integrate the common cell types across batches while maintaining separation between highly similar cell types in different batches.

#### Dataset 2: mouse cell atlas

Two independent mouse cell atlas datasets were generated by Han et al. [31] using Microwell-Seq, and by the Tabula Muris Consortium [32] using 10x Genomics and Smart-Seq2 protocols. For the dataset generated by Han et al., the Digital Gene Expression (*DGE*) matrix was extracted from MCA_Figure2batchremoved.txt.tar.gz downloaded from https://ndownloader.figshare.com/files/10351110?private_link=865e694ad06d5857db4b; cell type information was extracted from MCA_Figure2batchremoved.txt.tar.gz downloaded from https://ndownloader.figshare.com/files/10760158?private_link=865e694ad06d5857db4b. The Tabula Muris Consortium generated both 10x Genomics and Smart-Seq2 data, but only the Smart-Seq2 data was used in our study. Read counts were extracted from FACS.zip downloaded from https://ndownloader.figshare.com/files/10038307; cell type information was extracted from annotations_FACS.csv downloaded from https://ndownloader.figshare.com/files/10039267. Only cells with cell type labels and genes that were detected in both Microwell-seq and Smart-Seq2 data were retained for further processing. We selected 11 cell types with high cell numbers that were present in both batches. The selected cell types originate from nine organ systems: urinary, digestive, respiratory, circulatory, muscular, immune, nervous, endocrine, and lymphatic. The resulting first batch contains 4239 cells, and the second batch consists of 2715 cells, with 15,006 genes in both gene count tables. This dataset evaluates the removal of batch effects induced by using different scRNA-seq technologies.

#### Dataset 3: simulation

Due to the large variance in gene expression values found in experimentally obtained scRNA-seq data from different batches, the true differentially expressed genes (DEGs) are difficult to be determined. Therefore, we employed simulated data with known ground truth DEGs to assess the impact of batch-effect correction on DEG detection. We simulated 6 sets of single-cell read-count data using the Splatter package [19]. Each set contains two batches with balanced or unbalanced numbers of cells (Fig. 20b). Both batches contain 5000 genes. Due to the low capture efficiency common in scRNA-seq data, 5 or 25% of the cells were simulated to experience “drop out” events.

#### Dataset 4: human pancreas

Dataset 4 was constructed using human pancreatic data from five different sources [33–37]. The data batches were downloaded in the form of homogeneously prepared Single Cell Experiment (SCE) R objects featuring standardized annotations from https://hemberg-lab.github.io/scRNA.seq.datasets/human/pancreas/. For the data batches generated by Baron et al. and Segerstolpe et al., counts were extracted from the SCE R objects and used for further processing. For datasets generated by Muraro et al., Wang et al., and Xin et al., normcounts were extracted from the SCE R objects and further processed. Only genes that were detected in all five experiments were kept. Cell type information was also extracted from the SCE R objects. Cells annotated as “unclear,” “co-expression,” “not applicable,” “unclassified,” “unclassified endocrine,” “dropped,” “alpha.contaminated,” “beta.contaminated,” “delta.contaminated,” or “gamma.contaminated” were removed. “activated_stellate,” “PSC (Pancreatic Stellate Cell,” and “quiescent_stellate” cells were renamed to “stellate.” “mesenchymal” and “mesenchyme” cells were renamed to “mesenchymal.” The resulting dataset consists of five batches acquired using four different scRNA-seq technologies, with 15 different cell types for a total of 14,767 cells with 15,558 genes each. This dataset was used to assess batch-effect correction across multiple (> 2) data batches.

#### Dataset 5: human peripheral blood mononuclear cell (PBMC)

Dataset 5 is made up of human PBMC scRNA-seq data [38]. The 3′ and 5′ 10x Genomics protocols which capture different regions of mRNA were used to generate the two data batches. *Unique Molecular Identifiers* (*UMI*) counts of both batches were downloaded from the 10x Genomics website. The 3′ data was obtained from https://support.10xgenomics.com/single-cell-gene-expression/datasets/2.1.0/pbmc8k. For this data batch, PBMCs of a healthy donor were analyzed using the *Chromium* Single Cell 3′ v*2* chemistry in the experiment, and UMI counts were extracted using CellRanger 2.1.0. After filtering in CellRanger, 8381 cells were detected. The 5′ data was obtained from https://support.10xgenomics.com/single-cell-vdj/datasets/2.2.0/vdj_v1_hs_pbmc_5gex. In this data batch, PBMCs of a healthy donor were analyzed using the Chromium Single Cell 5′ paired-end chemistry, and UMI counts were extracted using CellRanger 2.2.0. After CellRanger-based filtering, 7726 cells were detected. The cells were annotated using the annotation published in Polański et al. [10]. Polański et al. selected cells each with the number of unique genes between 500 and 7000, and a total UMI count above 2000, and then annotated the cells in k-nearest neighbor clusters based on canonical markers. The cell type information by Polański et al. was downloaded from ftp://ngs.sanger.ac.uk/production/teichmann/BBKNN/PBMC.merged.h5ad and read using the script downloaded from https://nbviewer.jupyter.org/github/Teichlab/bbknn/blob/master/examples/pbmc.ipynb. Cells that were not annotated by Polański et al. were removed from our analysis. The resulting dataset consists of 8098 cells for the 3′ batch and 7378 cells for the 5′ batch, each with 17,430 genes. Integration of these two batches measures the integration capacity of the methods when encountering protocol-driven biological differences.

#### Dataset 6: cell line

The cell ranger output files of the cell line experiment were obtained from the 293t_jurkat subfolder of the data downloaded from http://scanorama.csail.mit.edu/data.tar.gz [9, 38]. We used the Read10X function of the Seurat package to extract the UMI count table from the Cell Ranger output files. Cell type information was extracted from the 293t_jurkat_cluster.txt file in the cell_labels subfolder. The dataset is composed of three batches, where batch 1 contains only 293T cells (2885 cells), batch 2 contains only Jurkat cells (3258 cells), and batch 3 consists of a 50/50 mixture of Jurkat and 293T cells (3388 cells). The gene expression data contains 16,602 genes acquired using the 10x Genomics platform. This dataset is suitable for assessing batch correction as the population of each cell type is monoclonal and the populations biologically dissimilar.

#### Dataset 7: mouse retina

Dataset 7 is composed of mouse retina data generated using the Drop-seq technology by two unassociated laboratories [39, 40]. The data batches were downloaded in the form of homogeneously prepared Single Cell Experiment (SCE) R objects featuring standardized annotations from https://hemberg-lab.github.io/scRNA.seq.datasets/mouse/retina/. Cell type information and counts were first extracted from the SCE R objects. Genes detected in both batches were retained, and cells of unknown type were removed. The resulting first data batch (Shekhar et al. [39]) has 26,830 cells, and the second batch (Macosko et al. [40]) has 44,808 cells, both with 12,333 genes. While the tissue of origin is the same for both batches, the cell types are not identical between batches. This dataset evaluates batch correction of a fairly large dataset with non-identical cell types.

#### Dataset 8: mouse brain

We combined two different mouse brain datasets from Saunders et al. [41] and Rosenberg et al. [42] to test the batch correction methods on a big dataset [9]. The two data batches were generated using different technologies, the Drop-seq and SPLiT-seq protocols respectively. There are 691,600 cells in batch 1, and 141,606 cells in batch 2, with 17,745 common genes. This dataset was used to evaluate the removal of batch effects induced by using different scRNA-seq technologies on a big dataset. We downloaded the data by Saunders et al. from http://dropviz.org/ and extracted the Digital Gene Expression (DGE) matrices of cells from the .raw.dge.txt.gz files found under the “DGE By Region” section. Cells were first assigned to clusters and sub-clusters according to the .cell_cluster_outcomes.RDS files also downloaded from the “DGE By Region” section, and cell type annotation was then incorporated based on the assigned clusters and sub-clusters found in the BrainCellAtlas_Saunders_version_2018.04.01.RDS file downloaded from https://storage.googleapis.com/dropviz-downloads/static/annotation.BrainCellAtlas_Saunders_version_2018.04.01.RDS. We removed cells with unknown cell type information and renamed “Endothelial_stalk” and “Endothelial_tip” cells to “Endothelial.” We downloaded the data by Rosenberg et al. from https://www.ncbi.nlm.nih.gov/geo/query/acc.cgi?acc=GSM3017261. Digital gene expression (DGE) matrices and cluster information were extracted from GSM3017261_150000_CNS_nuclei.mat.gz using the script made available by the author, at https://gist.github.com/Alex-Rosenberg/5ee8b14ea580144facad9c2b87cebf10. We then renamed the clusters with cell type information from Fig. 2 in Rosenberg et al.: clusters 1–54 were renamed to “Neuron,” 55–60 to “Oligodendrocytes,” 61 to “Polydendrocytes,” 62 to “Macrophage,” 63 to “Microglia,” 64 to “Endothelial,” 65 to “Mural,” 66–67 to “Vascular and leptomeningeal cells,” 68–71 to “Astrocyte,” 72 to “Ependymal,” and 73 to “Olfactory ensheathing cells.”

#### Dataset 9: human cell atlas

This is a large dataset with 321,463 bone marrow cells in batch 1 and 300,003 cord blood-derived cells in batch 2 [43]. Both data batches were generated using the 10x Genomics protocol with 18,969 genes acquired for each cell. Unfortunately, no appropriate cell type annotation was available. This dataset was used to gauge the assimilation power on big datasets of different tissue type generated with one scRNA-seq technology. The raw count matrices were downloaded from https://preview.data.humancellatlas.org/ (cord blood: https://s3.amazonaws.com/preview-ica-expression-data/ica_cord_blood_h5.h5; bone marrow: https://s3.amazonaws.com/preview-ica-expression-data/ica_bone_marrow_h5.h5).

#### Dataset 10: mouse hematopoietic stem and progenitor cells

The two data batches of this dataset were acquired by Nestorowa et al. [44] using the SMART-seq2 protocol (GSE81682, 1920 cells), and Paul et al. [45] using the MARS-seq protocol (GSE72857, 10,368 cells). We replicated the preprocessing and filtering workflow for the two datasets as described by Haghverdi et al. [5]. Of the 10,368 cells in the MARS-seq generated dataset, the 2729 well-annotated cells were extracted. We also modified the “ESLAM” cell type labels to “HSPC,” “LT-HSC” to “LTHSC,” and “ERY” to “MEP.” The genes were also filtered as per the published workflow; the list of HVGs from Nestorowa et al. was downloaded and compared with the dataset from Paul et al. The resulting common list of 3467 genes was retained in both datasets for further use. This dataset tests the ability of batch correction methods to eliminate batch effects due to different sequencing technologies in data batches with non-identical cell types.

### Evaluation functions

To help guide the assessment of batch correction algorithm efficacy, we employed five different methods, kBET, LISI, ASW, ARI, and DEG analysis. After obtaining the batch-corrected outputs, we computed the PCA vectors and used the top 20 PC as inputs to calculate the respective kBET, LISI, ASW, and ARI scores. For LISI, ASW, and ARI, we first calculated the metrics for assessing cell type purity and batch mixing separately, and then combined the assessments into a F1 score, as described in the following subsections. To summarize the metrics for comparison, we computed their rank sum. For DEG analysis, we used the normalized gene expression matrix obtained from each batch-effect removal method as input.

### *k*-Nearest neighbor batch-effect test (kBET)

The first metric employed is the kBET [23], which measures batch mixing at the local level. After SVD-based dimension reduction, the *k*-nearest neighbors around each data point are selected to compute its local batch label distribution. Then, 10% of the data points are randomly selected to test the local batch label distribution against the global distribution. The null hypothesis of all batches being well mixed is not rejected if the local distribution is sufficiently similar to the global distribution. The fraction of rejections ranges from 0 to 1. If the fraction of rejections is close to zero, this signifies that the batches are well mixed. In this work, we generated PCs from the corrected gene expression matrix, or from the corrected dimensionally reduced cell embeddings. Then, we used top 20 vectors as input to the kBET function. Because the number of nearest neighbors *k* as input has a big impact on the results of kBET, we ran kBET using a predefined list of *k* values. Following the example in the kBET paper, we chose the *k* input value equal to 5%, 10%, 15%, 20%, and 25% of the sample size and ran kBET to get the median of all kBET rejection rates to produce the final kBET result for each method. Finally, the Wilcoxon statistical test with the Benjamini and Hochberg correction was performed on the kBET results to identify if the integrated output of a method is statistically significantly better than other methods.

### Local inverse Simpson’s index (LISI)

Another local level metric is the LISI proposed by Korsunsky et al. [13, 22], which can be used to assesses batch and cell type mixing. Instead of a fixed number of nearest neighbors as in the case of kBET, LISI selects the nearest neighbors based on the local distribution of distances with a fixed perplexity. The selected neighbors are then used to compute the inverse Simpson’s index for diversity, which is the effective number of types present in this neighborhood. In the case of LISI integration (iLISI) to measure batch mixing, the index is computed for batch labels, and a score close to the expected number of batches denotes good mixing. The iLISI score is only computed for cells whose type appears in all batches. For cell type LISI (cLISI), the index is computed for all cell type labels, and a score close to 1 denotes that the clusters contain pure cell types. We computed the iLISI and cLISI scores for each cell in the dataset, and then determined the median values. To scale the median scores, we used the respective maximum and minimum scores. For combined assessment of cell type purity and batch mixing, the harmonic mean of cLISI and iLISI was computed to obtain the F1 score as described by Lin et al. [18]:

$$ \mathrm{F}{1}_{\mathrm{LISI}}=\frac{2\left(1-{\mathrm{cLISI}}_{\mathrm{norm}}\right)\left({\mathrm{iLISI}}_{\mathrm{norm}}\right)}{1-{\mathrm{cLISI}}_{\mathrm{norm}}+{\mathrm{iLISI}}_{\operatorname{norm}.}} $$

A higher F1 score indicates superior batch correction. Finally, Wilcoxon statistical test with Benjamini and Hochberg correction was performed on the iLISI and cLISI results to identify if any method(s) is statistically significantly better than others.

### Average silhouette width (ASW)

We employed the ASW [16, 24] in conjunction with cell type and batch labels to assess batch correction. The silhouette score of a data point is computed by subtracting its average distance to other members in the same cluster from its average distance to all members of the neighboring clusters, and then dividing by the larger of the two values. The resulting score ranges from − 1 to 1, where a high score denotes that the data point fits well in the current cluster, while a low score denotes a poor fit. The average score of all data points is used to measure overall cell type purity or batch mixing through the choice of labels.

In our work, we first randomly subsampled our datasets to 80% of the original number of cells. We then used the first 20 PCs of the downsampled datasets as input to calculate the distances between data points to obtain the ASW scores. To ensure the stability of ASW scores, we repeated this process 20 times to obtain 20 ASW scores each for batch mixing and cell type mixing. The median values for batch and cell type mixing were then used for further computation. We reversed the batch ASW score (higher is better) and then normalized both ASW scores to between 0 and 1. For combined assessment of cell type purity and batch mixing, we calculated the harmonic mean of batch and cell type ASW scores to obtain the F1 score:

$$ \mathrm{F}{1}_{\mathrm{ASW}}=\frac{2\left(1-{\mathrm{ASW}}_{\mathrm{batch}\_\operatorname{norm}}\right)\left({\mathrm{ASW}}_{\mathrm{cell}\_\mathrm{type}\_\operatorname{norm}}\right)}{1-{\mathrm{ASW}}_{\mathrm{batch}\_\operatorname{norm}}+{\mathrm{ASW}}_{\mathrm{cell}\_\mathrm{type}\_\operatorname{norm}}} $$

A higher F1 score (that is, smaller ASW score for batch and higher ASW score for cell type) indicates superior batch correction. Finally, the Wilcoxon statistical test with Benjamini and Hochberg correction was performed on the ASW results to identify if any method(s) is statistically significantly better than others.

### Adjusted rand index (ARI)

The ARI [25] can also be employed to evaluate batch correction methods in terms of cell type purity and batch mixing. The ARI measures the percentage of matches between two label lists, corrected for chance. In our work, we first subsampled our datasets to 80% of their original number of cells. We then used 20 PCs of the subsampled corrected data to perform *k*-means clustering using the *k*-means function from the stats package in the R language environment (where *k* is the number of unique cell types). To assess cell type purity using ARI, the cell type labels were compared against the *k*-means clustering results using the adjustedRandIndex function of the mclust R package [46]. A high ARI score corresponds to high cell type purity. For batch mixing assessment, only cells whose types are present in all batches were considered. Their respective batch labels were compared to the *k*-means clustering labels, and a low ARI score denotes superior mixing. Similar to the silhouette coefficient, to produce stable results, the computations for batch and cell type assessments were repeated 20 times each with random subsampling. The median ARI scores were then normalized to range between 0 and 1, and a combined F1 score was obtained for each batch correction method by computing the harmonic mean of the ARI scores:

$$ \mathrm{F}{1}_{\mathrm{ARI}}=\frac{2\left(1-{\mathrm{ARI}}_{\mathrm{batch}\_\operatorname{norm}}\right)\left({\mathrm{ARI}}_{\mathrm{cell}\_\mathrm{type}\_\operatorname{norm}}\right)}{1-{\mathrm{ARI}}_{\mathrm{batch}\_\operatorname{norm}}+{\mathrm{ARI}}_{\mathrm{cell}\_\mathrm{type}\_\operatorname{norm}}} $$

A higher F1 score will result from a lower ARI batch mixing score, and a higher ARI cell type mixing score. Finally, the Wilcoxon statistical test with the Benjamini and Hochberg correction was performed on the ARI results to test if a method is statistically significantly better than other methods.

### Identification of highly variable genes for simulated dataset 3

Highly variable genes enable us to highlight biological significance in downstream analysis. Consequently, batch-effect removal methods mainly focus on the correction of these HVGs. Using simulated data, we investigated the capacity of each method in two cases: using all genes, or only HVGs as input. The HVGs were selected based on dispersion and mean of expression. We employed the commonly used FindVariableGenes function in Seurat 2.3. [4] with 0.0125 as the low cutoff for mean of expression, 3.0 as the high cutoff for mean of expression, and an expression dispersion cutoff of 0.5.

### Differential gene expression analysis (DEG)

To perform DEG analysis, we used the FindMarkers function from the Seurat 2 package [4], with the likelihood-ratio test for single-cell gene expression (bimodal test). *p* value adjustment was performed using the Bonferroni correction with an adjusted *p* value less than 0.05 as the threshold. We performed DEG analysis on the gene expression matrix of raw data, which contains batch effects, and on the batch-corrected expression matrices (Seurat 3, MNN Correct, ComBat, limma, scGen, Scanorama, ZINB-WaVE, and scMerge).

### Accuracy metric of identification of DEGs

DEGs detected in the batch-corrected expression matrices of all genes or HVGs were compared to the ground true DEGs, and evaluation metrics including TP, FP, FN, TN, precision, and *F*-score were calculated. True positives (TP) is the number of genes which were detected as significant and are also true DEGs. False positives (FP) is the number of genes which were detected as significant but not true DEGs. False negatives (FN) is the number of genes which are true DEGs but detected as non-significant. True negatives (TN) is the number of genes that are not true DEGs and were detected as non-significant. Precision is defined as TP/(TP + FP), and *F*-score (measuring accuracy) is defined as (TP + TN)/(TP + TN + FP + FN). TP, FP, FN, TN, precision, and *F*-score were computed for each simulated dataset, and the median *F*-score over 6 simulated datasets was used to rank the batch correction methods.

### t-Distributed stochastic neighbor embedding (t-SNE) visualization

We employed t-SNE [20] to visualize our batch correction results. We ran t-SNE with a perplexity of 30 using the Scanpy package in the Python environment to visualize the raw data and batch-corrected output.

### Uniform manifold approximation and projection (UMAP) visualization

We employed UMAP [21] to visualized our batch correction results. We ran UMAP with the default number of neighbors using the Scanpy package in the Python environment to visualize the raw data and batch-corrected output.

### Assessment of memory usage

Memory usage becomes more and more of a critical challenge as the input data size increases*.* We assessed the memory usage of the 14 methods with dataset 8, which is the largest. We ran the assessment on our server with 1 TB of RAM and recorded the memory usage every 5 s. We then visualized the memory usage in the form of violin plots (Fig. 21b). Some methods were unable to complete the batch correction runs due to memory (> 1 TB) or runtime (> 48 h) requirements. Only BBKNN, ComBat, Harmony, limma, ResNet, Scanorama, scGen, LIGER, and Seurat 3 were able to complete the runs, and we recorded their memory usage during the entire run; fastMNN, scMerge, Seurat 2, and ZINB-Wave were terminated halfway because they consumed more than 1 TB of memory, and MNN Correct was also terminated because its runtime was too long.

### Computation evaluation of runtime

We captured the runtime of each method using the time function available in R and Python environments. We did not take into account the pre-filtering steps, and only measured the runtime of the main function in each method. All jobs were run on a Linux server with 2x Intel® Xeon® Platinum 8160 CPUs at 2.10GHz, 1 TB of DDR4 memory, and 1.27 TB of swap memory.

## Supplementary information

**Additional file 1: Table S1.** Detailed description of datasets. The table lists the dataset sources, number of batches, number of cells per batch, and sequencing technology.

**Additional file 2: Table S2.** Cell count per cell type. Breakdown of cell count per cell type for each dataset.

**Additional file 3: Table S3.** Scenarios and datasets. The table lists the datasets and which scenario each fall under. Each dataset may qualify for more than one scenario.

**Additional file 4: Figure S1-S11.** with legends.

**Additional file 5: Table S4.** Evaluation metrics. Detailed assessment metric scores and *F*-score for all methods on all datasets.

**Additional file 6: Table S5.** Evaluation statistical test. Statistical significance test results of the batch correction method’s assessment metric scores.

**Additional file 7: Table S6.** Simulation statistical test. Statistical significance test results of the DEG recovery with simulated data.

**Additional file 8: Table S7.** Rank and rank sums. The ranking of the batch correction methods for ARI, ASW, kBET, and LISI assessment metrics, and the rank sums for each method and the derived final rank for each dataset.

**Additional file 9.** Review history and authors’ response. Reviewers’ comments and authors’ point-to-point response.

## Data Availability

All data can be freely downloaded and are described in Additional file 1: Table S1 and in Methods. Dataset 1 consists of human blood dendritic cells (DC) scRNA-seq data from GEO accession GSE80171 [30]. Two independent mouse cell atlas datasets of dataset 2 are found at GEO under accession GSE108097 [31] and GSE109774 [32]. Dataset 4 was constructed using human pancreatic data from five different sources, available under accession numbers GSE85241 [33], E-MTAB-5061 [34], GSE84133 [35] GSE83139 [36], and GSE81608 [37]. Dataset 5 is made up of human PBMC scRNA-seq data [38]. For dataset 6, the cell ranger output files of the cell line experiment were obtained from the 293t_jurkat subfolder of the data downloaded from http://scanorama.csail.mit.edu/data.tar.gz [9, 38]. Dataset 7 is composed of mouse retina data found in GEO at GSE81904 and GSE63473 [39, 40]. Dataset 8 combines mouse brain data obtained from GEO accession ids GSE116470 [41] and GSE110823 [42]. Dataset 9 is obtained from the Human Cell Atlas [43]. The two data batches of dataset 10 were acquired from GEO GSE81682 [44] and GSE72857 [45] The simulation data are available at https://github.com/JinmiaoChenLab/Batch-effect-removal-benchmarking [47].
