# Supplementary material for: A benchmark of batch-effect correction methods for single-cell RNA sequencing data
Source: Genome Biol. 2020 Jan 16;21:12. doi: 10.1186/s13059-019-1850-9 (PMC6964114; doi:10.1186/s13059-019-1850-9)
Supplement: Supplementary file 3 — Additional file 3: Table S3. Scenarios and datasets. The table lists the datasets and which scenario each fall under. Each dataset may qualify for more than one scenario. [file 13059_2019_1850_MOESM3_ESM.docx]

**Table S3: Dataset scenarios**

Ten datasets that cover five different scenarios

| Dataset no. | Description | Scenario | | | | |
| --- | --- | --- | --- | --- | --- | --- |
|  |  | 1. Identical cell types different technologies | 2. Non-identical cell types | 3. Multiple batches | 4. Big data | 5. Simulation |
| 1 | Human Dendritic Cells |  | √ |  |  |  |
| 2 | Murine Atlas | √ |  |  |  |  |
| 3 | Simulations |  |  |  |  | √ |
| 4 | Human Pancreas |  | √ | √ |  |  |
| 5 | Human Peripheral Blood Mononuclear Cell | √ |  |  |  |  |
| 6 | Cell line |  | √ | √ |  |  |
| 7 | Mouse Retina |  | √ |  | √ |  |
| 8 | Mouse Brain |  | √ |  | √ |  |
| 9 | Human Cell Atlas |  |  |  | √ |  |
| 10 | Mouse Haematopoietic Stem and Progenitor Cells |  | √ |  |  |  |
