# Supplementary material for: A benchmark of batch-effect correction methods for single-cell RNA sequencing data
Source: Genome Biol. 2020 Jan 16;21:12. doi: 10.1186/s13059-019-1850-9 (PMC6964114; doi:10.1186/s13059-019-1850-9)
Supplement: Supplementary file 4 — Additional file 4: Figure S1-S11. with legends. [file 13059_2019_1850_MOESM4_ESM.docx]

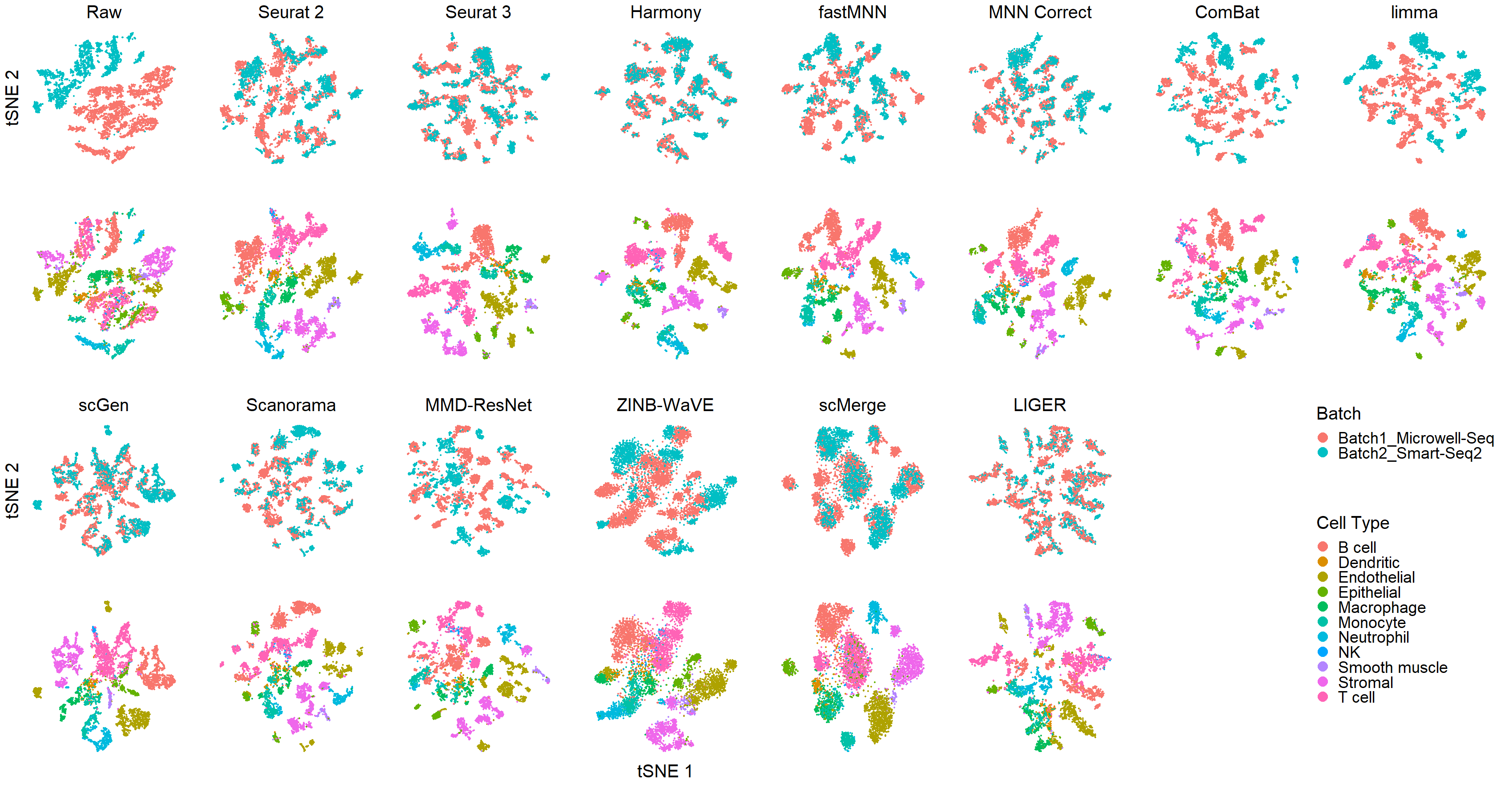
 **Fig S1: Qualitative evaluation of fourteen batch effect correction methods using tSNE visualization for Dataset 2 Mouse Cell Atlas.** The 14 methods are organized into two panels, the top panel showing t-SNE of raw data, Seurat 2, Seurat 3, Harmony, fastMNN, MNN Correct, ComBat, and limma, while the bottom panel shows t-SNE of scGen, Scanorama, MMD-ResNet, ZINB-WaVE, scMerge, and LIGER. Each panel contains two rows of t-SNE plots. In the first row cells are colored by batch and in the second by cell type.


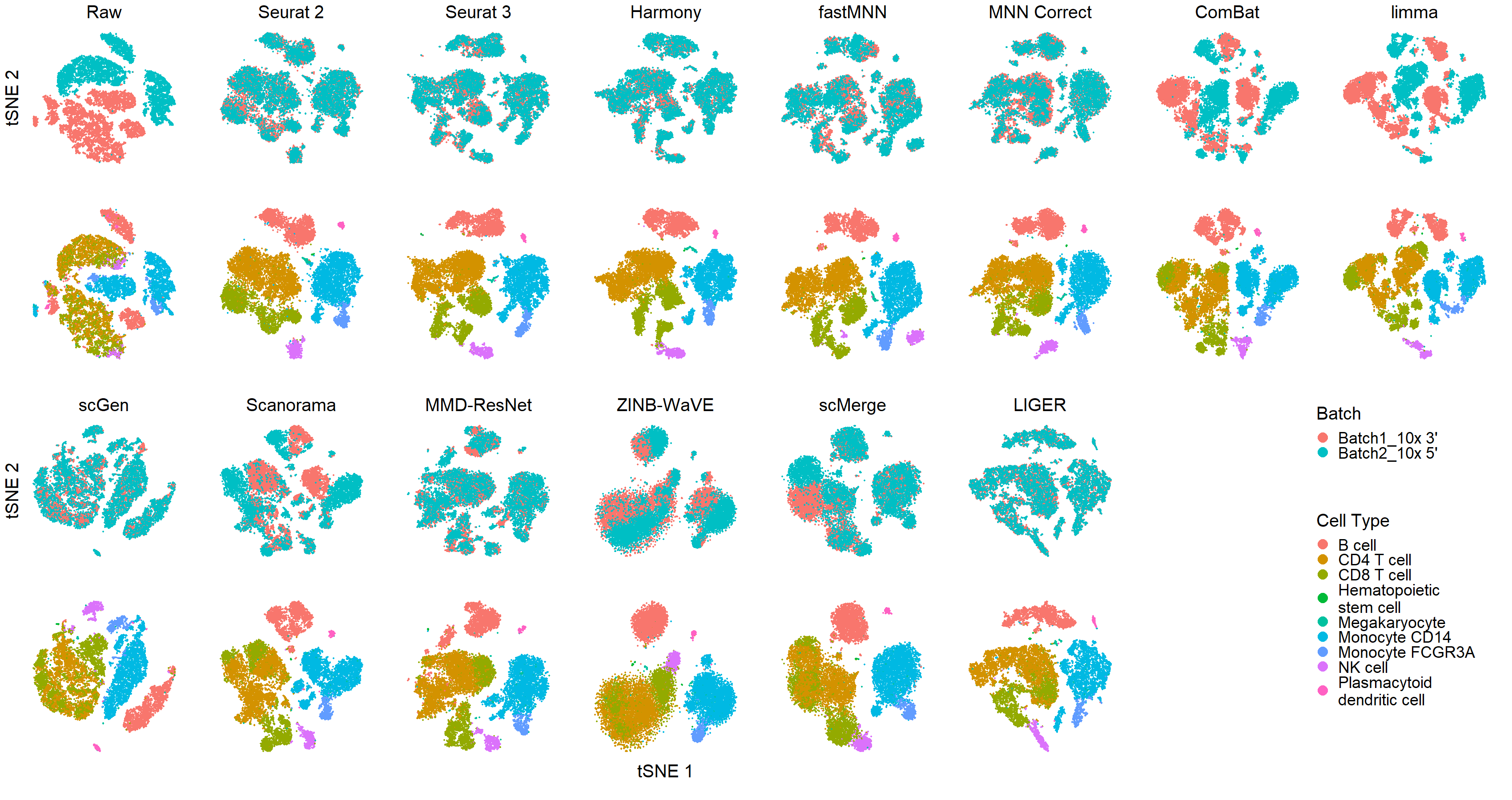
 **Fig S2: Qualitative evaluation of fourteen batch effect correction methods using tSNE visualization for Dataset 5 Human Peripheral Blood Mononuclear Cell.** The 14 methods are organized into two panels, the top panel showing t-SNE of raw data, Seurat 2, Seurat 3, Harmony, fastMNN, MNN Correct, ComBat, and limma, while the bottom panel shows t-SNE of scGen, Scanorama, MMD-ResNet, ZINB-WaVE, scMerge, and LIGER. Each panel contains two rows of t-SNE plots. In the first row cells are colored by batch and in the second by cell type.


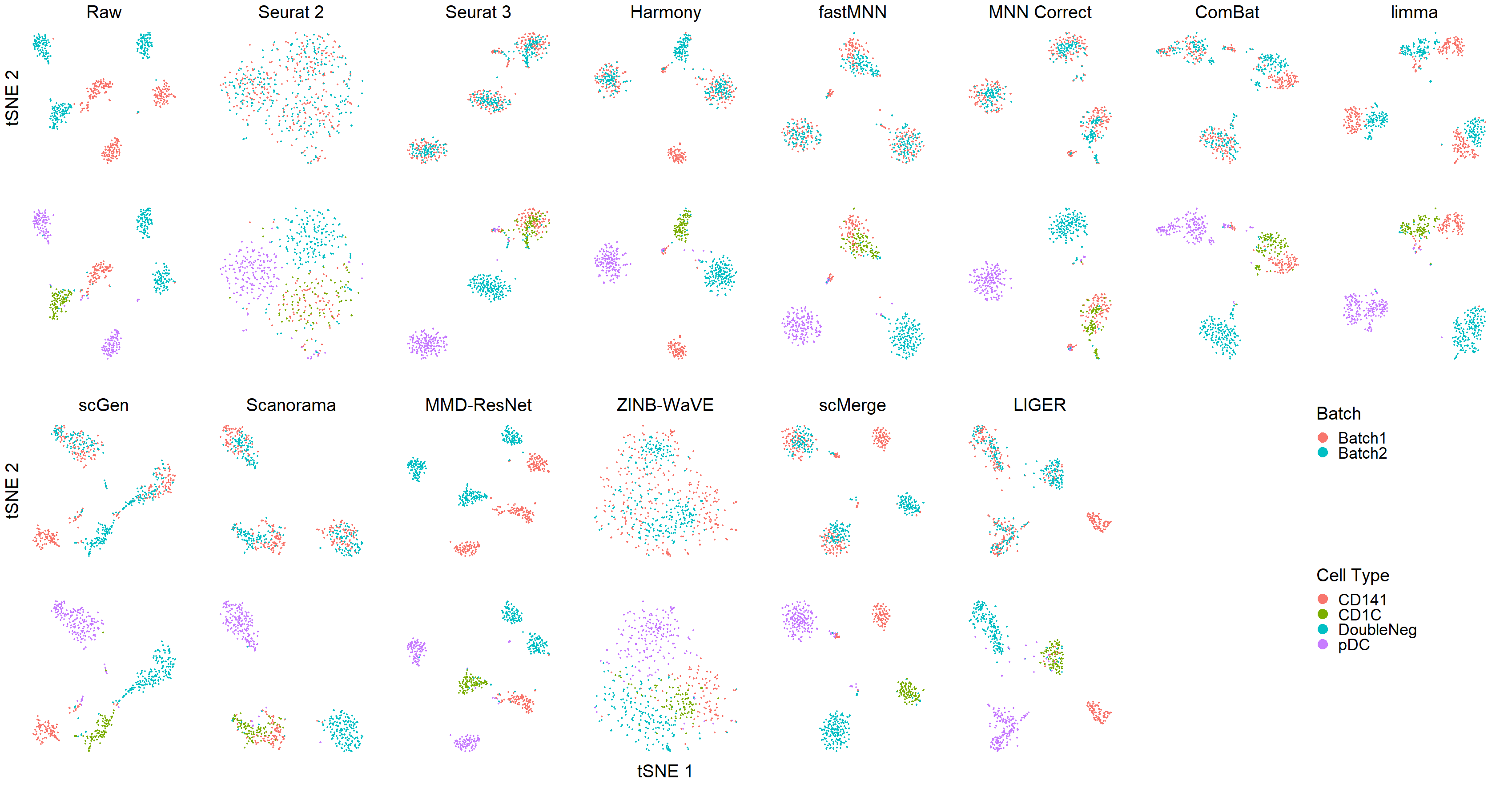
 **Fig S3: Qualitative evaluation of fourteen batch effect correction methods using tSNE visualization for Dataset 1 Human Dendritic Cells.** The 14 methods are organized into two panels, the top panel showing t-SNE of raw data, Seurat 2, Seurat 3, Harmony, fastMNN, MNN Correct, ComBat, and limma, while the bottom panel shows t-SNE of scGen, Scanorama, MMD-ResNet, ZINB-WaVE, scMerge, and LIGER. Each panel contains two rows of t-SNE plots. In the first row cells are colored by batch and in the second by cell type.


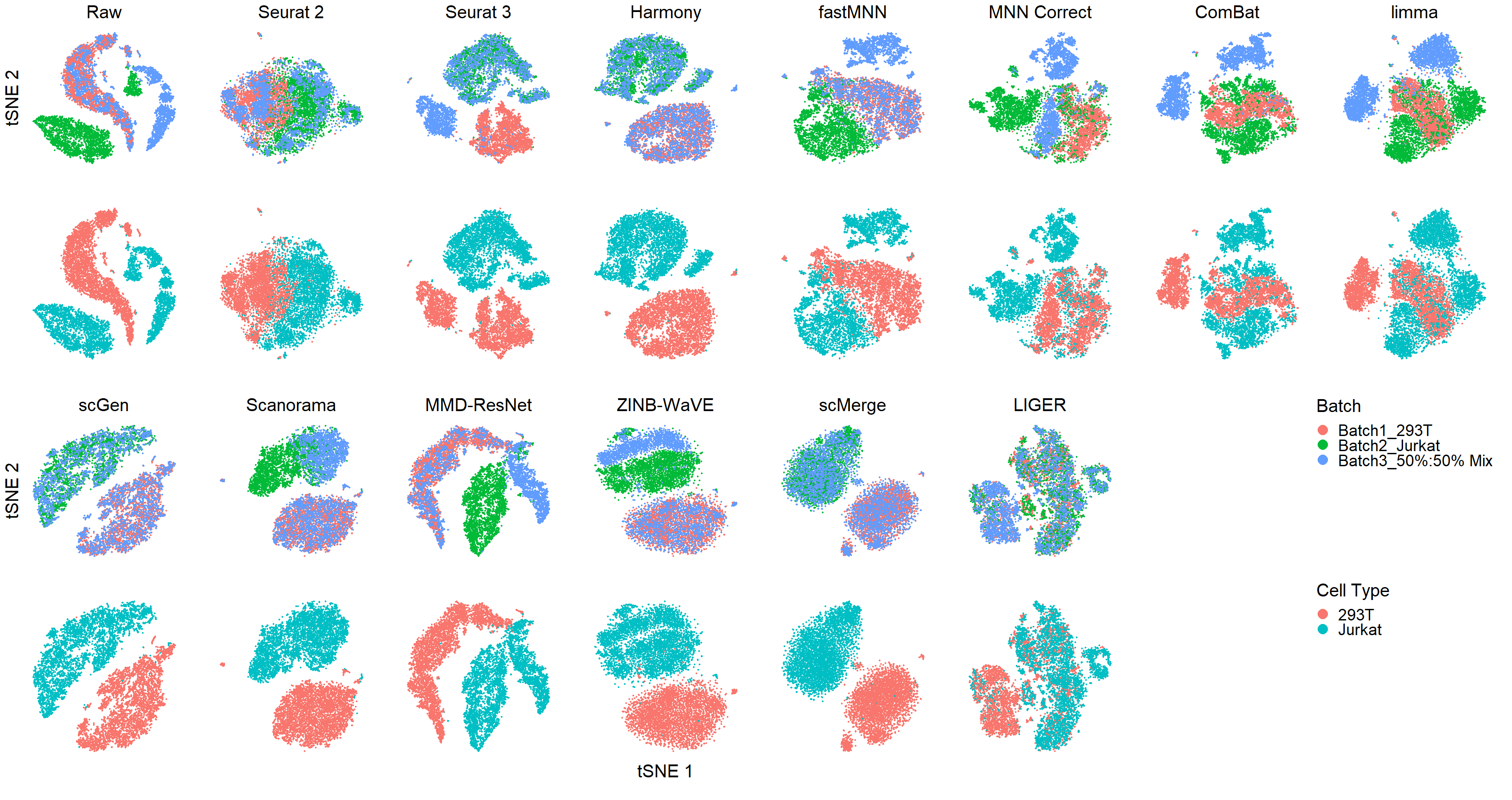
 **Fig S4: Qualitative evaluation of fourteen batch effect correction methods using tSNE visualization for Dataset 6 Cell Line.** The 14 methods are organized into two panels, the top panel showing t-SNE of raw data, Seurat 2, Seurat 3, Harmony, fastMNN, MNN Correct, ComBat, and limma, while the bottom panel shows t-SNE of scGen, Scanorama, MMD-ResNet, ZINB-WaVE, scMerge, and LIGER. Each panel contains two rows of t-SNE plots. In the first row cells are colored by batch and in the second by cell type.


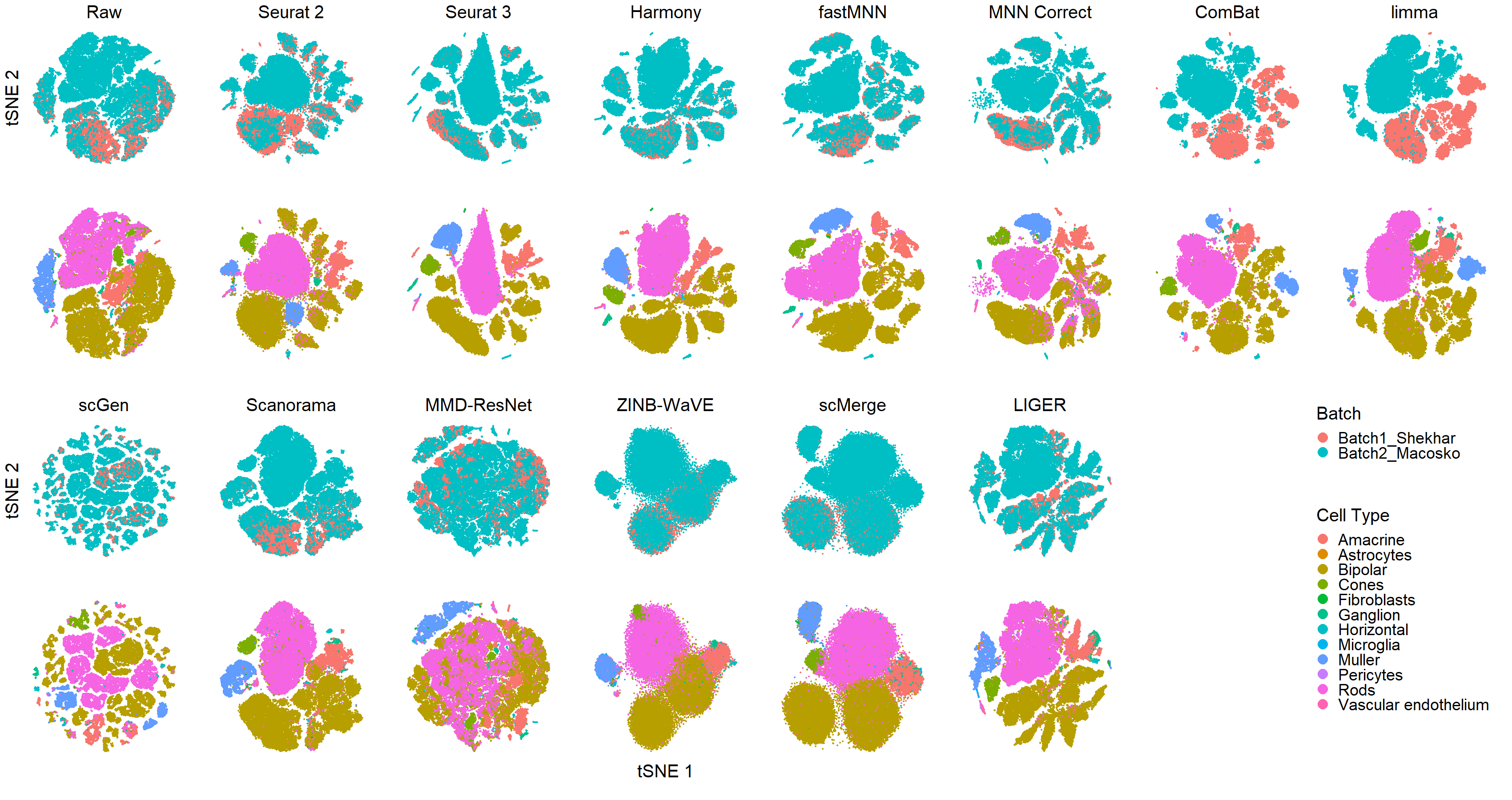
 **Fig S5: Qualitative evaluation of fourteen batch effect correction methods using tSNE visualization for Dataset 7 Mouse Retina.** The 14 methods are organized into two panels, the top panel showing t-SNE of raw data, Seurat 2, Seurat 3, Harmony, fastMNN, MNN Correct, ComBat, and limma, while the bottom panel shows t-SNE of scGen, Scanorama, MMD-ResNet, ZINB-WaVE, scMerge, and LIGER. Each panel contains two rows of t-SNE plots. In the first row cells are colored by batch and in the second by cell type.


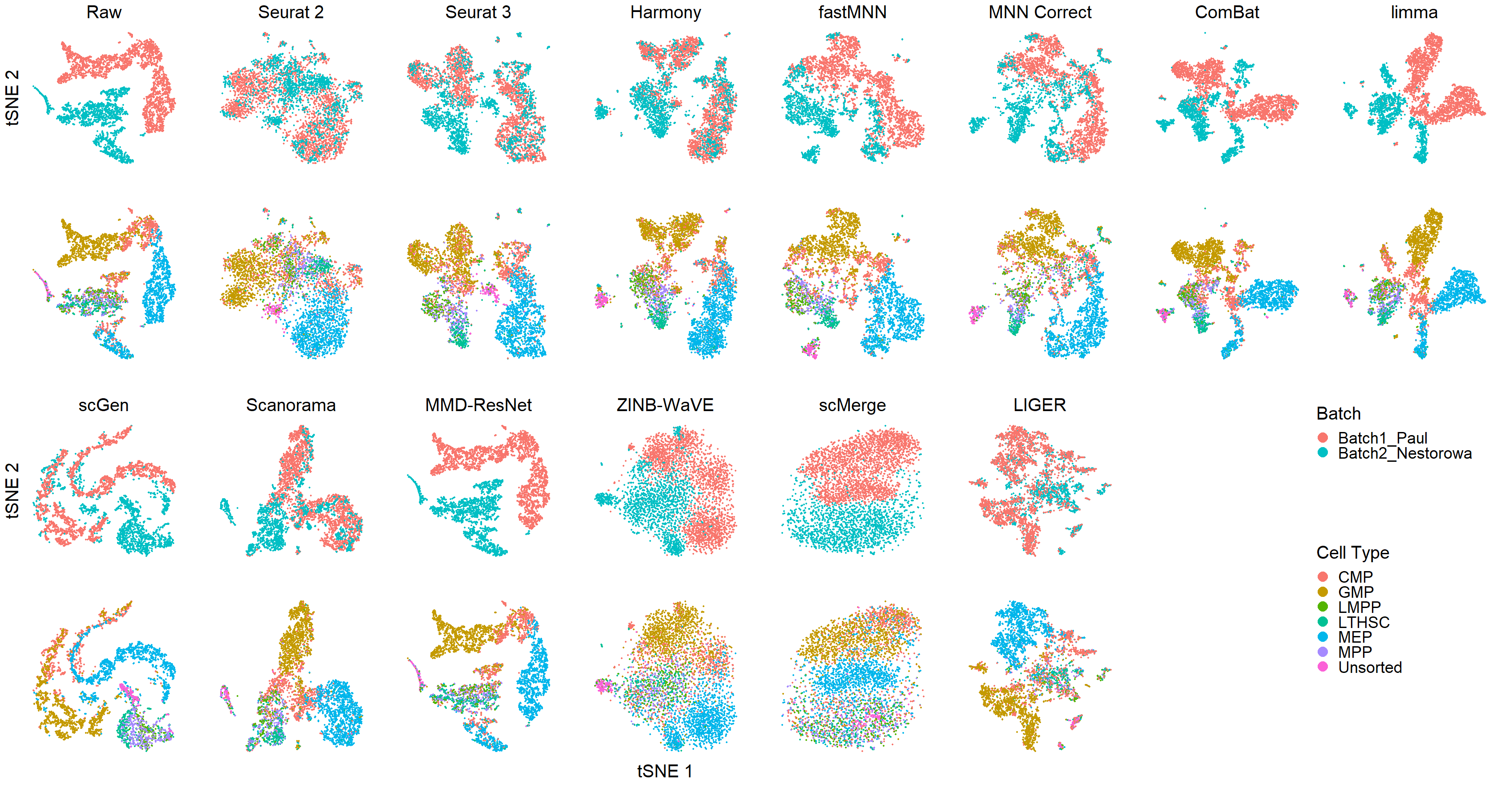
 **Fig S6: Qualitative evaluation of fourteen batch effect correction methods using tSNE visualization for Dataset 10 Mouse Haematopoietic Stem and Progenitor Cells.** The 14 methods are organized into two panels, the top panel showing t-SNE of raw data, Seurat 2, Seurat 3, Harmony, fastMNN, MNN Correct, ComBat, and limma, while the bottom panel shows t-SNE of scGen, Scanorama, MMD-ResNet, ZINB-WaVE, scMerge, and LIGER. Each panel contains two rows of t-SNE plots. In the first row cells are colored by batch and in the second by cell type.


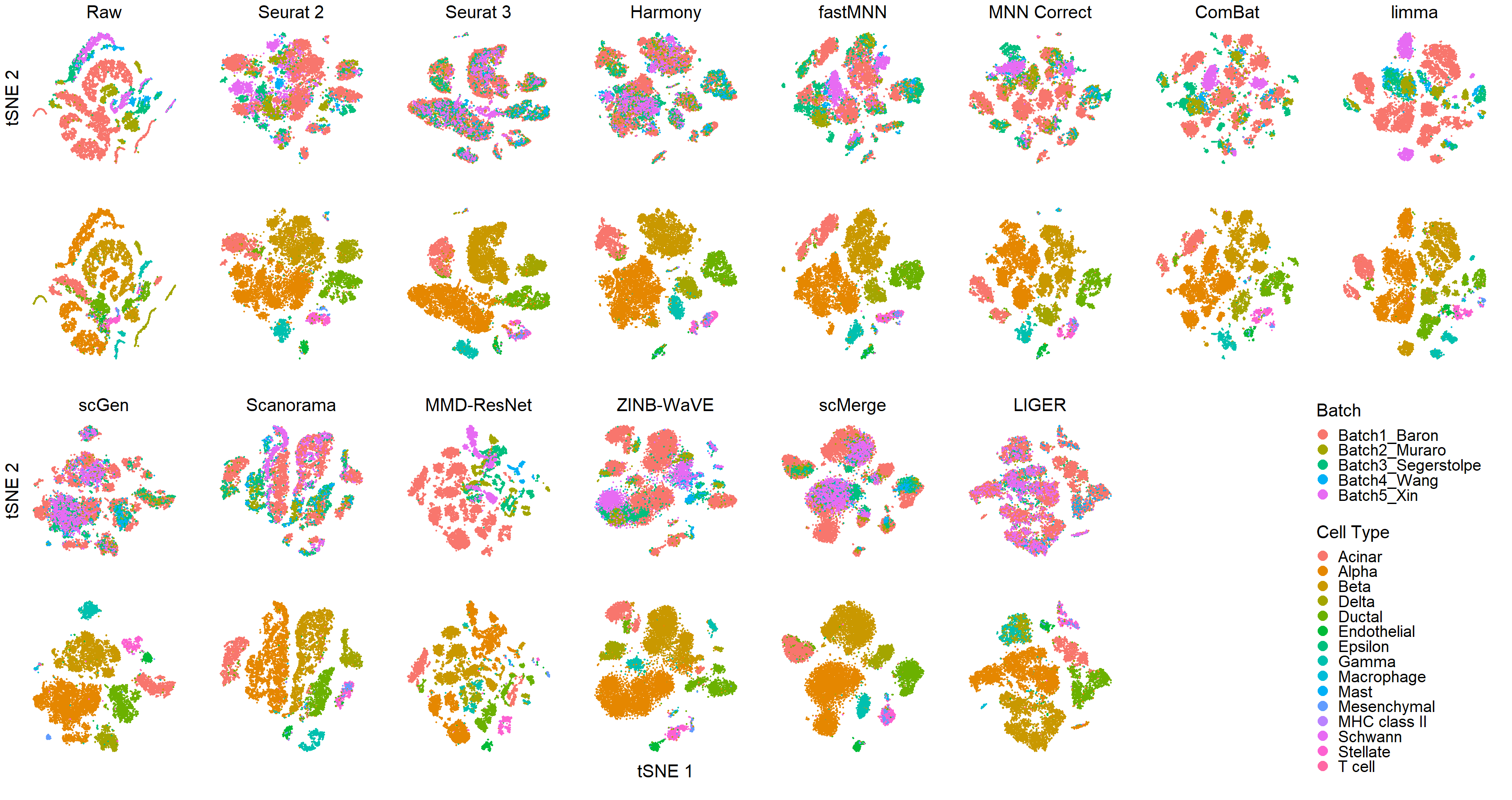
 **Fig S7: Qualitative evaluation of fourteen batch effect correction methods using tSNE visualization for Dataset 4 Human Pancreas.** The 14 methods are organized into two panels, the top panel showing t-SNE of raw data, Seurat 2, Seurat 3, Harmony, fastMNN, MNN Correct, ComBat, and limma, while the bottom panel shows t-SNE of scGen, Scanorama, MMD-ResNet, ZINB-WaVE, scMerge, and LIGER. Each panel contains two rows of t-SNE plots. In the first row cells are colored by batch and in the second by cell type.


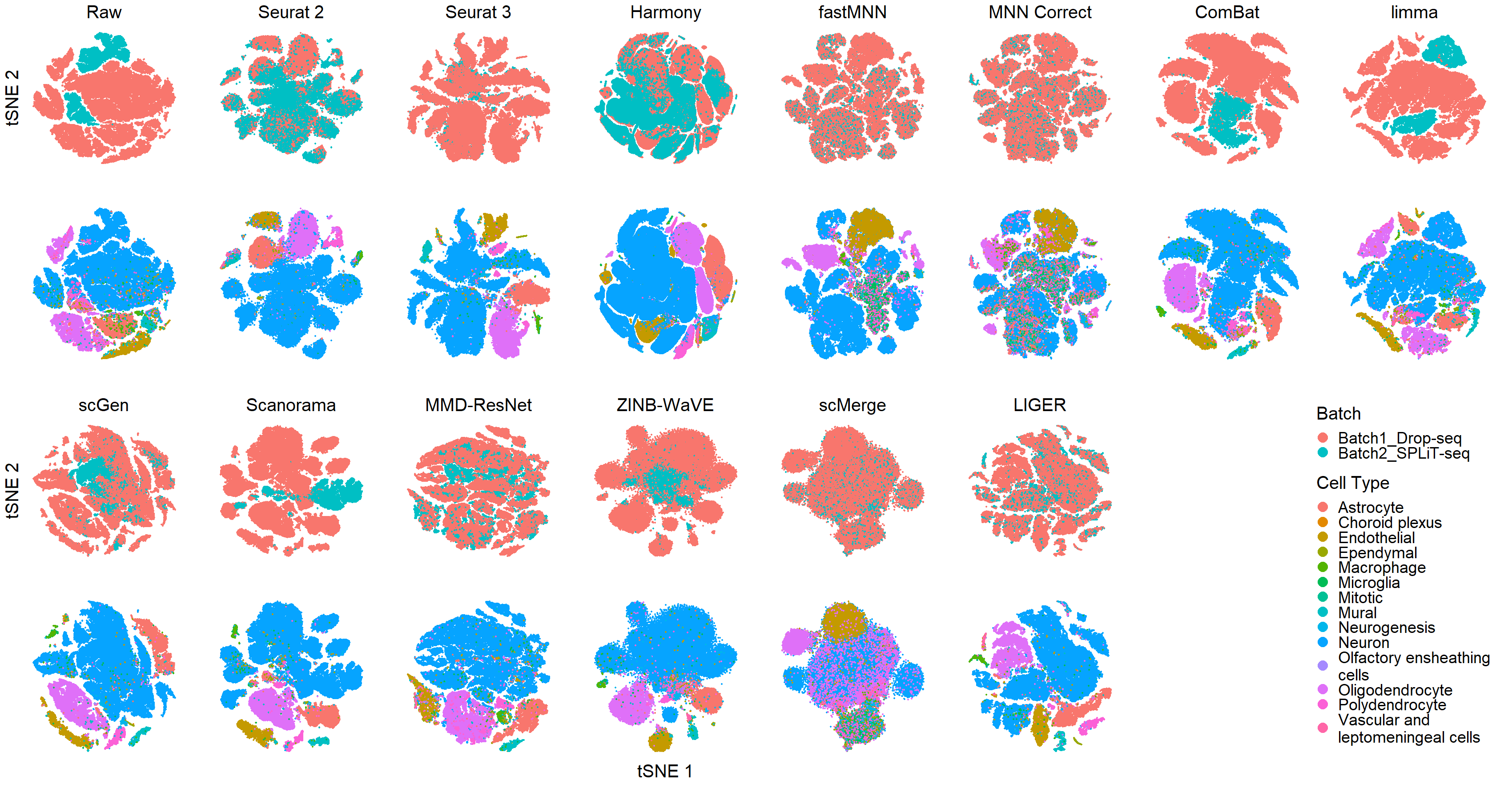
 **Fig S8: Qualitative evaluation of fourteen batch effect correction methods using tSNE visualization for Dataset 8 Mouse Brain.** The 14 methods are organized into two panels, the top panel showing t-SNE of raw data, Seurat 2, Seurat 3, Harmony, fastMNN, MNN Correct, ComBat, and limma, while the bottom panel shows t-SNE of scGen, Scanorama, MMD-ResNet, ZINB-WaVE, scMerge, and LIGER. Each panel contains two rows of t-SNE plots. In the first row cells are colored by batch and in the second by cell type.


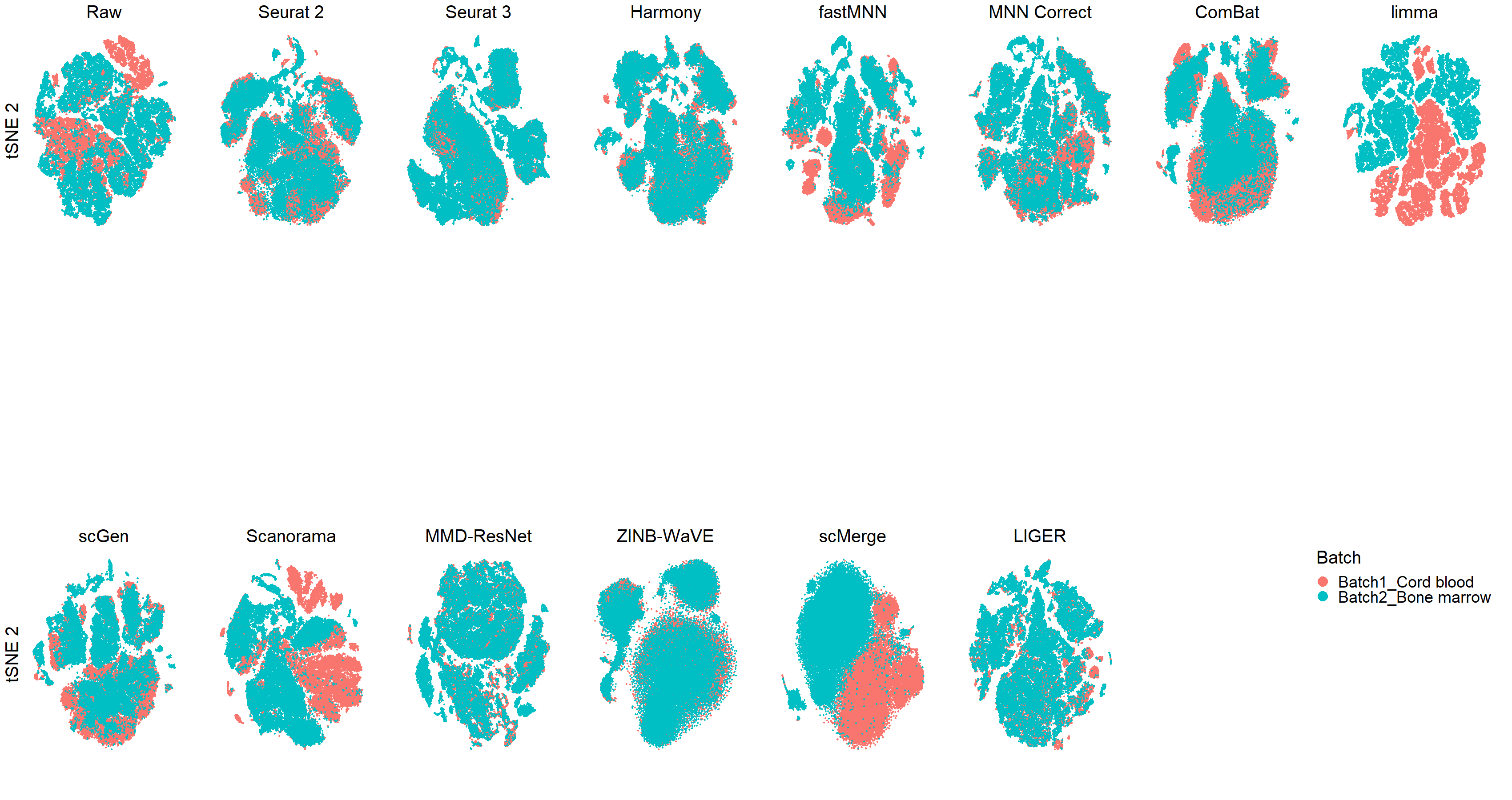
 **Fig S9: Qualitative evaluation of fourteen batch effect correction methods using tSNE visualization for Dataset 9 Human Cell Atlas.** The 14 methods are organized into two panels, the top panel showing t-SNE of raw data, Seurat 2, Seurat 3, Harmony, fastMNN, MNN Correct, ComBat, and limma, while the bottom panel shows t-SNE of scGen, Scanorama, MMD-ResNet, ZINB-WaVE, scMerge, and LIGER. Cells are colored by batch.


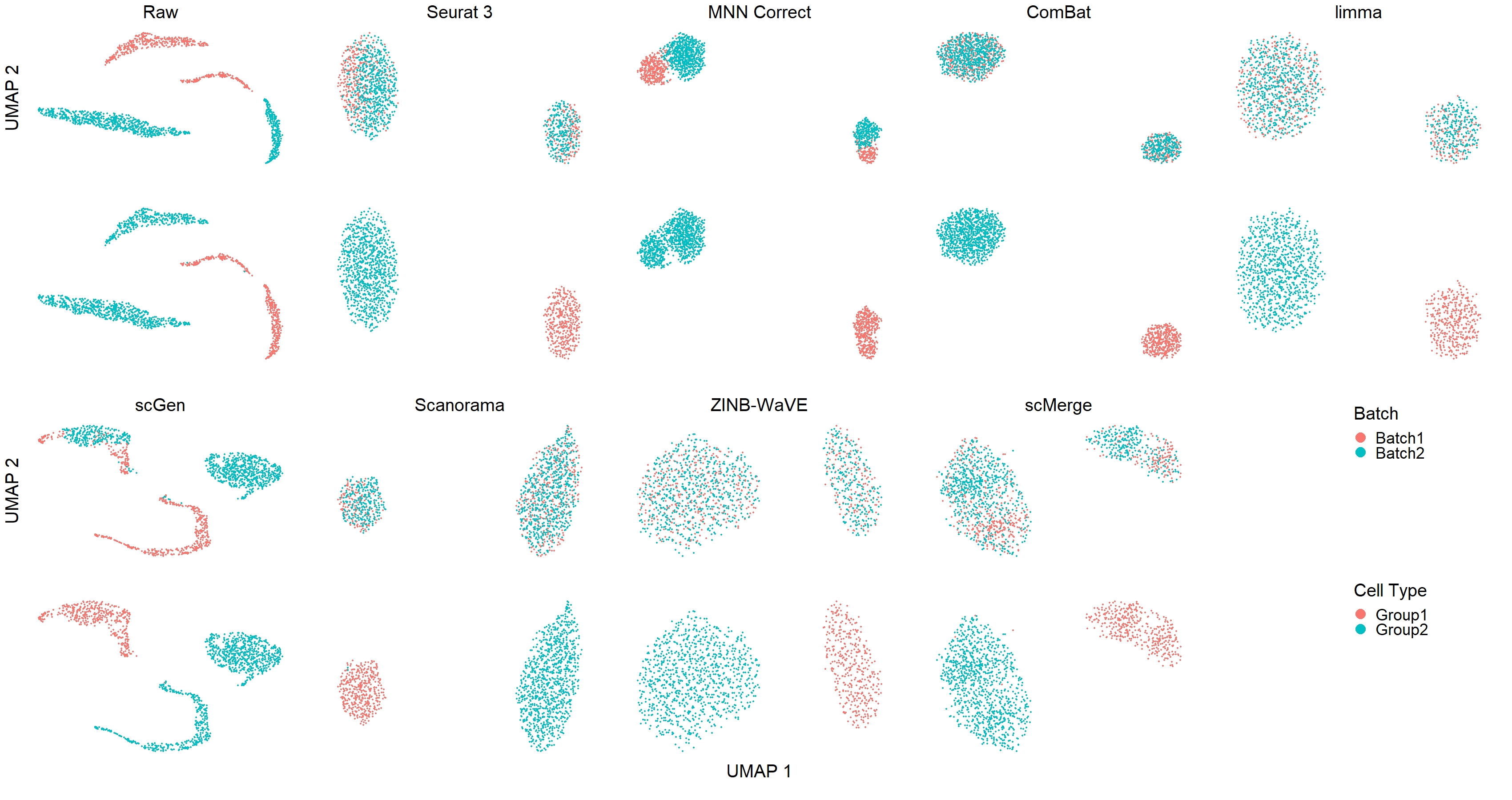
 **Fig S10: Qualitative evaluation of fourteen batch effect correction methods using UMAP visualization for Dataset 3 Simulation.** The 14 methods are organized into two panels, the top panel showing UMAP of raw data, Seurat 3, MNN Correct, ComBat, and limma, while the bottom panel shows UMAP of scGen, Scanorama, ZINB-WaVE, and scMerge. Cells are colored by batch.


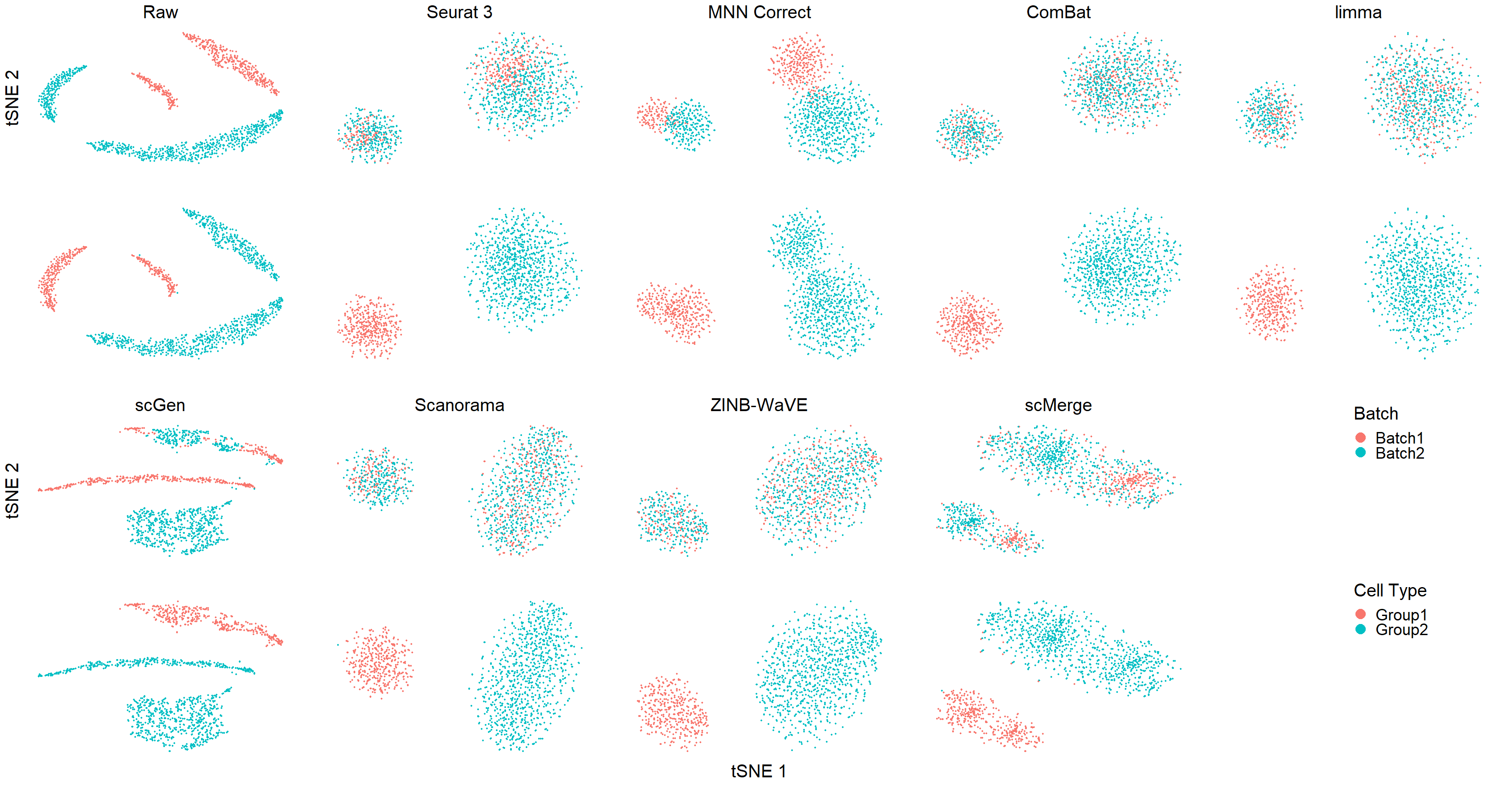
 **Fig S11: Qualitative evaluation of fourteen batch effect correction methods using tSNE visualization for Dataset 3 Simulation.** The 14 methods are organized into two panels, the top panel showing t-SNE of raw data, Seurat 3, MNN Correct, ComBat, and limma, while the bottom panel shows t-SNE of scGen, Scanorama, ZINB-WaVE, and scMerge. Cells are colored by batch.
