# Supplementary material for: A benchmark of batch-effect correction methods for single-cell RNA sequencing data
Source: Genome Biol. 2020 Jan 16;21:12. doi: 10.1186/s13059-019-1850-9 (PMC6964114; doi:10.1186/s13059-019-1850-9)
Supplement: Supplementary file 7 — Additional file 7: Table S6. Simulation statistical test. Statistical significance test results of the DEG recovery with simulated data. [file 13059_2019_1850_MOESM7_ESM.docx]

**Table S6: Simulation statistical test**

**A.**

| **Up-regulated in Group 1** | | **All genes** | | | | | |  | **HVG** | | | | | |
| --- | --- | --- | --- | --- | --- | --- | --- | --- | --- | --- | --- | --- | --- | --- |
|  |  | **TP** | **FN** | **FP** | **Recall** | **Precision** | **F-score** |  | **TP** | **FN** | **FP** | **Recall** | **Precision** | **F-score** |
| **Simulation 1** | Raw | 164 | 670 | 0 | 0.197 | 1 | 0.329 |  | 171 | 2 | 409 | 0.988 | 0.295 | 0.454 |
|  | Seurat 3 | 267 | 567 | 101 | 0.32 | 0.726 | 0.444 |  | 164 | 9 | 0 | 0.948 | 1 | 0.973 |
| dropout=0.05 | MNN Correct | 593 | 241 | 49 | 0.711 | 0.924 | 0.804 |  | 165 | 8 | 1 | 0.954 | 0.994 | 0.974 |
| b1=500 | ComBat | 600 | 234 | 69 | 0.719 | 0.897 | 0.798 |  | 161 | 12 | 0 | 0.931 | 1 | 0.964 |
| b2=900 | limma | 647 | 187 | 131 | 0.776 | 0.832 | 0.803 |  | 163 | 10 | 0 | 0.942 | 1 | 0.97 |
|  | scGen | 222 | 612 | 319 | 0.266 | 0.41 | 0.323 |  | 123 | 50 | 0 | 0.711 | 1 | 0.831 |
|  | Scanorama | 153 | 681 | 759 | 0.183 | 0.168 | 0.175 |  | 37 | 136 | 122 | 0.214 | 0.233 | 0.223 |
|  | ZINB-WaVE | 571 | 263 | 32 | 0.685 | 0.947 | 0.795 |  | 169 | 4 | 0 | 0.977 | 1 | 0.988 |
|  | scMerge | 601 | 233 | 68 | 0.721 | 0.898 | 0.8 |  | 162 | 11 | 0 | 0.936 | 1 | 0.967 |
| **Simulation 2** | Raw | 166 | 668 | 0 | 0.199 | 1 | 0.332 |  | 175 | 2 | 403 | 0.989 | 0.303 | 0.464 |
|  | Seurat 3 | 264 | 570 | 99 | 0.317 | 0.727 | 0.441 |  | 166 | 11 | 2 | 0.938 | 0.988 | 0.962 |
| dropout=0.25 | MNN Correct | 604 | 230 | 51 | 0.724 | 0.922 | 0.811 |  | 169 | 8 | 0 | 0.955 | 1 | 0.977 |
| b1=500 | ComBat | 606 | 228 | 78 | 0.727 | 0.886 | 0.799 |  | 163 | 14 | 0 | 0.921 | 1 | 0.959 |
| b2=900 | limma | 645 | 189 | 156 | 0.773 | 0.805 | 0.789 |  | 165 | 12 | 0 | 0.932 | 1 | 0.965 |
|  | scGen | 260 | 574 | 1066 | 0.312 | 0.196 | 0.241 |  | 169 | 8 | 0 | 0.955 | 1 | 0.977 |
|  | Scanorama | 150 | 684 | 767 | 0.18 | 0.164 | 0.172 |  | 43 | 134 | 119 | 0.243 | 0.265 | 0.254 |
|  | ZINB-WaVE | 578 | 256 | 30 | 0.693 | 0.951 | 0.802 |  | 172 | 5 | 1 | 0.972 | 0.994 | 0.983 |
|  | scMerge | 595 | 239 | 82 | 0.713 | 0.879 | 0.787 |  | 165 | 12 | 0 | 0.932 | 1 | 0.965 |
| **Simulation 3** | Raw | 147 | 677 | 0 | 0.178 | 1 | 0.302 |  | 152 | 13 | 332 | 0.921 | 0.314 | 0.468 |
|  | Seurat 3 | 234 | 590 | 90 | 0.284 | 0.722 | 0.408 |  | 142 | 23 | 1 | 0.861 | 0.993 | 0.922 |
| dropout=0.05 | MNN Correct | 511 | 313 | 47 | 0.62 | 0.916 | 0.739 |  | 147 | 18 | 1 | 0.891 | 0.993 | 0.939 |
| b1=500 | ComBat | 507 | 317 | 67 | 0.615 | 0.883 | 0.725 |  | 140 | 25 | 0 | 0.848 | 1 | 0.918 |
| b2=450 | limma | 592 | 232 | 141 | 0.718 | 0.808 | 0.76 |  | 147 | 18 | 0 | 0.891 | 1 | 0.942 |
|  | scGen | 386 | 438 | 1212 | 0.468 | 0.242 | 0.319 |  | 164 | 1 | 433 | 0.994 | 0.275 | 0.431 |
|  | Scanorama | 138 | 686 | 601 | 0.167 | 0.187 | 0.176 |  | 37 | 128 | 99 | 0.224 | 0.272 | 0.246 |
|  | ZINB-WaVE | 469 | 355 | 32 | 0.569 | 0.936 | 0.708 |  | 154 | 11 | 2 | 0.933 | 0.987 | 0.959 |
|  | scMerge | 487 | 337 | 71 | 0.591 | 0.873 | 0.705 |  | 137 | 28 | 0 | 0.83 | 1 | 0.907 |
| **Simulation 4** | Raw | 146 | 678 | 0 | 0.177 | 1 | 0.301 |  | 155 | 10 | 328 | 0.939 | 0.321 | 0.478 |
|  | Seurat 3 | 244 | 580 | 83 | 0.296 | 0.746 | 0.424 |  | 141 | 24 | 1 | 0.855 | 0.993 | 0.919 |
| dropout=0.25 | MNN Correct | 505 | 319 | 56 | 0.613 | 0.9 | 0.729 |  | 146 | 19 | 0 | 0.885 | 1 | 0.939 |
| b1=500 | ComBat | 492 | 332 | 83 | 0.597 | 0.856 | 0.703 |  | 135 | 30 | 0 | 0.818 | 1 | 0.9 |
| b2=450 | limma | 598 | 226 | 160 | 0.726 | 0.789 | 0.756 |  | 146 | 19 | 0 | 0.885 | 1 | 0.939 |
|  | scGen | 86 | 738 | 160 | 0.104 | 0.35 | 0.16 |  | 152 | 13 | 7 | 0.921 | 0.956 | 0.938 |
|  | Scanorama | 142 | 682 | 614 | 0.172 | 0.188 | 0.18 |  | 28 | 137 | 107 | 0.17 | 0.207 | 0.187 |
|  | ZINB-WaVE | 470 | 354 | 27 | 0.57 | 0.946 | 0.711 |  | 155 | 10 | 3 | 0.939 | 0.981 | 0.96 |
|  | scMerge | 480 | 344 | 83 | 0.583 | 0.853 | 0.693 |  | 134 | 31 | 0 | 0.812 | 1 | 0.896 |
| **Simulation 5** | Raw | 121 | 715 | 0 | 0.145 | 1 | 0.253 |  | 136 | 16 | 237 | 0.895 | 0.365 | 0.519 |
|  | Seurat 3 | 157 | 679 | 38 | 0.188 | 0.805 | 0.305 |  | 122 | 30 | 0 | 0.803 | 1 | 0.891 |
| dropout=0.05 | MNN Correct | 401 | 435 | 57 | 0.48 | 0.876 | 0.62 |  | 131 | 21 | 1 | 0.862 | 0.992 | 0.922 |
| b1=80 | ComBat | 366 | 470 | 69 | 0.438 | 0.841 | 0.576 |  | 112 | 40 | 0 | 0.737 | 1 | 0.849 |
| b2=400 | limma | 452 | 384 | 115 | 0.541 | 0.797 | 0.645 |  | 121 | 31 | 0 | 0.796 | 1 | 0.886 |
|  | scGen | 416 | 420 | 1914 | 0.498 | 0.179 | 0.263 |  | 145 | 7 | 6 | 0.954 | 0.96 | 0.957 |
|  | Scanorama | 74 | 762 | 365 | 0.089 | 0.169 | 0.117 |  | 26 | 126 | 59 | 0.171 | 0.306 | 0.219 |
|  | ZINB-WaVE | 393 | 443 | 24 | 0.47 | 0.942 | 0.627 |  | 141 | 11 | 2 | 0.928 | 0.986 | 0.956 |
|  | scMerge | 349 | 487 | 60 | 0.417 | 0.853 | 0.56 |  | 108 | 44 | 0 | 0.711 | 1 | 0.831 |
| **Simulation 6** | Raw | 123 | 713 | 0 | 0.147 | 1 | 0.256 |  | 137 | 19 | 217 | 0.878 | 0.387 | 0.537 |
|  | Seurat 3 | 161 | 675 | 42 | 0.193 | 0.793 | 0.31 |  | 121 | 35 | 0 | 0.776 | 1 | 0.874 |
| dropout=0.25 | MNN Correct | 387 | 449 | 78 | 0.463 | 0.832 | 0.595 |  | 133 | 23 | 1 | 0.853 | 0.993 | 0.918 |
| b1=80 | ComBat | 372 | 464 | 93 | 0.445 | 0.8 | 0.572 |  | 111 | 45 | 0 | 0.712 | 1 | 0.832 |
| b2=400 | limma | 439 | 397 | 120 | 0.525 | 0.785 | 0.629 |  | 122 | 34 | 0 | 0.782 | 1 | 0.878 |
|  | scGen | 246 | 590 | 15 | 0.294 | 0.943 | 0.448 |  | 77 | 79 | 0 | 0.494 | 1 | 0.661 |
|  | Scanorama | 68 | 768 | 354 | 0.081 | 0.161 | 0.108 |  | 18 | 138 | 66 | 0.115 | 0.214 | 0.15 |
|  | ZINB-WaVE | 372 | 464 | 21 | 0.445 | 0.947 | 0.605 |  | 142 | 14 | 2 | 0.91 | 0.986 | 0.946 |
|  | scMerge | 341 | 495 | 67 | 0.408 | 0.836 | 0.548 |  | 104 | 52 | 0 | 0.667 | 1 | 0.8 |

**B.**

| **Up-regulated in Group 1** | | Raw | | Seurat 3 | | MNN Correct | | ComBat | | limma | | scGen | | Scanorama | | ZINB-WaVE | |
| --- | --- | --- | --- | --- | --- | --- | --- | --- | --- | --- | --- | --- | --- | --- | --- | --- | --- |
| **All genes** | Seurat 3 | 0.039 | * |  |  |  |  |  |  |  |  |  |  |  |  |  |  |
|  | MNN Correct | 0.004 | ** | 0.004 | ** |  |  |  |  |  |  |  |  |  |  |  |  |
|  | ComBat | 0.004 | ** | 0.004 | ** | 0.525 | NS |  |  |  |  |  |  |  |  |  |  |
|  | limma | 0.004 | ** | 0.004 | ** | 0.818 | NS | 0.684 | NS |  |  |  |  |  |  |  |  |
|  | scGen | 0.818 | NS | 0.346 | NS | 0.004 | ** | 0.004 | ** | 0.004 | ** |  |  |  |  |  |  |
|  | Scanorama | 0.004 | ** | 0.004 | ** | 0.004 | ** | 0.004 | ** | 0.004 | ** | 0.039 | * |  |  |  |  |
|  | ZINB-WaVE | 0.004 | ** | 0.004 | ** | 0.763 | NS | 0.818 | NS | 0.684 | NS | 0.004 | ** | 0.004 | ** |  |  |
|  | scMerge | 0.004 | ** | 0.004 | ** | 0.525 | NS | 0.763 | NS | 0.602 | NS | 0.004 | ** | 0.004 | ** | 0.602 | NS |
|  |  |  |  |  |  |  |  |  |  |  |  |  |  |  |  |  |  |
| **HVG** | Seurat 3 | 0.006 | ** |  |  |  |  |  |  |  |  |  |  |  |  |  |  |
|  | MNN Correct | 0.013 | * | 0.391 | NS |  |  |  |  |  |  |  |  |  |  |  |  |
|  | ComBat | 0.006 | ** | 0.623 | NS | 0.232 | NS |  |  |  |  |  |  |  |  |  |  |
|  | limma | 0.006 | ** | 0.842 | NS | 0.749 | NS | 0.525 | NS |  |  |  |  |  |  |  |  |
|  | scGen | 0.146 | NS | 0.662 | NS | 0.482 | NS | 0.662 | NS | 0.525 | NS |  |  |  |  |  |  |
|  | Scanorama | 0.006 | ** | 0.006 | ** | 0.013 | * | 0.006 | ** | 0.006 | ** | 0.006 | ** |  |  |  |  |
|  | ZINB-WaVE | 0.006 | ** | 0.216 | NS | 0.216 | NS | 0.164 | NS | 0.216 | NS | 0.146 | NS | 0.006 | ** |  |  |
|  | scMerge | 0.006 | ** | 0.662 | NS | 0.216 | NS | 0.937 | NS | 0.647 | NS | 0.792 | NS | 0.006 | ** | 0.216 | NS |

**C.**

| **Up-regulated in Group 1** | **All genes** | | | | |  | **HVG** | | | | |
| --- | --- | --- | --- | --- | --- | --- | --- | --- | --- | --- | --- |
|  | **min** | **Q1** | **median** | **Q3** | **max** |  | **min** | **Q1** | **median** | **Q3** | **max** |
| Raw | 0.253 | 0.26725 | 0.3 | 0.32225 | 0.332 |  | 0.454 | 0.465 | 0.47 | 0.50875 | 0.537 |
| Seurat 3 | 0.305 | 0.3345 | 0.42 | 0.43675 | 0.444 |  | 0.874 | 0.898 | 0.92 | 0.952 | 0.973 |
| MNN Correct | 0.595 | 0.64725 | 0.73 | 0.78775 | 0.811 |  | 0.918 | 0.92625 | 0.94 | 0.96525 | 0.977 |
| ComBat | 0.572 | 0.60775 | 0.71 | 0.77975 | 0.799 |  | 0.832 | 0.86175 | 0.91 | 0.94875 | 0.964 |
| limma | 0.629 | 0.67275 | 0.76 | 0.78175 | 0.803 |  | 0.878 | 0.89925 | 0.94 | 0.95925 | 0.97 |
| scGen | 0.16 | 0.2465 | 0.29 | 0.322 | 0.448 |  | 0.431 | 0.7035 | 0.88 | 0.95225 | 0.977 |
| Scanorama | 0.108 | 0.13075 | 0.17 | 0.17575 | 0.18 |  | 0.15 | 0.195 | 0.22 | 0.24025 | 0.254 |
| ZINB-WaVE | 0.605 | 0.64725 | 0.71 | 0.774 | 0.802 |  | 0.946 | 0.95675 | 0.96 | 0.97725 | 0.988 |
| scMerge | 0.548 | 0.59325 | 0.7 | 0.7665 | 0.8 |  | 0.8 | 0.84725 | 0.9 | 0.9505 | 0.967 |

**D.**

| **Down-regulated in Group 1** | | **All genes** | | | | | |  | **HVG** | | | | | |
| --- | --- | --- | --- | --- | --- | --- | --- | --- | --- | --- | --- | --- | --- | --- |
|  |  | **TP** | **FN** | **FP** | **Recall** | **Precision** | **F-score** |  | **TP** | **FN** | **FP** | **Recall** | **Precision** | **F-score** |
| **Simulation 1** | Raw | 100 | 458 | 204 | 0.179 | 0.329 | 0.232 |  | 100 | 0 | 298 | 1 | 0.251 | 0.401 |
|  | Seurat 3 | 162 | 396 | 156 | 0.29 | 0.509 | 0.369 |  | 100 | 0 | 126 | 1 | 0.442 | 0.613 |
| dropout=0.05 | MNN Correct | 401 | 157 | 151 | 0.719 | 0.726 | 0.722 |  | 99 | 1 | 51 | 0.99 | 0.66 | 0.792 |
| b1=500 | ComBat | 406 | 152 | 243 | 0.728 | 0.626 | 0.673 |  | 100 | 0 | 58 | 1 | 0.633 | 0.775 |
| b2=900 | limma | 436 | 122 | 260 | 0.781 | 0.626 | 0.695 |  | 100 | 0 | 190 | 1 | 0.345 | 0.513 |
|  | scGen | 265 | 293 | 904 | 0.475 | 0.227 | 0.307 |  | 100 | 0 | 453 | 1 | 0.181 | 0.307 |
|  | Scanorama | 70 | 488 | 596 | 0.125 | 0.105 | 0.114 |  | 63 | 37 | 402 | 0.63 | 0.135 | 0.222 |
|  | ZINB-WaVE | 372 | 186 | 129 | 0.667 | 0.743 | 0.703 |  | 100 | 0 | 71 | 1 | 0.585 | 0.738 |
|  | scMerge | 408 | 150 | 226 | 0.731 | 0.644 | 0.685 |  | 98 | 2 | 54 | 0.98 | 0.645 | 0.778 |
| **Simulation 2** | Raw | 101 | 457 | 207 | 0.181 | 0.328 | 0.233 |  | 101 | 0 | 289 | 1 | 0.259 | 0.411 |
|  | Seurat 3 | 160 | 398 | 165 | 0.287 | 0.492 | 0.363 |  | 101 | 0 | 120 | 1 | 0.457 | 0.627 |
| dropout=0.25 | MNN Correct | 399 | 159 | 150 | 0.715 | 0.727 | 0.721 |  | 100 | 1 | 34 | 0.99 | 0.746 | 0.851 |
| b1=500 | ComBat | 409 | 149 | 250 | 0.733 | 0.621 | 0.672 |  | 100 | 1 | 34 | 0.99 | 0.746 | 0.851 |
| b2=900 | limma | 434 | 124 | 289 | 0.778 | 0.6 | 0.678 |  | 101 | 0 | 191 | 1 | 0.346 | 0.514 |
|  | scGen | 35 | 523 | 311 | 0.063 | 0.101 | 0.078 |  | 101 | 0 | 395 | 1 | 0.204 | 0.339 |
|  | Scanorama | 74 | 484 | 616 | 0.133 | 0.107 | 0.119 |  | 60 | 41 | 434 | 0.594 | 0.121 | 0.201 |
|  | ZINB-WaVE | 365 | 193 | 126 | 0.654 | 0.743 | 0.696 |  | 101 | 0 | 76 | 1 | 0.571 | 0.727 |
|  | scMerge | 391 | 167 | 208 | 0.701 | 0.653 | 0.676 |  | 98 | 3 | 36 | 0.97 | 0.731 | 0.834 |
| **Simulation 3** | Raw | 111 | 407 | 60 | 0.214 | 0.649 | 0.322 |  | 107 | 7 | 238 | 0.939 | 0.31 | 0.466 |
|  | Seurat 3 | 176 | 342 | 170 | 0.34 | 0.509 | 0.408 |  | 109 | 5 | 65 | 0.956 | 0.626 | 0.757 |
| dropout=0.05 | MNN Correct | 351 | 167 | 118 | 0.678 | 0.748 | 0.711 |  | 108 | 6 | 10 | 0.947 | 0.915 | 0.931 |
| b1=500 | ComBat | 377 | 141 | 261 | 0.728 | 0.591 | 0.652 |  | 110 | 4 | 38 | 0.965 | 0.743 | 0.84 |
| b2=450 | limma | 400 | 118 | 248 | 0.772 | 0.617 | 0.686 |  | 111 | 3 | 64 | 0.974 | 0.634 | 0.768 |
|  | scGen | 268 | 250 | 722 | 0.517 | 0.271 | 0.356 |  | 79 | 35 | 16 | 0.693 | 0.832 | 0.756 |
|  | Scanorama | 91 | 427 | 707 | 0.176 | 0.114 | 0.138 |  | 77 | 37 | 381 | 0.675 | 0.168 | 0.269 |
|  | ZINB-WaVE | 337 | 181 | 98 | 0.651 | 0.775 | 0.708 |  | 108 | 6 | 17 | 0.947 | 0.864 | 0.904 |
|  | scMerge | 375 | 143 | 200 | 0.724 | 0.652 | 0.686 |  | 108 | 6 | 27 | 0.947 | 0.8 | 0.867 |
| **Simulation 4** | Raw | 113 | 405 | 67 | 0.218 | 0.628 | 0.324 |  | 110 | 6 | 229 | 0.948 | 0.324 | 0.483 |
|  | Seurat 3 | 177 | 341 | 174 | 0.342 | 0.504 | 0.407 |  | 113 | 3 | 69 | 0.974 | 0.621 | 0.758 |
| dropout=0.25 | MNN Correct | 356 | 162 | 111 | 0.687 | 0.762 | 0.723 |  | 112 | 4 | 12 | 0.966 | 0.903 | 0.933 |
| b1=500 | ComBat | 375 | 143 | 247 | 0.724 | 0.603 | 0.658 |  | 110 | 6 | 37 | 0.948 | 0.748 | 0.836 |
| b2=450 | limma | 403 | 115 | 249 | 0.778 | 0.618 | 0.689 |  | 113 | 3 | 63 | 0.974 | 0.642 | 0.774 |
|  | scGen | 346 | 172 | 2763 | 0.668 | 0.111 | 0.19 |  | 115 | 1 | 194 | 0.991 | 0.372 | 0.541 |
|  | Scanorama | 80 | 438 | 729 | 0.154 | 0.099 | 0.121 |  | 78 | 38 | 420 | 0.672 | 0.157 | 0.255 |
|  | ZINB-WaVE | 335 | 183 | 111 | 0.647 | 0.751 | 0.695 |  | 111 | 5 | 15 | 0.957 | 0.881 | 0.917 |
|  | scMerge | 361 | 157 | 207 | 0.697 | 0.636 | 0.665 |  | 112 | 4 | 22 | 0.966 | 0.836 | 0.896 |
| **Simulation 5** | Raw | 106 | 432 | 171 | 0.197 | 0.383 | 0.26 |  | 106 | 1 | 196 | 0.991 | 0.351 | 0.518 |
|  | Seurat 3 | 123 | 415 | 85 | 0.229 | 0.591 | 0.33 |  | 107 | 0 | 80 | 1 | 0.572 | 0.728 |
| dropout=0.05 | MNN Correct | 285 | 253 | 119 | 0.53 | 0.705 | 0.605 |  | 105 | 2 | 7 | 0.981 | 0.938 | 0.959 |
| b1=80 | ComBat | 294 | 244 | 276 | 0.546 | 0.516 | 0.531 |  | 105 | 2 | 41 | 0.981 | 0.719 | 0.83 |
| b2=400 | limma | 352 | 186 | 271 | 0.654 | 0.565 | 0.606 |  | 106 | 1 | 157 | 0.991 | 0.403 | 0.573 |
|  | scGen | 31 | 507 | 92 | 0.058 | 0.252 | 0.094 |  | 107 | 0 | 259 | 1 | 0.292 | 0.452 |
|  | Scanorama | 81 | 457 | 612 | 0.151 | 0.117 | 0.132 |  | 81 | 26 | 409 | 0.757 | 0.165 | 0.271 |
|  | ZINB-WaVE | 252 | 286 | 52 | 0.468 | 0.829 | 0.598 |  | 105 | 2 | 17 | 0.981 | 0.861 | 0.917 |
|  | scMerge | 276 | 262 | 247 | 0.513 | 0.528 | 0.52 |  | 103 | 4 | 27 | 0.963 | 0.792 | 0.869 |
| **Simulation 6** | Raw | 110 | 428 | 154 | 0.204 | 0.417 | 0.274 |  | 109 | 2 | 180 | 0.982 | 0.377 | 0.545 |
|  | Seurat 3 | 112 | 426 | 102 | 0.208 | 0.523 | 0.298 |  | 111 | 0 | 59 | 1 | 0.653 | 0.79 |
| dropout=0.25 | MNN Correct | 296 | 242 | 130 | 0.55 | 0.695 | 0.614 |  | 108 | 3 | 5 | 0.973 | 0.956 | 0.964 |
| b1=80 | ComBat | 294 | 244 | 328 | 0.546 | 0.473 | 0.507 |  | 109 | 2 | 26 | 0.982 | 0.807 | 0.886 |
| b2=400 | limma | 352 | 186 | 315 | 0.654 | 0.528 | 0.584 |  | 110 | 1 | 138 | 0.991 | 0.444 | 0.613 |
|  | scGen | 270 | 268 | 1926 | 0.502 | 0.123 | 0.198 |  | 111 | 0 | 437 | 1 | 0.203 | 0.337 |
|  | Scanorama | 77 | 461 | 625 | 0.143 | 0.11 | 0.124 |  | 79 | 32 | 427 | 0.712 | 0.156 | 0.256 |
|  | ZINB-WaVE | 249 | 289 | 61 | 0.463 | 0.803 | 0.587 |  | 109 | 2 | 14 | 0.982 | 0.886 | 0.932 |
|  | scMerge | 275 | 263 | 255 | 0.511 | 0.519 | 0.515 |  | 107 | 4 | 12 | 0.964 | 0.899 | 0.93 |

**E.**

| **Down-regulated in Group 1** | | Raw | | Seurat 3 | | MNN Correct | | ComBat | | limma | | scGen | | Scanorama | | ZINB-WaVE | |
| --- | --- | --- | --- | --- | --- | --- | --- | --- | --- | --- | --- | --- | --- | --- | --- | --- | --- |
| **All genes** | Seurat 3 | 0.014 | * |  |  |  |  |  |  |  |  |  |  |  |  |  |  |
|  | MNN Correct | 0.004 | ** | 0.004 | ** |  |  |  |  |  |  |  |  |  |  |  |  |
|  | ComBat | 0.004 | ** | 0.004 | ** | 0.158 | NS |  |  |  |  |  |  |  |  |  |  |
|  | limma | 0.004 | ** | 0.004 | ** | 0.209 | NS | 0.158 | NS |  |  |  |  |  |  |  |  |
|  | scGen | 0.27 | NS | 0.023 | * | 0.004 | ** | 0.004 | ** | 0.004 | ** |  |  |  |  |  |  |
|  | Scanorama | 0.004 | ** | 0.004 | ** | 0.004 | ** | 0.004 | ** | 0.004 | ** | 0.394 | NS |  |  |  |  |
|  | ZINB-WaVE | 0.004 | ** | 0.004 | ** | 0.158 | NS | 0.158 | NS | 0.277 | NS | 0.004 | ** | 0.004 | ** |  |  |
|  | scMerge | 0.004 | ** | 0.004 | ** | 0.158 | NS | 0.394 | NS | 0.277 | NS | 0.004 | ** | 0.004 | ** | 0.158 | NS |
|  |  |  |  |  |  |  |  |  |  |  |  |  |  |  |  |  |  |
| **HVG** | Seurat 3 | 0.004 | ** |  |  |  |  |  |  |  |  |  |  |  |  |  |  |
|  | MNN Correct | 0.004 | ** | 0.004 | ** |  |  |  |  |  |  |  |  |  |  |  |  |
|  | ComBat | 0.004 | ** | 0.008 | ** | 0.1 | NS |  |  |  |  |  |  |  |  |  |  |
|  | limma | 0.037 | * | 0.294 | NS | 0.004 | ** | 0.004 | ** |  |  |  |  |  |  |  |  |
|  | scGen | 0.499 | NS | 0.023 | * | 0.004 | ** | 0.004 | ** | 0.087 | NS |  |  |  |  |  |  |
|  | Scanorama | 0.004 | ** | 0.004 | ** | 0.004 | ** | 0.004 | ** | 0.004 | ** | 0.004 | ** |  |  |  |  |
|  | ZINB-WaVE | 0.008 | ** | 0.114 | NS | 0.207 | NS | 0.4 | NS | 0.042 | * | 0.02 | * | 0.008 | ** |  |  |
|  | scMerge | 0.004 | ** | 0.008 | ** | 0.209 | NS | 0.338 | NS | 0.004 | ** | 0.004 | ** | 0.004 | ** | 0.688 | NS |

**F.**

| **Down-regulated in Group 1** | **All genes** | | | | |  | **HVG** | | | | |
| --- | --- | --- | --- | --- | --- | --- | --- | --- | --- | --- | --- |
|  | **min** | **Q1** | **median** | **Q3** | **max** |  | **min** | **Q1** | **median** | **Q3** | **max** |
| Raw | 0.232 | 0.23975 | 0.27 | 0.31 | 0.324 |  | 0.401 | 0.42475 | 0.47 | 0.50925 | 0.545 |
| Seurat 3 | 0.298 | 0.33825 | 0.37 | 0.3975 | 0.408 |  | 0.613 | 0.65225 | 0.74 | 0.75775 | 0.79 |
| MNN Correct | 0.605 | 0.63825 | 0.72 | 0.72175 | 0.723 |  | 0.792 | 0.871 | 0.93 | 0.9525 | 0.964 |
| ComBat | 0.507 | 0.56125 | 0.66 | 0.6685 | 0.673 |  | 0.775 | 0.8315 | 0.84 | 0.84825 | 0.886 |
| limma | 0.584 | 0.624 | 0.68 | 0.68825 | 0.695 |  | 0.513 | 0.52875 | 0.59 | 0.72925 | 0.774 |
| scGen | 0.078 | 0.118 | 0.19 | 0.27975 | 0.356 |  | 0.307 | 0.3375 | 0.4 | 0.51875 | 0.756 |
| Scanorama | 0.114 | 0.1195 | 0.12 | 0.13 | 0.138 |  | 0.201 | 0.23025 | 0.26 | 0.26575 | 0.271 |
| ZINB-WaVE | 0.587 | 0.62225 | 0.7 | 0.70125 | 0.708 |  | 0.727 | 0.7795 | 0.91 | 0.917 | 0.932 |
| scMerge | 0.515 | 0.55625 | 0.67 | 0.68275 | 0.686 |  | 0.778 | 0.84225 | 0.87 | 0.88925 | 0.93 |

**Table A:** Accuracy metrics of true positive (TP), false negative (FN), false positive (FP), recall, precision and F-score for the six use cases comparing the true DE genes to the DE genes obtained from each corrected matrix. DEGs analysis has been calculated on all genes from simulated data or on highly variable genes (HVG), results shown on upregulated in Group 1 (**Table D**: downregulated in Group 1)

**Table B:** Pairwise comparisons of F-score from the six use cases for all methods using Wilcoxon rank sum test with Benjamini-Hochberg correction (*: p<0.05, **: p<0.01, ***: <0.001, NS: not significant). Results shown on upregulated in Group 1 (**Table E**: downregulated in Group 1)

**Table C:** Statistical values from the six use cases for all methods. Results shown on upregulated in Group 1 (**Table F**: downregulated in Group 1)
