# Supplementary material for: A benchmark of batch-effect correction methods for single-cell RNA sequencing data
Source: Genome Biol. 2020 Jan 16;21:12. doi: 10.1186/s13059-019-1850-9 (PMC6964114; doi:10.1186/s13059-019-1850-9)
Supplement: Supplementary file 9 — Additional file 9. Review history and authors’ response. Reviewers’ comments and authors’ point-to-point response. [file 13059_2019_1850_MOESM9_ESM.docx]

Review History

**First round of review**

**Reviewer 1**

**Are you able to assess all statistics in the manuscript, including the appropriateness of statistical tests used?**

yes

**Comments to author:**

Nhu et al. performed a benchmark study of computational methods for batch effect correction of single-cell RNA-seq (scRNA-seq) data. Batch effect correction is becoming a critical challenge in scRNA-seq data analysis, giving the fast-increasing volume of scRNA-seq data and the need to compare and/or integrate these data for making better informed interpretations of biological systems of interest. Benchmark studies like this one can be valuable due to the fast-growing number of methods and tools for scRNA-seq data batch effect correction. Nevertheless, I have the following comments/suggestions which may help the authors to increase the value of their benchmark of methods for scRNA-seq data batch effect correction.

1.     A key limitation of the current study is the number of datasets employed for benchmarking methods. Only three experimental datasets were used for testing the performance of different batch effect correction methods. In my opinion, this is far from sufficient. Published scRNA-seq datasets are wide-spread and easily accessible. To have a meaningful comparison, the authors need to include at least 10 or more datasets for scenarios "…non-identical cell types in two batches" and "…measured on different technologies", respectively, for performance benchmarking. The current suggestion/conclusion on which method to use is drawn on one or two dataset(s) and is highly unreliable. It therefore greatly reduces the utility of the current study.

2.     Related to the above, an appropriate statistical test should be performed to identify if any method(s) is statistically significantly better than other methods in comparison. This also requires the authors to expend the number of scRNA-seq datasets used for method comparison.

3.     The authors included nine methods for comparison (two of them are the updated versions of their original methods, i.e. "MNN" and "fastMNN"; "Seurat" and "Seurat3"). While this is a more comprehensive representation compared to the number of datasets used, I suggest the authors to also include ComBat (PMID: 16632515), scMerge (PMID: 31028141), and ZINB-WaVE (PMID: 29348443). ComBat is a well-established method for batch effect correction originally proposed for microarray data. Yet, it is found to be performing well in scRNA-seq data (see method comparison in PMID: 31028141). scMerge is another newly proposed method for scRNA-seq data normalization and integration. It uses remove unwanted variation (RUV), a well-established batch effect correction method (PMID: 25150836), as its core. ZINB-WaVE uses zero-inflated negative binomial model and represents another type of method that is not captured in the current list.

4.     The authors separated the comparison to three scenarios. I only identify two scenarios. Is differential gene identification considered as an independent scenario? I think it may make more sense to treat this as a benchmark criterion other than a scenario, since this is often considered as a downstream analysis step after batch effect correction.

5.     Three evaluation metrics are used. I would suggest the authors to also include more metrics such as Adjusted Rand Index (ARI) which is also very popular in benchmarking method performance in single cell datasets.

**Reviewer 2**

**Are you able to assess all statistics in the manuscript, including the appropriateness of statistical tests used?**

yes

**Comments to author:**

In the manuscript "A benchmark of batch effect correction methods for single-cell RNA sequencing data", the author Hoa Tran Thi Nhu et al. comprehensively compared the performance of different scRNA-seq batch correction methods. Compared with previous analysis, this research included most recent techniques as well as multiple metrics, and the result will have wide implications for biologist working on scRNA-seq analysis. To be published in Genome Biology, the manuscript still has several major points to be clarified:

1.     The authors claim they compared different data set in terms computational efficiency and the ability to handle large datasets. However, the comparison is done on a single data set with only 6,954 of cells, a small data set compared with the other studies in Scanorama (Brian Hie, et al. Nature biotechnology, 2019) or Harmony (Ilya Korsunsky, et al. bioRxiv, 2018).  Ideally, comparison across a large cell number window (1,000s to 1,000,000s) should be performed to assess the ability of different approaches in handling large datasets and computation memory usage should also be reported for comparison.

2.     "We aim to evaluate all batch effect correction approaches that have been developed for RNA-seq data". However, several commonly used batch correction methods are not included in comparison, such as Combat and Limma. It would also be nice to include other newly published techniques, such as LIGER (Joshua Welch, et al. Cell, 2019).

3.     The paper use two sets of published data sets: two blood cell data sets (Peter See, et al, Science, 2017, Alexandra-Chloe Villani, et al, Science, 2017) and two mouse atlas studies. However, neither of these two data sets are explicitly controlled in biological sample source (i.e. age, sex). As a result, this study lacks a golden standard to assess the performance of different approaches in removing technique biases or preserving real biological differences. For example, the Harmony shows lower KBET values compared with Scanorama in the first data set, but this can be due to the overcorrection of Harmony or insufficient correction by Scanorama. Ideally, the authors should evaluate different techniques in a data set from same biological source.

4.     In this research, all comparisons are done on batch correction between only two data sets. In real study, however, biologist may need to integrate three or even more data batches (i.e. Tim Stuart, et al, Cell, 2019) and this has not been covered in the manuscript.

5.     For DE gene analysis, the authors claim "most batch effect correction methods cannot preserve the shared important biological features in the two batches". However, this conclusion can be biased depending on the conditions for generating the stimulated data (i.e. low drop out events, similar cell type ratio in two batches, low cell number). Simulations with varied parameters are needed to compare different techniques in DE gene identification.

Minor comment:

6.     For assessment of batch correction algorithm, the authors use k-nearest neighbor batch estimation (kBET). The accessment of kBET depends on the value k. However, the author only choose an arbitrary k value = 30. Ideally a series of k values are needed to estimated the performance of different approaches as shown in the original paper (Maren Buttner, Nature method, 2019).

**Authors Response**

The authors would like to thank the handling editor Prof. Yixin Yao and anonymous reviewers for their valuable and constructive comments. We have carefully revised the manuscript according to the comments. In the revised manuscript, we comprehensively benchmarked 14 batch effect correction methods on 10 datasets using 5 assessment metrics. Our changes are summarized below:

1) We have increased the number of batch effect removal methods from 9 to 14 by adding ComBat, scMerge, ZINB-Wave, LIGER and limma;

2) We have increased the number of datasets from 3 to 10, and increased the number of scenarios from 3 to 5 by adding scenario 3: multiple batches and scenario 4: big data.

3) We have increased the number of assessment metrics from 4 to 5 by adding ARI.

4) We have increased the number of simulated datasets from 1 to 6 by using Splatter with varied parameters.

5) We have added UMAP visualization to the main figures and shifted tSNE visualization to supplementary figures.

6) We have added statistical tests for comparing different methods.

7) We have extensively revised the figures, Results and Conclusion sections based on our new analysis.

**Point-by-point responses to the reviewers’ comments:**

**Reviewer #1:**

Nhu et al. performed a benchmark study of computational methods for batch effect correction of single-cell RNA-seq (scRNA-seq) data. Batch effect correction is becoming a critical challenge in scRNA-seq data analysis, giving the fast-increasing volume of scRNA-seq data and the need to compare and/or integrate these data for making better informed interpretations of biological systems of interest. Benchmark studies like this one can be valuable due to the fast-growing number of methods and tools for scRNA-seq data batch effect correction. Nevertheless, I have the following comments/suggestions which may help the authors to increase the value of their benchmark of methods for scRNA-seq data batch effect correction.

Response:

We authors would like to thank the reviewer for the summary and comments of our work. Following the comments, we have revised the manuscript and detailed changes are given below.

1. A key limitation of the current study is the number of datasets employed for benchmarking methods. Only three experimental datasets were used for testing the performance of different batch effect correction methods. In my opinion, this is far from sufficient. Published scRNA-seq datasets are wide-spread and easily accessible. To have a meaningful comparison, the authors need to include at least 10 or more datasets for scenarios "…non-identical cell types in two batches" and "…measured on different technologies", respectively, for performance benchmarking. The current suggestion/conclusion on which method to use is drawn on one or two dataset(s) and is highly unreliable. It therefore greatly reduces the utility of the current study.

Response:

We thank the reviewer for pointing out this concern and fully agree that three datasets are not sufficient. In our revised manuscript, we have managed to include 7 more datasets, in total 10 datasets. As shown in Table R1 of the response letter and Supplementary Table S2 of the revised manuscript, these 10 datasets cover five different scenarios including 1) Scenario 1: identical cell types across batches measured by different technologies; 2) Scenario 2: non-identical cell types across batches measured by either the same or different technologies; 3) Scenario 3: multiple (more than 2) batches; 4) Scenario 4: big data that contain millions of cells 5) Scenario 5: six sets of simulation data with pre-defined batch effect and differential gene expression profiles generated using Splatter with varied parameters. For each scenario, we have multiple datasets. Most importantly, the 10 datasets are highly comprehensive (Table R2 of the response letter, Figure 1B of the revised manuscript), covering different species (Human, Mouse) and cell line, different tissues and cell types, 11 different single-cell RNA-seq technologies, a wide cell number range from 100s to > 500,000s, different numbers of batches (2 to 5). Based on the evaluation using these 10 datasets, we have extensively revised our figures, results and conclusion. We believe that with these modifications, our benchmarking study will be useful for single cell community in selecting an appropriate method for their batch effect removal and data integration.

Table R1. Ten datasets cover five different scenarios.

| Dataset | Description | Scenario | | | | |
| --- | --- | --- | --- | --- | --- | --- |
|  |  | 1. Identical cell types different technologies | 2. Non-identical cell types | 3. Multiple batches | 4. Big data | 5. Simulation |
| 1 | Human Dendritic Cells |  | √ |  |  |  |
| 2 | Mouse Cell Atlas | √ |  |  |  |  |
| 3 | Simulations |  |  |  |  | √ |
| 4 | Human Pancreas |  | √ | √ |  |  |
| 5 | Human Peripheral Blood Mononuclear Cell | √ |  |  |  |  |
| 6 | Cell line |  | √ | √ |  |  |
| 7 | Mouse Retina |  | √ |  |  |  |
| 8 | Mouse Brain |  | √ |  | √ |  |
| 9 | Human Cell Atlas |  |  |  | √ |  |
| 10 | Mouse Haematopoietic Stem and Progenitor Cells |  | √ |  |  |  |

Table R2: Description of ten datasets

| Dataset | Description | Number of batches | Total cell number | Technologies |
| --- | --- | --- | --- | --- |
| 1 | Human Dendritic Cells | 2 | 576 | Smart-Seq2 |
| 2 | Mouse Cell Atlas | 2 | 6,954 | Microwell-Seq |
|  |  |  |  | Smart-Seq2 |
| 3 | Simulation | Refer to Simulation table | | |
| 4 | Human Pancreas | 5 | 14,767 | inDrop |
|  |  |  |  | CEL-Seq2 |
|  |  |  |  | Smart-Seq2 |
|  |  |  |  | SMARTer |
|  |  |  |  | SMARTer |
| 5 | Human Peripheral Blood Mononuclear Cell | 2 | 15,476 | 10X 3' |
|  |  |  |  | 10X 5' |
| 6 | Cell line | 3 | 9,530 | 10X |
|  |  |  |  | 10X |
|  |  |  |  | 10X |
| 7 | Mouse Retina | 2 | 71,638 | Drop-seq |
|  |  |  |  | Drop-seq |
| 8 | Mouse Brain | 2 | 833,206 | Drop-seq |
|  |  |  |  | SPLiT-seq |
| 9 | Human Cell Atlas | 2 | 621,466 | 10X |
|  |  |  |  | 10X |
| 10 | Mouse Haematopoietic Stem and Progenitor Cells | 2 | 4,649 | Smart-seq2 |
|  |  |  |  | MARS-seq |

2. Related to the above, an appropriate statistical test should be performed to identify if any method(s) is statistically significantly better than other methods in comparison. This also requires the authors to expend the number of scRNA-seq datasets used for method comparison.

Response:

We thank and fully agree with the reviewer. In our revised manuscript, we used kBET, LISI, ASW, ARI and DEG as benchmarking metrics. For each metric, we performed **Wilcoxon rank sum test with BH correction** to identify if any method(s) is significantly better than other methods. Results of statistical tests are provided in Supplementary Table S4 and S5 of the revised manuscript. How statistical tests were applied to individual metric is described as follows. k-nearest neighbor batch estimation (kBET) metric measures batch mixing on the local level. Its value depends on neighborhood size k. In our assessment, we calculated kBET acceptance rates 5 times using five different k values and then ran statistical test. Local Inverse Simpson’s Index (LISI) was calculated on a per cell basis and then subject to statistical analysis. Average Silhouette Width (ASW) measures the similarity of a cell to its own batch or cell type compared to other batches or cell types. We sub-sampled 80% of the cells 20 times and calculated ASW cell type and ASW batch for each sub-sample which were then used for statistical test. Statistical tests of ARI were conducted in a similar manner as ASW. In the assessment using differentially expressed gene (DEG) metric, we generated six simulated datasets, computed F scores by comparing DEGs identified from batch corrected data with ground truth DEGs for each dataset, and then run statistical test on the F scores.

3. The authors included nine methods for comparison (two of them are the updated versions of their original methods, i.e. "MNN" and "fastMNN"; "Seurat" and "Seurat3"). While this is a more comprehensive representation compared to the number of datasets used, I suggest the authors to also include ComBat (PMID: 16632515), scMerge (PMID: 31028141), and ZINB-WaVE (PMID: 29348443). ComBat is a well-established method for batch effect correction originally proposed for microarray data. Yet, it is found to be performing well in scRNA-seq data (see method comparison in PMID: 31028141). scMerge is another newly proposed method for scRNA-seq data normalization and integration. It uses remove unwanted variation (RUV), a well-established batch effect correction method (PMID: 25150836), as its core. ZINB-WaVE uses zero-inflated negative binomial model and represents another type of method that is not captured in the current list.

Response:

We thank the reviewer for this comment. In our revised manuscript, we have included ComBat, scMerge and ZINB-Wave. We have also added another two methods namely LIGER and limma as suggested by the other reviewer. In total, we have included 14 different methods as listed in Figure 1A and Table 1 of the revised manuscript. We evaluated all 14 methods on 10 datasets and provided results in Figure 2-21 and Supplementary Table S4-S5 of the revised manuscript.

4. The authors separated the comparison to three scenarios. I only identify two scenarios. Is differential gene identification considered as an independent scenario? I think it may make more sense to treat this as a benchmark criterion other than a scenario, since this is often considered as a downstream analysis step after batch effect correction.

Response:

We agree that differentially expressed gene (DEG) identification should be considered as a benchmark criterion. We have revised our manuscript to highlight this point and listed DEG as one of the five benchmarking metrics in Figure 1A. In fact, DEG benchmarking needs to be run on simulated datasets, and we organized the 10 datasets and the Results section into five relevant scenarios: identical cell types, non-identical cell types, multiple batches, big data, and simulation. Thus we still presented DEG benchmarking under Scenario 5: Simulation in the Results section.

5. Three evaluation metrics are used. I would suggest the authors to also include more metrics such as Adjusted Rand Index (ARI) which is also very popular in benchmarking method performance in single cell datasets.

Response:

We agree with the reviewer. We have included Adjusted Rand Index (ARI) as one of our five assessment metrics for batch effect correction (Figure 1A of the revised manuscript). We calculated ARI for batch mixing and ARI for cell type separation_,_ then combined them into F1_ARI_ = (2 * (1 - ARI_batch_) * ARI_cell_type_) / (1 - ARI_batch_ + ARI_cell_type_). We ranked the 14 methods based on the F1_ARI_ scores. Due to the fact that each assessment metric has its advantages and limitations, we combined the rankings derived from each of ARI, ASW, kBET, LISI assessment metrics using a rank sum approach. We have provided ARI results in Figure 2-21 of the revised manuscript and discussed the effectiveness of ARI metric compared to kBET, LISI, and ASW in the Results and Discussion sections.

**Reviewer #2:**

In the manuscript "A benchmark of batch effect correction methods for single-cell RNA sequencing data", the author Hoa Tran Thi Nhu et al. comprehensively compared the performance of different scRNA-seq batch correction methods. Compared with previous analysis, this research included most recent techniques as well as multiple metrics, and the result will have wide implications for biologist working on scRNA-seq analysis. To be published in Genome Biology, the manuscript still has several major points to be clarified:

Response:

We authors would like to thank the reviewer for the summary and comments of our work. Following the comments, we have revised the manuscript and detailed changes are given below.

1. The authors claim they compared different data set in terms computational efficiency and the ability to handle large datasets. However, the comparison is done on a single data set with only 6,954 of cells, a small data set compared with the other studies in Scanorama (Brian Hie, et al. Nature biotechnology, 2019) or Harmony (Ilya Korsunsky, et al. bioRxiv, 2018). Ideally, comparison across a large cell number window (1,000s to 1,000,000s) should be performed to assess the ability of different approaches in handling large datasets and computation memory usage should also be reported for comparison.

Response:

We thank the reviewer for highlighting this issue. In the revised manuscript, we have performed the benchmarking on 10 datasets. Description of the 10 datasets is given in Figure 1B of revised manuscript and Table R2 of response letter. Data size ranges from a few hundreds to more than half a million. The 10 datasets allow us to assess the ability of different methods in handling small (< 1000 cells) to medium (1000 ~ 100,000 cells) and large datasets (> 500,000 cells).

Memory usage becomes more and more of a critical challenge when data size increases. We assessed memory usage of the 14 methods on dataset 8 which is the largest. We ran the assessment on our server with 1TB of memory and recorded memory usage every five seconds. We then visualized the memory usage in the format of violin plots (Figure 21B of revised manuscript, Figure R1A of response letter). Please take note that some methods were unable to complete the batch correction runs due to memory (> 1TB) or runtime (> 48 hours) requirements. Only LIGER, BBKNN, ComBat, Harmony, limma, ResNet, Scanorama, scGen and Seurat3 were able to complete the runs and we recorded their memory usage during the entire run; fastMNN, scMerge, Seurat2, ZINB-Wave were terminated halfway because they took more than 1TB of memory; MNN Correct was also terminated because its run time is too long.In addition to memory usage, we have also reported the runtime of the 14 methods on 10 datasets in Figure 21C of the revised manuscript and Figure R1B of response letter.

Figure R1. Memory usage and run time of batch effect normalization methods. A) Violin plots of memory usage required by each batch effect normalization method on dataset 8. B) Run time of each batch effect normalization method on each dataset. Color represents log10(Time(second)), node size represents log10(cell number).

2. "We aim to evaluate all batch effect correction approaches that have been developed for RNA-seq data". However, several commonly used batch correction methods are not included in comparison, such as Combat and Limma. It would also be nice to include other newly published techniques, such as LIGER (Joshua Welch, et al. Cell, 2019).

Response:

We thank the reviewer for this comment. In our revised manuscript, we have included ComBat, limma and LIGER. We have also added another two methods namely scMerge and ZINB-WaVE as suggested by the other reviewer. In total, we have included 14 different methods as listed in Figure 1A and Table 1 of the revised manuscript. We evaluated all 14 methods on 10 datasets and provided results in Figure 2-21 and Supplementary Table S4-S5 of revised manuscript.

3. The paper use two sets of published data sets: two blood cell data sets (Peter See, et al, Science, 2017, Alexandra-Chloe Villani, et al, Science, 2017) and two mouse atlas studies. However, neither of these two data sets are explicitly controlled in biological sample source (i.e. age, sex). As a result, this study lacks a golden standard to assess the performance of different approaches in removing technique biases or preserving real biological differences. For example, the Harmony shows lower KBET values compared with Scanorama in the first data set, but this can be due to the overcorrection of Harmony or insufficient correction by Scanorama. Ideally, the authors should evaluate different techniques in a data set from same biological source.

Response:

We thank the reviewer for highlighting this potential issue. In the revised manuscript, we have added dataset 6 and used it to address this issue. Dataset 6 contains expression data of immortalized cell lines which have no additional biological variation. As such, this dataset is particularly ideal for assessing the performance of different approaches in removing technical biases while preserving real biological differences.

Furthermore, we have worked towards achieving the gold standard by using 10 datasets that are popularly used in various batch effect correction publications for easy comparison and referencing. However, we should note that it is not easy to find datasets derived from different batches but from the same biological source. For most datasets that have batch effects to be corrected, they usually come from different labs and/or different sources/tissues/conditions.

4. In this research, all comparisons are done on batch correction between only two data sets. In real study, however, biologist may need to integrate three or even more data batches (i.e. Tim Stuart, et al, Cell, 2019) and this has not been covered in the manuscript.

Response:

We thank the reviewer for pointing this out. In our revised manuscript, we have used dataset 4 and 6 to assess batch effect correction across multiple (>2) batches. Dataset 4 consists of five batches of human pancreatic cell expression data acquired using four technologies. The distributions of cell types are significantly different between batches. Dataset 6 consists of three batches of cell line expression data. It contains only two cell types, with two out of the three batches containing only one cell type which also only shared with the third batch.

5. For DE gene analysis, the authors claim "most batch effect correction methods cannot preserve the shared important biological features in the two batches". However, this conclusion can be biased depending on the conditions for generating the stimulated data (i.e. low drop out events, similar cell type ratio in two batches, low cell number). Simulations with varied parameters are needed to compare different techniques in DE gene identification.

Response:

We thank and fully agree with the reviewer. In our revised manuscript, we have extended the number of simulations to six so that the following conditions are covered: (i) low and high drop-out events (ii) balanced and unbalanced number of cells in 2 batches (iii) very few cells in one batch. Detailed description of the six simulations are given in Figure 20B of the revised manuscript and Table R3 below. For each simulation, we compared DEGs identified from batch corrected data and ground truth DEGs to calculate a F-score (Figure 20C of revised manuscript, Figure R2 below). To statistically compare different methods, we performed Wilcoxon rank sum test with BH correction on the F-scores. Detailed information of F-scores, p-values as well as true positive, false negative, recall and precision are provided in Supplementary Table S5 of our revised manuscript. Please note that we have also added DEG identification results of three new methods scMerge, Limma, Zinb-WaVe.

Table R3. Six datasets simulated using varied parameters.

Figure R2. DEG identification F scores calculated by comparing DEGs identified from batch corrected data with ground-truth DEGs.

Minor comment:

6. For assessment of batch correction algorithm, the authors use k-nearest neighbor batch estimation (kBET). The accessment of kBET depends on the value k. However, the author only choose an arbitrary k value = 30. Ideally a series of k values are needed to estimated the performance of different approaches as shown in the original paper (Maren Buttner, Nature method, 2019).

Response:

We agree that the assessment of kBET depends on the value k. And we also noticed when k is too small or too big, kBET is unable to differentiate the performance of good batch correction methods from poor ones. In order to decide an appropriate range of k values, we performed computational simulation to evaluate the effect of k values on kBET. We simulated 11 sets of data comprising 2 batches b1 and b2. Each batch has variance equal to 1 and distance between mean of b1 and mean of b2 ranges from 0.25 to 4 (Figure R3A). We ran kBET using varied k value ranging from 1% to 100% of the total cell count and plotted kBET acceptance rate, i.e. 1- rejection rates (Figure R3B). As expected, the further the two batches are apart, the lower acceptance rate is. When k value increases, kBET acceptance rate first decreases and then increases. **Notably, when k is greater than 25% of total cell count, dataset with distance=0.3 has higher acceptance rate than distance=0.25, which is not correct.** Therefore, we have re-run kBET for all 14 methods on 10 datasets using k=5%, 10%, 15%, 20%, 25% of total cell count, and taken the average kBET acceptance rates over different k to rank the 14 methods. kBET results for datasets 1, 2, 4, 5, 6, 7, 8, 9, 10 are given in Figure 3, 5, 7, 9, 11, 13, 15, 17, 19 of the revised manuscript. An example of kBET plots is provided in Figure R4 of response letter.

Figure R3. kBET analysis using simulated datasets to determine an appropriate range of k value. A) Dot plots of 11 simulated datasets with different distance between the two batches. Dots are colored by batch. B) kBET acceptance rates calculated using different k values on the 11 simulated datasets.

Figure R4. kBET acceptance rates of the 14 methods calculated on dataset 4 using a series of k values being 5%, 10%, 15%, 20% and 25% of the total cell count.
